# Replacing Sanger with Next Generation Sequencing to improve coverage and quality of reference DNA barcodes for plants

**DOI:** 10.1038/srep46040

**Published:** 2017-04-12

**Authors:** Mike J. Wilkinson, Claudia Szabo, Caroline S. Ford, Yuval Yarom, Adam E. Croxford, Amanda Camp, Paul Gooding

**Affiliations:** 1 Pwllpeiran Upland Research Centre, Institute of Biological, Environmental and Rural Sciences, Aberystwyth University, Ceredigion, SY23 4AB, UK; 2 School of Computer Science, The University of Adelaide, SA 5005, Australia; 3 School of Agriculture, Food and Wine, Waite Campus, The University of Adelaide, SA 5064, Australia; 4 School of Animal & Veterinary Sciences, Roseworthy Campus, The University of Adelaide, SA 5371, Australia; 5 Australian Genome Research Facility, Plant Genomics Centre, Hartley Grove, Urrbrae, SA 5064, Australia

## Abstract

We estimate the global BOLD Systems database holds core DNA barcodes (*rbcL *+* matK*) for about 15% of land plant species and that comprehensive species coverage is still many decades away. Interim performance of the resource is compromised by variable sequence overlap and modest information content within each barcode. Our model predicts that the proportion of species-unique barcodes reduces as the database grows and that ‘false’ species-unique barcodes remain >5% until the database is almost complete. We conclude the current *rbcL *+* matK* barcode is unfit for purpose. Genome skimming and supplementary barcodes could improve diagnostic power but would slow new barcode acquisition. We therefore present two novel Next Generation Sequencing protocols (with freeware) capable of accurate, massively parallel *de novo* assembly of high quality DNA barcodes of >1400 bp. We explore how these capabilities could enhance species diagnosis in the coming decades.

Specimen identification by DNA barcoding provides an attractive alternative to morphological examination in large-scale studies^1^ and works on cryptic^2^, microscopic^3^ or digested materials^4^. Species identification of an unknown sample is achieved by comparing its DNA barcode against reference sequences from authenticated specimens of that species. Agreement on standard barcoding loci has been central in the drive towards the harmonization of DNA barcoding approaches. The two core barcode loci designated for land plants are *rbcL* and *matK*^5^; both coding regions on the chloroplast genome. The diagnostic power of these markers is in part determined by context. For instance, identification of a specimen from a species-poor plant community naturally poses a far lesser challenge than one from a species-rich location or an island flora that has undergone extensive species radiation and/or hybridization^6^. The effectiveness of the core barcode across a wide range of user scenarios is determined by the properties of the core barcode itself and crucially, by the availability of reference DNA barcode sequences against which unknown samples can be compared.

A public database platform, BOLD Systems was created in 2005 to provide users with quality-assured and specimen-referenced DNA barcodes from around the world^7^; a move that benefited many research fields^8^,^9^,^10^,^11^. Since then, BOLD Systems has accumulated barcodes for 20% (68,823/350,699) of land plants^12^ (www.boldsystems.org, May 2016). However, not all species records contain both core barcodes (*rbcL* and *matK*). We surveyed 1,000 plant species covering all major taxonomic groups and found 75% contained both *rbcL* and *matK* (Supplementary Table S1). If this pattern applies generally, species-level coverage of the complete core barcode is around 15% (51,410/350,699) of land plant species. New species records with both core barcodes have therefore accumulated at 4,674 pa (51,410 species in 11 years); requiring >64 years for completion of all land plant species (299,289 species @ 4,674 species pa). In reality a far longer completion period is more likely because early progress was accelerated by extensive sequence mining from other databases (75% of all records, www.boldsystems.org, May 2016) and natural bias towards the common and least problematic species. Moreover, as time passes it will become increasingly difficult to find wanted or missing species from the BOLD database such that it is likely to take considerably longer than the above estimates before the BOLD database approaches complete species coverage. Current practice also emphasizes taxonomic coverage over improved representation of intraspecific variation^13^, such that species records are commonly represented by one or few individuals^14^,^15^. For the foreseeable future therefore, species coverage and intraspecific variation will be very incomplete.

## Results

### Limitations of an incomplete global reference barcode database

Barcode read lengths are variable on BOLD Systems and thus the number of bases that can be compared in larger alignments may be reduced. We explored this phenomenon by aligning variable numbers of barcodes from 650 records of 101 congeneric species pairs recovered from BOLD Systems (Supplementary Table S2). Shared sequence overlap of *rbcL* alignments declined to <200 bases for alignments comprising >400 barcodes (Supplementary Fig. S1). This reduced effective information content to less than a third of the full *rbcL* locus. For *matK*, shared sequence overlap declined more steeply and assemblies containing >100 specimens failed altogether (Supplementary Fig. S1). Removal of all reads <300 bp in length slightly reduced the decline (Supplementary Fig. S1), with the small cost of excluding 2% of records. Increasing the filter length to 500 bp (for *rbcL*) and 600 bp (for *matK*) reduced the decline further but increased record losses to 8% (85/1056) and 10% (113/1154) respectively. Thus, the effective size of the database is far smaller than simply the number of records it contains. We next examined how database incompleteness affects performance.

To explore how the diagnostic properties of an incomplete reference database change during expansion, we constructed an artificial Comprehensive Reference Barcode (CRB) database to represent the entire flora of a hypothetical region. For this we retrieved *rbcL* and *matK* sequences from 721 complete chloroplast genomes spanning land plant phylogeny (Supplementary Table S3). Use of chloroplast genomes avoided confounding variation in locus coverage. We introduced intraspecific variation using barcodes recovered from BOLD Systems. The model (IRB Assembly) then mimicked the unpredictable pattern of species assimilation into the reference barcode resource. For this we created a series of incomplete reference databases at various stages of completion. The intention was to create a model system analogous to the partial but growing species coverage in BOLD Systems as measured against the true global picture of barcode sequence diversity (CRB in the model). This was achieved by creating 200 independent Incomplete Reference Barcode (IRB) sets from the CRB, with each replicate set containing the same number of species (N_s_, where 70 < N_s_ < 735). The IRBs contained randomly selected individuals from the CRB (with replacement) until N_s_ was reached. In the model, IRBs therefore represent a range of possible reference datasets that could occur at different stages of completion (for example, the range of reference barcode entries in the database when species coverage reached 50%). In contrast, the CRB represents all barcodes and species names that exist in the hypothetical region (i.e. a total view of all variation); a feat that would be impossible in a real biological system.

We used the model IRB Size to characterize changes to *rbcL* and *matK* performance as IRBs grew. The model deemed barcodes as ‘perceived species-unique’ (barcodes with only one species name in the IRB), ‘ambiguous’ (>1 species sharing the same IRB barcode) or ‘false species-unique’ (barcodes that are species-unique in the IRB but **not** in the CRB). The ‘false species-unique’ type of misclassification will inevitably lead to inflated estimates of the ability of a barcode to distinguish between species. It is therefore important to carefully characterize changes in its abundance as a reference barcode database grows.

**Figure 1:**
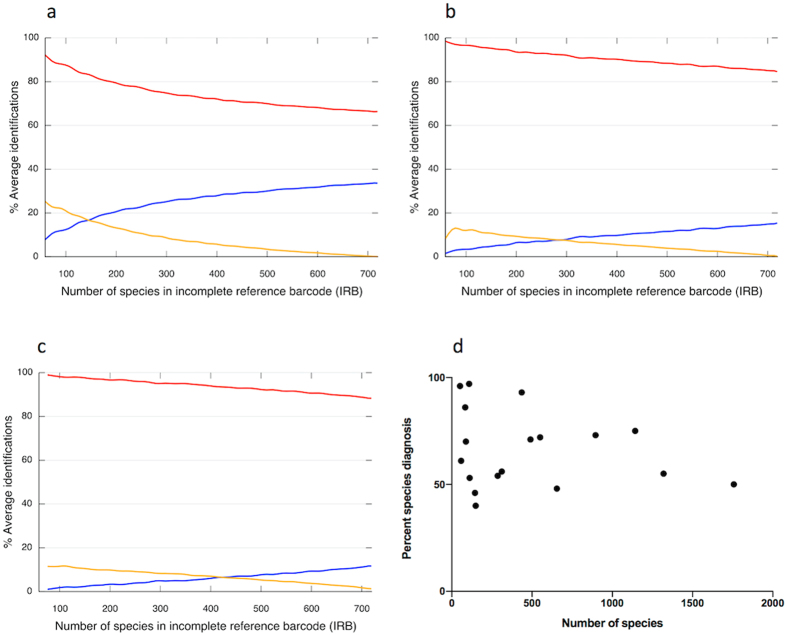
Changing frequency of species-specific DNA barcodes in an expanding reference resource. A model constructs a comprehensive reference barcode (CRB) resource from *rbcL* and *matK* sequences from 721 whole chloroplast genomes and associated variants derived from the BOLD Systems database to represent all barcodes in a hypothetical geographic area. The model takes subsets of barcodes from the CRB to represent incomplete coverage of barcodes in the reference database (Interim Reference Barcode, IRB) and records the proportion of barcodes that are perceived as species-specific (red line), ambiguous (blue line) or false (species-specific in the IRB only, orange line) for: (**a**) *rbcL*, (**b**) *matK* or (**c**) *rbcL* + *matK*. Panel (**d**) shows changing frequency of species-specific DNA barcodes with expanding species coverage recovered from literature listed in Supplementary Table S4.

For *rbcL*, almost all barcodes were perceived species-unique in small IRBs but the proportion fell as the IRB expanded, according to the curve y = 104.37x^−0.12^ (Fig. 1a). When the IRB reached CRB size, only 66% of *rbcL* barcodes were species-unique (Fig. 1a). Ambiguous barcodes were almost absent when the IRB was small but increased as the IRB grew (Fig. 1a,c). By comparison, 20–30% of *rbcL* records were false species-unique in the smallest IRBs only falling gradually to <5% when the IRB exceeded 58% (427/735 species) of CRB’s size (Fig. 1a). *MatK* exhibited a slower decline in perceived species-unique barcodes (y = 98.649e^0.004x^), falling to 85% when IRB equalled CRB size (Fig. 1b). The combined *rbcL *+* matK* locus performed best with slowest decline (y = 99.188e^−0.003x^), finally reaching 88% species-unique barcodes (Fig. 1c). False species-unique barcodes were initially less frequent for *matK* and *rbcL *+* matK* but the decline was slower and levels only fell below 5% when the IRB reached 59% and 72% of CRB size respectively (Fig. 1b,c). Resolution improved at the genus level but similar patterns were noted for both loci as the IRB expanded (Supplementary Fig. S2). This predicted decline in barcode performance with IRB expansion is mirrored in the literature by a slow but erratic decline in species identification with increasing size of *de novo* barcoding studies, despite some variation in methodology used for species designation (Fig. 1d, Supplementary Table S4).

The IRB Size model represents best possible levels of diagnostic performance. Identifying unknown specimens by comparison to an incomplete reference database introduces additional complexity because query species may be missing from the reference dataset. We simulated an Ecological Study Set (ESS) by randomly taking samples from the CRB (with replacement) to a specified number of species. We then attempted to identify each ESS specimen, initially by using exact matches to records in the IRB to best reveal sources of error. ESS barcodes that uniquely matched barcode(s) of one species in the IRB were deemed ‘perceived’ identifications. Specimens with barcodes shared by >1 IRB species were deemed ‘ambiguous’. However, samples designated ‘perceived’ in the IRB but ‘ambiguous’ in the CRB were deemed ‘false’ (false positive identifications). Those in concordance were labelled ‘true’. Failed matches were simply ‘unknowns’.

**Figure 2:**
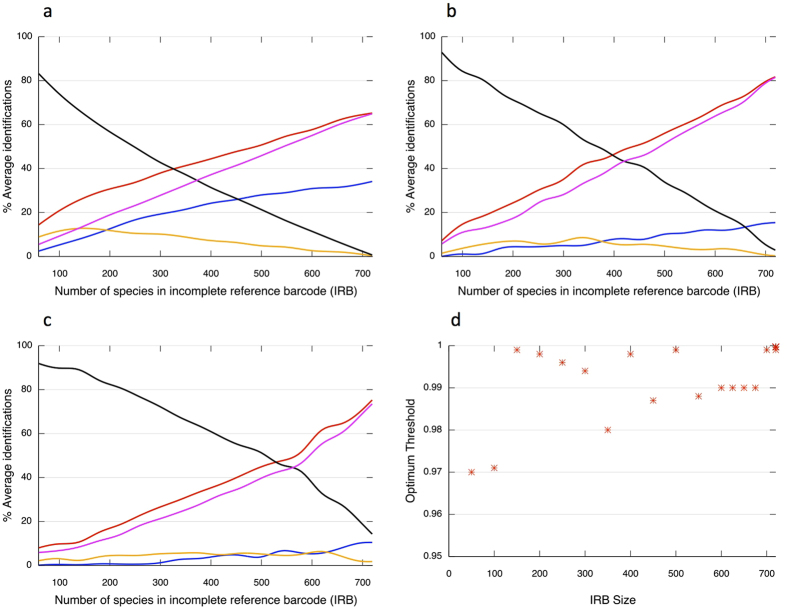
Species identification accuracy for unknown samples matched to an incomplete reference DNA barcode resource. A model constructs a comprehensive reference barcode (CRB) resource from *rbcL* and *matK* sequences from 721 whole chloroplast genomes and associated variants derived from the BOLD Systems database to represent all barcodes in a hypothetical geographic area. The model then takes subsets of barcodes from the CRB to represent incomplete coverage of barcodes in the reference database (Interim Reference Barcode, IRB) and also a random set of unknown samples from the CRB to represent an Ecological Study Set (ESS). The panels above indicate the various outcomes: perceived identifications (red line); ambiguous (blue line); unknowns (black); true (pink); false (orange) when identification of samples from the ESS (200 species) is attempted by exact matches made to IRBs of various sizes for: (**a**) *rbcL*, (**b**) *matK,* and (**c**) *rbcL* + *matK*. The model then allowed for 0.1–3% sequence similarity threshold for matches between ESS query samples and the IRB reference barcode, and calculated the Optimum threshold (O_t_) for each IRB size. Panel (**d**) shows how O_t_ changes with IRB size.

IRB size profoundly altered performance of both barcodes. Unknowns dominated when IRBs were <40% of the CRB size (Fig. 2a,c), highlighting the importance of accommodating for unsampled variation when using incomplete reference databases. For *rbcL*, perceived identifications initially comprised <20% of samples, around half of which were false (Fig. 2a). Perceived, true and ambiguous identifications rose steadily with IRB size, with true diagnoses culminating at around 55% (Fig. 2a). False identifications declined slowly and fell below 5% when the IRB contained 69% (500/721) of species in the CRB (Fig. 2a). IRB size had greater effect on *matK*, with lower perceived identifications for small IRBs but a higher final level of true diagnoses (>60%), and unknowns only falling below 50% when the IRB was half of the CRB size (Fig. 2b). False identifications were lower for *matK*, but still represented 25–40% of perceived identifications until the IRB was around 30% CRB-size (Fig. 2b). For the combined barcode there was little change to the progression of the perceived and true identifications of *matK*, although true identifications ultimately exceeded 70% (Fig. 2c). ESS size had little impact on these trends (Supplementary Fig. S3), indicating that reference resource size has greatest influence on exact match identifications.

The exact match approach to species identification does not accommodate for unsampled variation. This probably explains the dominance of unknowns in IRBs of comparable completeness to current coverage in BOLD Systems. The simplest way to address this problem is to allow for some degree of mismatch between query and reference sequences. We therefore repeated the combined barcode (*rbcL *+* matK*) run but with variable sequence similarity thresholds (97–99.9%) to define species matches between ESS and IRB barcodes. Optimal similarity threshold (O_t_) was not stable but increased stochastically as the IRB grew (Fig. 2d).

### Next Generation reference barcoding as a possible solution

**Figure 3:**
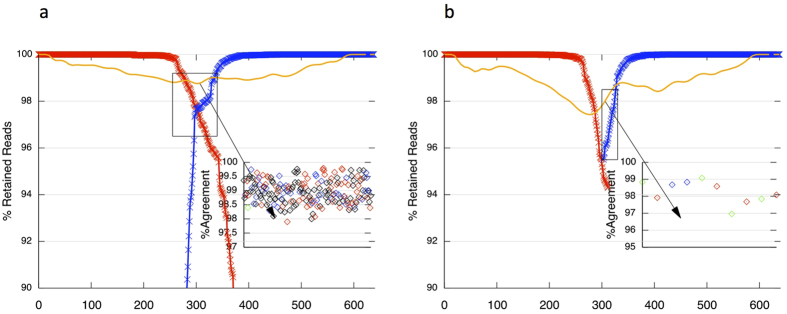
Extended Illumina MiSeq reads for high throughput *rbcL* reference barcoding. The standard protocol of the Illumina V3 MiSeq 2 × 300 kit was changed from a bidirectional to a unidirectional format (Extended Unidirectional Sequencing, EUS). The panels above indicate the proportion of EUS reads passing filter in the forward (red line) or reverse (blue line) directions only, and show the percentage consensus agreement (orange line) at each base position for: (**a**) the best example profile for *rbcL (Rhagodia spinesens* X98); (**b**) the worst example profile for *rbcL (Eremophila scoparia* M56B). Inset shows base-by-base consensus agreement in boxed area.

We sought to circumvent the current reliance on the relatively slow Sanger sequencing method for reference barcode acquisition by modifying the Illumina V3 MiSeq 2 × 300 bp protocol. The strategy (Extended Unidirectional Sequencing, EUS) first involved swapping bidirectional reads across all colonies for extended unidirectional reads. Rare colonies containing extended reads in either the forward or reverse direction were isolated and merged to form a consensus sequence. This approach yielded merged *rbcL* reads for 93% (110/118) of samples (Supplementary Table S5). All successful reads were full-length (599 bp), with a mean overlap between forward and reverse reads of 116 bases (range 10–196) (Supplementary Table S5). All EUS profiles provided >100-fold coverage throughout the read (compared with 1–2x coverage for all Sanger barcodes) and achieved base-by-base consensus agreement of >97% (Fig. 3a,b). The high level of consensus simplified merging of forward and reverse sequences. Error rates (non-consensus base calls) in successful reads were lowest (<1%) proximal to primer binding sites and highest (<3%) in the area of overlap between forward and reverse reads (Fig. 3a,b). All failures occurred where there were insufficient reads passing filter to maintain 100-fold coverage into the overlap area. This EUS method therefore allows for large-scale generation of *rbcL* barcodes with existing primers and can be used for *COI* or presumably for a single copy nuclear barcode of similar size.

**Figure 4:**
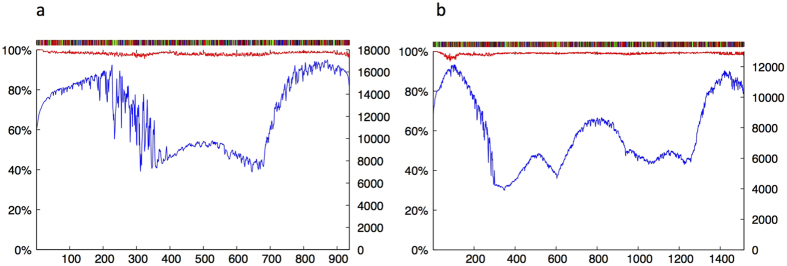
Sonication-MicroAssembly (SMA) method for high throughput *matK* reference barcoding. Amplicons of the plant DNA barcode locus *matK* were sonicated and tag-labelled for each specimen prior to sequencing on the Illumina V3 MiSeq platform. Sequences sharing the same species tag label were re-assembled. The pipeline used by the freeware removes the tag labels and aligns all sequences passing filter along a sliding window until the two sequences can be merged. The panel (**a**) above shows an example readout indicating the consensus barcode sequence across the barcode region (upper bar, where black = G; green = A; red = T and blue = C), percent consensus agreement for all reads passing filter across the 950 bases of *matK* (red line) and the number of individual sequences contributing to each base position (blue line) for *Callitris rhomboidea*. Panel (**b**) shows the same plot for near gene-length (>1,400 bases) reads of *matK* recovered for *Rytidosperma caespitosa* (M3).

We next explored a Sonication-MicroAssembly (SMA) strategy for *de novo* barcoding of *matK* and longer barcodes. Here, barcode amplicons were sonicated and the fragmented products tag-labelled during library preparation. The reconstruction of sequences into a consensus recruited up to 100,000 reads (depending on the sample) of which 82% (min 63%, max 95%) were used in making an alignment across the entire barcode region. Unlike the EUS method, error rates (<4% base call consensus) were evenly distributed and not greatly influenced by position in the alignment (Fig. 4a). SMA thereby allowed us to recover 92% (88/96) full-length *matK* barcodes (mean 899 bp, range 856–922 bp) with 96–99% consensus sequence support at all base positions (Fig. 4a, Supplementary Table S5). This compares with 6% (5/87) full length reads (between primers) and a mean read length of 873 bp (801–905 bp) using Sanger sequencing (Supplementary Table S5). We conclude that the SMA method, either alone or in combination with the simpler EUS method allows more rapid assembly of full-length core plant barcodes than Sanger using existing primers. Furthermore, read redundancy provides valuable additional information on sequence accuracy.

**Figure 5:**
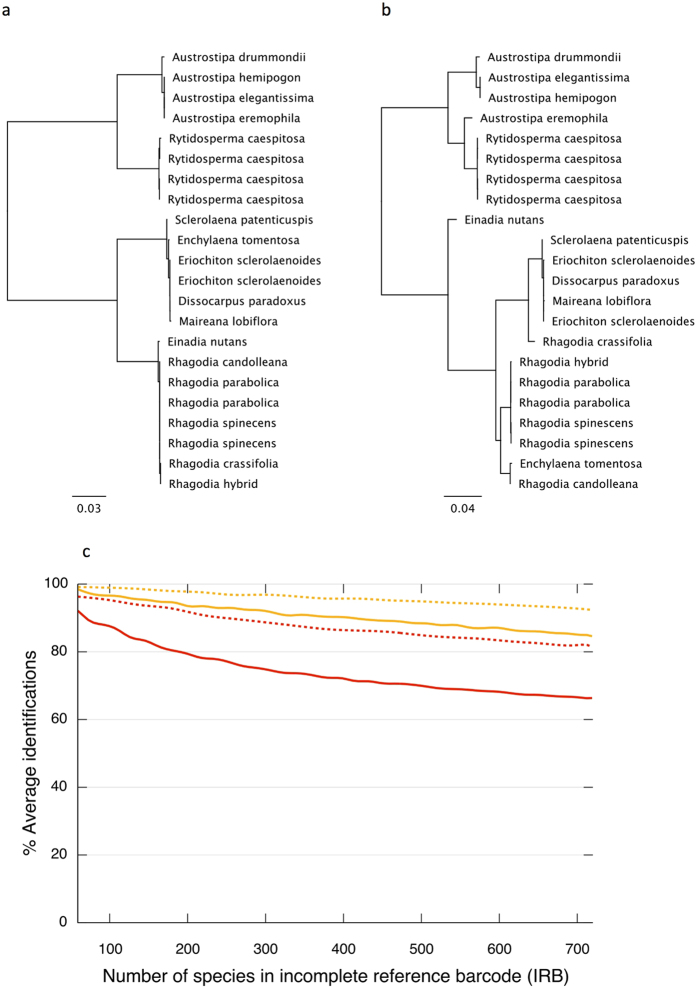
Improved diagnostic performance of gene-length barcoding for *rbcL* and *matK*. Species separation of 16 problematic species from two families (Poaceae and Amaranthaceae) as illustrated by nearest neighbour trees generated using Geneious software with (**a**) Sanger sequences of the standard core barcode (*rbcL* + *matK*) and (**b**) near gene-length sequences of *rbcL* and *matK* generated using the Sonication-MicroAssembly (SMA) method. Panel (**c**) provides a plot produced using the IRB Size model (as depicted in Fig. 1) that compares the changing diagnostic performance of extended read (1,400 bases) barcodes (dashed lines) possible using the SMA method with existing full-length barcodes (solid lines) across 721 species as the Interim Reference Barcode (IRB) database increases in size for *rbcL* (red) and *matK* (orange).

Given that the SMA method has potential for increased read lengths, we attempted to retrieve gene-length sequences for both barcode genes from 16 species of two families (Poaceae and Amaranthaceae) including one suspected hybrid. We recovered near gene-length sequences for *rbcL* (1,333 bp excl. primers) and for *matK* (1,456 to 1,471 bp excl. primers), with >100 fold coverage and >97% base-by-base consensus agreement for all reads (e.g. Fig. 4b, Supplementary Fig. S4). The increased information content significantly improved resolution over standard Sanger barcodes (Fig. 5a,b) and was able to provisionally identify the maternal parental species of the suspected hybrid as *Rhagodia parabolica* (Fig. 5b). Re-running the IRB Size model provided a more general picture of the scale of improved resolution. The extended barcodes showed marked improvement across all IRB sizes and culminated in a 16% increase in species-unique sequences (to 82%) for *rbcL* and an 8% increase (to 92%) for *matK* when the IRB reached CRB size (Fig. 5c).

## Discussion

There are no universally agreed primer binding sites for the two core plant barcode loci *rbcL* and *matK*^16^,^17^,^18^; and there is a variable need to trim individual sequences for quality assurance following Sanger sequencing. This results in reference barcodes that have considerable length variation in BOLD, which in turn reduces the proportion of sequence overlap between records; an effect which is compounded as larger numbers of barcodes are compared. This feature is more pronounced in the faster-evolving *matK* and could significantly impede the usefulness of BOLD for larger scale ecological studies. For *rbcL*, we found that shared overlap in alignments of >400 records was reduced to less than a third of the full barcode locus. This is far lower than is typical for self-contained studies, where methodology and primer selection are usually harmonized^19^,^20^ or adapted for the study group^16^,^17^ and where short reads are often removed^17^. The effect was even more pronounced for *matK*, where comparisons between >100 specimens usually failed altogether. The simplest means of circumventing this problem is to remove short barcode sequences from these alignments but this resulted in 8% reduction of *rbcL* records and 10% for *matK*, and frequently meant greater proportions of species were lost from multi-species alignments. This ‘alignment effect’ means that the effective species coverage of BOLD is even smaller than it appears on the basis of species counts alone.

One unfortunate feature of an expanding global database is that the proportion of species that can be distinguished using the core barcodes may alter as the number of species it contains increases. Using models based on sequences retrieved from chloroplast genomes (to remove any confounding variation through variable barcode length) the number of species-specific barcodes using *rbcL, matK* and the combined locus invariably declined as the reference barcode resource expanded. It was possible to generate good fits to each of these decay curves. However, such is the scale of the difference between the hundreds of barcodes used here and the 350,699 species estimated on earth^12^, the error terms preclude a robust extrapolation to a completed global reference barcode resource. We can nevertheless infer the ultimate diagnostic value of *rbcL *+* matK* is likely to be far lower than any current estimates made from studies of hundreds to a few thousand species^5^,^16^,^17^,^20^,^21^,^22^.

There is a second, more direct problem of using an incomplete reference barcode resource for specimen identification. Large-scale ecological studies wishing to identify specimens by reference to an incomplete reference database face the risk that exact matches are unlikely because of incomplete species and intraspecific coverage. Many works accommodate for this limitation by permitting a certain amount of variation between the query and reference barcodes. One of the advantages of modelling a comprehensive barcode resource is that the performance of an arbitrary similarity threshold value used on an incomplete data set can be directly tested against the ‘true’ answer in the comprehensive set. Thus, when we modelled the effect of expanding an incomplete reference barcode data set, we were able to show that the optimal similarity threshold (O_t_) was not stable but increased stochastically with dataset size. This implies that not only will there be no universal O_t_, a feature already noted between taxonomic groups^23^,^24^, but also that O_t_ changes as the reference database expands. In part this is because both intra- and interspecific variation expands as the IRB grows.

The Best Close Match method of sample assignment^25^ allows for variable intraspecific variation using frequency distribution estimates of intraspecific pairwise distances. These estimates are used to impose threshold distances for query to reference barcode matches and are intended to capture a fixed proportion of conspecific variation. The popular method is viewed as robust^26^,^27^ but is sensitive to poor or skewed representation of intraspecific variation, as is generally true for species in BOLD Systems^14^,^15^. Accelerated intraspecific representation is therefore needed for the database to adequately serve the many studies that use this system. Other workers have elected to use tree-based approaches to identify query barcodes^16^,^19^,^28^. However, concerns over these approaches centre on reliance for the queried sample to cluster within supported clades for species assignment, an inability to assign a ‘no identification’ result^27^ and sensitivity of the approach to incomplete lineage sorting and species-level paraphyly^29^. These properties imply that tree-based approaches are best deployed when the reference database is nearly complete (i.e. IRB approaches CRB size) for the research question being addressed. Thus, regardless of which method is used to accommodate unsampled variation, performance of BOLD Systems is tightly associated with its inter- and intraspecific coverage. Acceleration towards a comprehensive global barcode database is therefore imperative. Even then, poor current performance, variable read lengths and our projections of diminishing diagnostic value of *rbcL *+* matK* as the database expands implies that the current core plant barcode may ultimately prove unfit for purpose. The accumulation of supplementary barcode loci to increase the information content of the core barcodes has therefore attracted considerable support^5^,^30^,^31^,^32^. The problem here is that species coverage of all additional loci is currently poor in BOLD Systems and addressing the shortfall would slow progress towards comprehensive coverage still further. *We therefore reason the needs are for accelerated accumulation of high quality, full-length reference barcodes AND to extend the read lengths of current core barcodes such that their diagnostic power is significantly improved.*

Acquisition of new reference barcodes is currently limited by reliance on Sanger sequencing^33^. Coissac and colleagues^34^ proposed that genome skimming^35^,^36^ (low coverage shotgun sequencing of genomic DNA) could complement Sanger-based barcoding and improve the information deficit of the core barcode. Genome skimming exploits high abundance of plastid DNA within genomic DNA samples to allow full assembly of entire chloroplast genomes whilst also providing thin nuclear genome coverage. This PCR-free strategy avoids primer design problems and allows simultaneous acquisition of all plastid barcodes at once. Furthermore, superficial coverage of the nuclear genome may assist the search for nuclear barcode(s). Unlike organelle markers, nuclear markers do not typically show uniparental inheritance^34^ and so could help identify scenarios where hybridization has led to all organelle markers being ‘captured’ by a cross-compatible species. In such cases, no organelle marker would be species-specific and so misidentifications more likely^34^. However, the genome skimming strategy is expensive, requires extensive computational resources and would initially be restricted to well-resourced projects handling a few thousand samples^34^. It is therefore ill-suited to accelerate new barcode acquisition. The slow speed of Sanger sequencing also drives current practice of using a single bidirectional read per barcode entry and means consensus agreement between technical replicates cannot be used to assess base call accuracy.

First use of Next Generation Sequencing for *de novo* barcoding deployed a 454 pyrosequencing pipeline to secure full-length (658 bp) *COI* barcodes for 189 of 190 Lepidopteran specimens^33^. No base-by-base assessment of sequence consensus was provided but a mean of 143 reads per target demonstrated its feasibility. However, for this protocol to be used for reference barcode generation, propensity of the 454 platform for homopolymer length inaccuracies^37^ warrants consideration. This feature of the technology^37^ means consensus agreement across such arrays provides little indication of base call accuracy and seriously impedes its usefulness for new reference barcode accumulation. Chemistry on the Illumina NGS platforms is apparently free from this incongruity and although the methodology can also struggle with repeat motifs^38^, sequencing error rates are low^39^. Nevertheless, read lengths are currently too short to yield full-length barcodes directly^18^. Shokralla and colleagues^18^ overcame the shortfall between the 600 base reads of the Illumina V3 MiSeq 2 × 300 bp kit and the 658 bp in the *COI* locus by paired-end sequencing of two overlapping *COI* amplicons, and merging reads to assemble full-length barcodes. This solution, whilst effective, requires additional primers for all barcode loci across taxonomic groups. This would be no small undertaking given the current lack of universal primers for plants^16^,^17^,^40^ and wide variance between groups^41^, and could easily offset gains made through accelerated sequencing.

In this study we present two alternative approaches for *de novo* reference barcoding using existing Next Generation Sequencing platforms. The EUS method allows for large-scale generation of *rbcL* barcodes with existing primers (600 bp) and may be used for *COI*, or presumably a single copy nuclear barcode of similar size. The relatively few failures arose in samples with insufficient reads to maintain >100-fold coverage into the overlap region. This limitation could be managed by reducing the number of different samples included in a single run. However, the method cannot assemble full-length *matK* reads (approx. 900 bp) pending further improvements to Illumina read length or until other long-read NGS technologies (e.g. PacBio^42^ or Oxford Nanopore^43^) are able to generate equivalent quality scores at low cost^44^,^45^. As an alternative the SMA method presented here enables the generation of sequences up to 1.5 Kb in length, and offers the potential for more. The approach appeared to be insensitive to individual read lengths and the few errors generated were randomly dispersed. This method therefore enables the acquisition of full-length *matK* barcodes and/or near gene-length sequences for both *rbcL* and *matK* in a high throughput system. However, such measures alone will not overcome all limitations of the current core barcode. There will inevitably remain some groups that cannot be separated into species even following the extension of the core barcode to encompass near full-length reads of *rbcL* and *matK*. For example, Seberg and Petersen^46^ calculated even 5,800 bp from six plastid barcode regions were insufficient to separate all 86 known species of *Crocus*.

Widespread and systematic adoption of the NGS pipelines outlined here could nevertheless help address many of shortcomings of the current DNA barcode reference resource. Our pipeline provides an opportunity for a global drive to produce high quality and up to gene-length reference barcodes for land plants which could supplement and then eventually replace variable Sanger barcodes. This would: (i) accelerate progress towards comprehensive species coverage; (ii) provide base-by-base measures of call accuracy of all records via consensus agreement; (iii) improve the modest diagnostic performance of the *rbcL *+* matK* core barcode; (iv) reduce alignment issues for large sample sets; (v) allow rapid introduction of single copy nuclear barcodes, and (vi) help harmonize the data needs of the DNA barcoding and systematics communities. Viewed from a broader perspective, there is a developing dichotomy in the barcoding community between users that prefer to use genome skimming approaches^34^,^35^,^36^ for specimen identification and those that use conventional (Sanger) barcodes^16^,^17^,^18^,^19^,^20^. The former often generates whole plastid sequence information and nuclear genome information from multiple, unlinked loci. It therefore provides much increased resolution for species separation. However, the nuclear data is thin, untargeted and scattered and is ill-suited for incorporation into a reference barcode database. In contrast, the current use of two plastid barcode loci is relatively cheap and easy to assemble into a useable reference barcode resource but as we have demonstrated in the present work, probably lacks the information content to address the needs of all users, even if gene-length core barcodes were deployed. In the longer term, we therefore advocate the addition of a small number of conserved nuclear barcode loci to the extended length *rbcL* + *matK* core barcode. This would address the short-comings of both systems by allowing massively parallel generation of high quality multi-locus reference barcodes from several species simultaneously. However, this approach is predicated on the development and deployment of suitable nuclear markers and so in the short term, attention should probably focus on accelerating provision of core barcode reference resources. There are several logistical challenges that would need to be addressed before significant progress could be made to meet this, less ambitious goal. These include (among others) agreement on primers, standardization of sequence error-calling protocols, specimen archiving and PCR protocols (to minimize amplification-derived errors). Moving forward, if the high throughput afforded by adopting an NGS approach to *de novo* barcoding is to have lasting benefits, greater emphasis needs to be placed on the accuracy of specimen identification as well as on sequencing, and on the capacity to remove erroneous records from the repository. In our view, it is important to take advantage of these developments and implement a more measured acceleration towards a lasting resource of high quality, informative DNA barcodes that are fit for purpose.

## Methods

### Modelling

Methods used in this study to estimate the proportion of species in BOLD Systems database containing both *rbcL* and *matK* and examine the effect of variable sequence overlap in multi-specimen alignments from BOLD Systems are described in Supplementary Methods S1.

The models developed to assess the importance of incomplete species coverage in the reference database, to simulate the process of building a reference barcode database from scratch, and to characterize changes to the diagnostic properties of *rbcL* and *matK* as the reference resource expands are described in Supplementary Methods S2.

Real-world identification using DNA barcodes requires comparison between unknown specimens and reference barcodes. To model this, we simulated variable collections of field specimens requiring identification as described in Supplementary Methods S3.

### Reference barcoding

#### Plant materials and DNA extraction

Plant samples were collected from the Murraylands region of South Australia during February-March 2013 and October 2014 (Supplementary Table S5). Specimens were identified by reference to the Flora of South Australia^47^, and Atlas of Living Australia (www.ala.org.au). Herbarium specimens were generated and lodged with the State Herbarium of South Australia. Diversity of the sample set was increased by the addition of nine Gymnosperm species (for which *rbcL* and *matK* data were available in BOLD) from the Waite Arboretum (University of Adelaide, South Australia). DNA was extracted using the Qiagen 96 Dneasy Plant DNA kit or Bioline Isolate II Plant DNA extraction kit according to the manufacturers’ instructions.

#### Sanger sequencing

PCRs were performed with Biomix *taq* polymerase using standard reaction mixtures as recommended by the manufacturer (Bioline). For thermocycling *rbcL* primers *rbcLa*_f^48^ and *rbcL*a_rev^49^ were applied with: 95 °C for 4 min; 5 cycles of 94 °C 30 s, 55 °C 1 min, 72 °C 1 min; 30 cycles of 94 °C 30 s, 54 °C 1 min, 72 °C 1 min; and 72 °C for 10 min. For *matK* primers xF^29^ and MALPR1^41^ were applied with: 95 °C 4 min; 10 cycles of 94 °C 30 s, 52 °C 30 s, 72 °C 1 min; 25 cycles of 88 °C 30 s, 48 °C 30 s 72 °C 1 min; and 72 °C 10 min. Resultant products were sent to the Australian Genome Research Facility, Adelaide for Sanger sequencing.

#### The Extended Unidirectional Sequencing (EUS) method of *de novo* reference DNA barcoding

We aimed to exploit the massive excess in colony coverage on the Illumina MiSeq V3 platform to enable identification of sufficient long, high quality reads in both forward and reverse directions to allow merging and assembly of a full-length barcode.

A two-step amplification strategy using the above *rbcL* and *matK* primers (*matK* Gym-F1A + Gym-R1A for Gymnosperms^50^) was applied. For the initial step, each primer had an additional Illumina adapter^51^ at the 5′ end. Two pairs of primers were designed for each marker to sequence fragments in both directions on the MiSeq (i.e. P5-adapter + forward primer and P7-adapter + reverse primer; P7-adapter + forward primer and P5-adapter + reverse primer). The initial PCR used MyFi *taq* polymerase (Bioline) with 0.4 μM primer pair. Thermocycling conditions were: 95 °C 1 min; 30 cycles of 95 °C 15 s, 54 °C 15 s, 72 °C 15 s for *rbcL*. For *matK* thermocycling conditions were: 95 °C 1 min; 30 cycles of 95 °C 5 s, 52 °C 10 s, 72 °C 10 s. For *matK* Gymnosperm primers thermocycling conditions were: 95 °C 1 min; 8 cycles of 94 °C 15 s, 52 °C 15 s, 72 °C 15 s; 22 cycles of 88 °C 15 s, 48 °C 15 s, 72 °C 15 s; 72 °C 1 min. Products were then purified using Agencourt AMPure XP PCR Purification beads (Beckman Coulter) at a v/v ratio of 0.6x beads/PCR product.

The second PCR amplified the purified products with 0.4 μM of Nextera 96 Indices (Illumina) and MyFi polymerase (Bioline). Thermocycling conditions were: 95 °C 1 min; 5 cycles of 95 °C 5 s, 55 °C 10 s, 72 °C 10 s. A final purification was performed using the Agencourt AMPure XP PCR Purification beads at a v/v ratio of 0.6x beads/PCR product. Resultant products were quantified by qPCR using a RotorGene RG-6000 (Corbett) and the SYBR FAST qPCR Kit (Kapa Biosystems) with reference to known PhiX standards (Illumina). Libraries were pooled and a 20 pM aliquot was sequenced in a single direction on a MiSeq V3 Sequencer using a Version 3 kit (Illumina). The MiSeq software was configured for unidirectional sequencing by setting the run conditions: Read 1–609 cycles, Index Read 1–8 cycles; Index Read 2–8 cycles.

The MiSeq Bcl output files were demultiplexed and converted to fastq files using MiSeq Reporter. These files were imported into the code EUS Assembly for analysis (available at http://bit.ly/next-gen-barcode). In brief, EUS Assembly builds a consensus sequence base-by-base from the primer termini. Only those colonies that passed QF > 30 filter, contained <5% Ns in the most 5′ twenty bases and <1% Ns overall were used. Percent agreement for all colonies passing filter was recorded at each base position and the consensus base identity recorded. This process was actioned in the forward and reverse directions independently. The reverse sequences were then converted to reverse complementation and merged with the forward read. The raw data was then re-analysed summing base calls in both reads to provide consensus agreement at each position.

#### The Sonication-MicroAssembly (SMA) method of *de novo* DNA barcoding

The SMA strategy relies on sonication of amplicons followed by tag-labelling and sequencing. Post-hoc micro-assembly of the resultant sequences allowed us to derive longer length barcodes of *matK* for multiple samples in a single run. In the first instance this was performed using *matK* barcoding amplicons (xF + MALPR1, 790–915 bp). Next, near gene-length products were amplified for *rbcL* (>1.3 kb) and *matK* (>1.4 kb) from 36 Murraylands plant samples representing 21 species (Supplementary Table S5). Amplicons were generated using MyFi polymerase (Bioline) with 0.4 μM of each primer (Supplementary Table S6) using thermocycling conditions: 95 °C 1 min; 30 cycles of 95 °C 5 s, 54 °C (*rbcL*) or 50 °C (*matK*) 10 s, 72 °C 10 s; 72 °C 60 s. Samples were purified using the Agencourt AMPure XP PCR Purification beads. Purified samples were made up to 130 μl in nH_2_O and fragmented on a Covaris S2 machine in microTUBE AFA Fiber Pre-Slit Snap Cap 6 × 16 mm cuvettes (TrendBio) using the following: Intensity 3; Duty Cycle 5%; Cycles per Burst 200; Treatment Time 50 s; Temperature 7 °C. Sonicated samples were indexed for sequencing using the 96 NEB Next Illumina library preparation kit (New England Biolabs) as per the manufacturer’s instructions. Samples were analysed for fragment size and quantity using a High Sensitivity DNA Assay on a 2100 Bioanalyzer (Agilent) and a 20 nM aliquot sequenced by paired-end sequencing on a MiSeq V3 sequencer instrument with a Version 3 kit (Illumina) according to manufacturer’s instructions.

The MiSeq Bcl output files were demultiplexed and converted to fastq files using a MiSeq Reporter. These files were imported into a Python code SMA Assembly for analysis (available on http://bit.ly/next-gen-barcode). The code first passes the fastq data through a filter that replaces bases with QF < 30 with an N. The code then doubles the number of reads by adding the reverse complement of each filtered read. An initial assembled sequence is generated by selecting all reads that start with the forward primer and counting the number of appearances of each base in each base position in these reads. The initial assembled sequence is then extended to the complete barcode by an terative process. For each base position, the code searches for hitherto unmerged reads that match the most common bases in the 20 base positions upstream of the current position. Sequence from matching reads is then added to the previously assembled sequence. After all base positions are considered, the assembled sequence is trimmed to remove the primers. A Gnuplot script generates the graphical output based on the information collected during the barcode assembly process.

## Additional Information

**How to cite this article**: Wilkinson, M. J. *et al*. Replacing Sanger with Next Generation Sequencing to improve coverage and quality of reference DNA barcodes for plants. *Sci. Rep.* **7**, 46040; doi: 10.1038/srep46040 (2017).

**Publisher's note:** Springer Nature remains neutral with regard to jurisdictional claims in published maps and institutional affiliations.

## Supplementary Material

Supplementary Information
