# Supplementary material for: Replacing Sanger with Next Generation Sequencing to improve coverage and quality of reference DNA barcodes for plants
Source: Sci Rep. 2017 Apr 12;7:46040. doi: 10.1038/srep46040 (PMC5388885; doi:10.1038/srep46040)
Supplement: Supplementary Information [file srep46040-s1.pdf]

Supplementary Information

Replacing Sanger with Next Generation Sequencing to improve coverage and quality of reference DNA barcodes for plants

Comprises Supplementary Figures S1,S2, S3,S4; Supplementary Methods S1,S2,S3 & Supplementary Tables S1,S2,S3,S4,S5,S6

Mike J. Wilkinson[1*], Claudia Szabo[2], Caroline S. Ford[1], Yuval Yarom[2], Adam E. Croxford[3], Amanda Camp[4], Paul Gooding[5]

Supplementary Fig. S1. Information content of barcode alignments. Plots showing the relationship between the number of DNA barcodes compared in an alignment and the number of shared bases that can be used to distinguish between: a) *rbcL* barcodes; b) *matK* barcodes; c) only *rbcL* barcodes of length >300bases aligned and d) only *matK* barcodes of length >300 bases aligned.

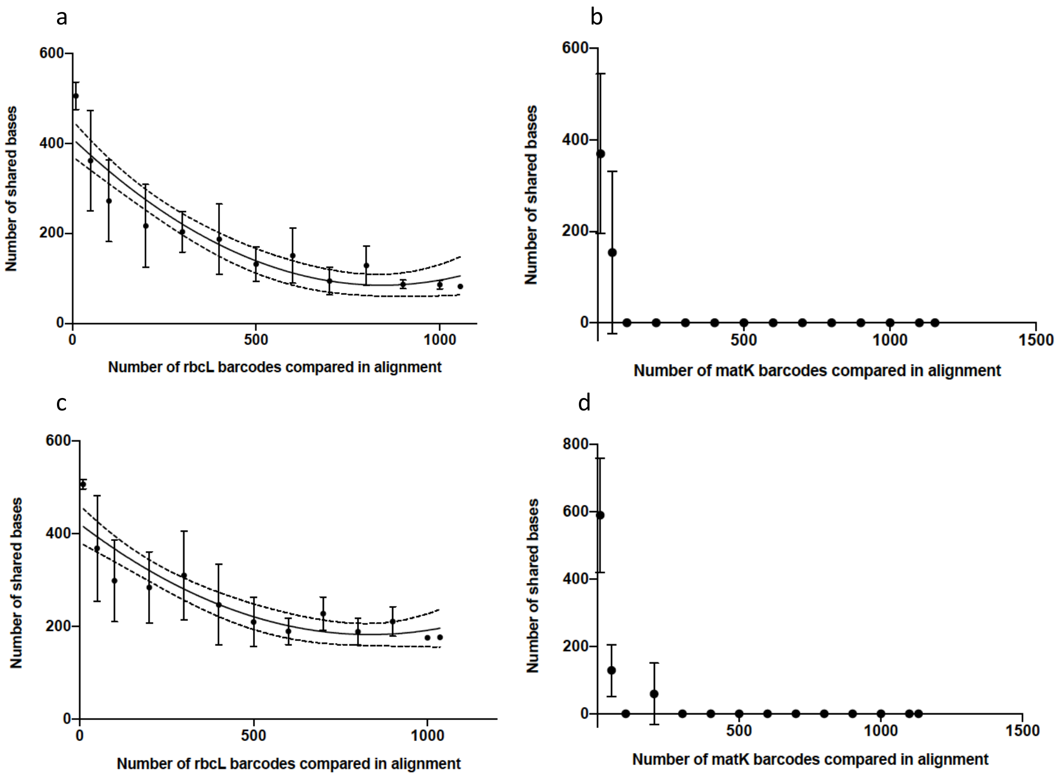

Supplementary Fig. S2. Genus identification accuracy for unknown samples matched to an incomplete reference DNA barcode resource. A model constructs a comprehensive reference barcode (CRB) resource from 721 whole chloroplast genomes and associated variants derived from the BOLD Systems 3 database to represent all barcodes in a hypothetical geographic area. The model then takes subsets of barcodes from the CRB to represent incomplete coverage of barcodes in the reference database (Interim Reference Barcode, IRB) and also a random set of unknown samples from the CRB to represent an Ecological Study Set. The panels below indicate the various outcomes: perceived identifications (red); ambiguous (blue); unknowns (black); true (pink) or false (orange) when identification of samples from the ESS (200 species) is attempted by exact matches made to IRBs of various sizes for: a) *rbcL*, b) *matK* and c) *rbcL* + *matK*.

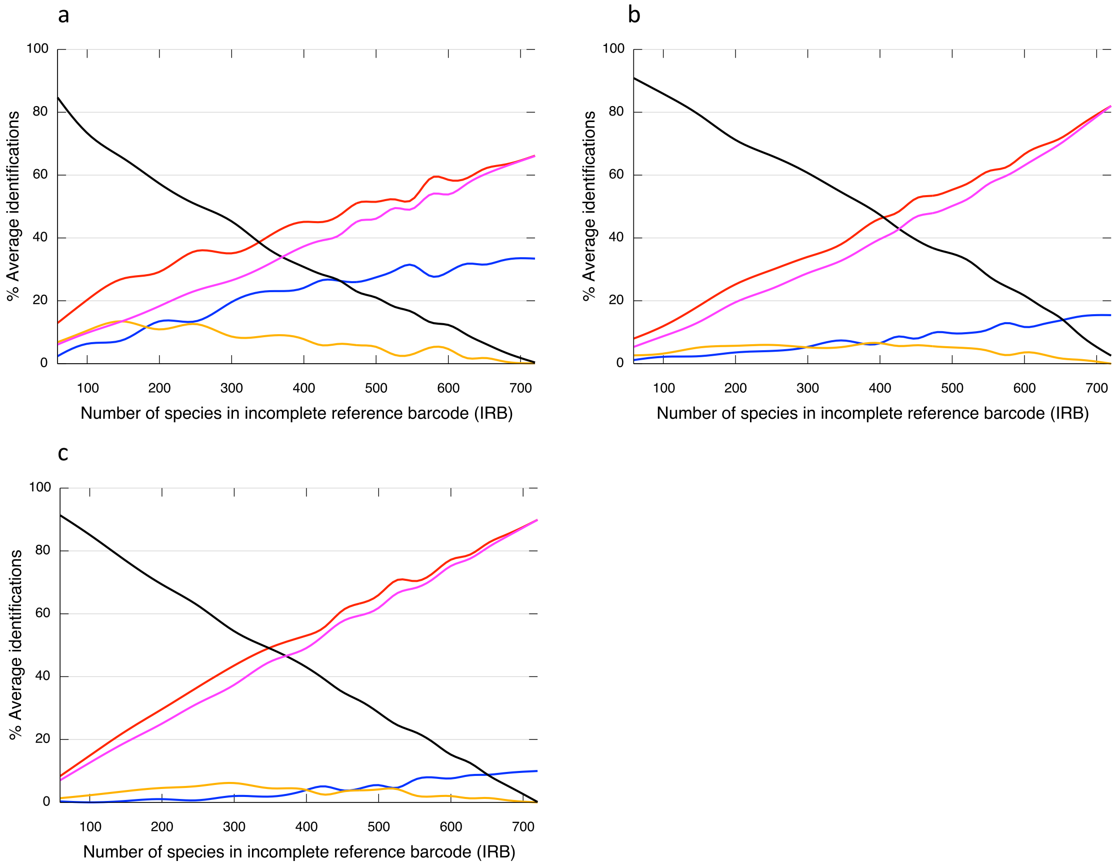

Supplementary Fig S3. Influence of Ecological Study Set (ESS) size on barcode diagnostic performance. A model constructs a comprehensive reference barcode (CRB) resource from 721 whole chloroplast genomes and associated variants derived from the BOLD Systems 3 database to represent all barcodes in a hypothetical region. The model then takes subsets of barcodes from the CRB to represent incomplete coverage of barcodes in the reference database (Interim Reference Barcode, IRB) and also a random set of unknown samples from the CRB to represent Ecological Study Sets of varying size. The panels below indicate the various outcomes: perceived identifications (red); ambiguous (blue); unknowns (black); true (pink); false (orange) when identification of samples taken from ESS sets of varying size (long dashed line = 50 species; square dashed line = 100 species; fine dashed line = 150 species; solid line = 200 species) is attempted by exact matches to IRBs of varying size (x-axis) for: a) *rbcL*, b) *matK* and c) *rbcL* + *matK*.

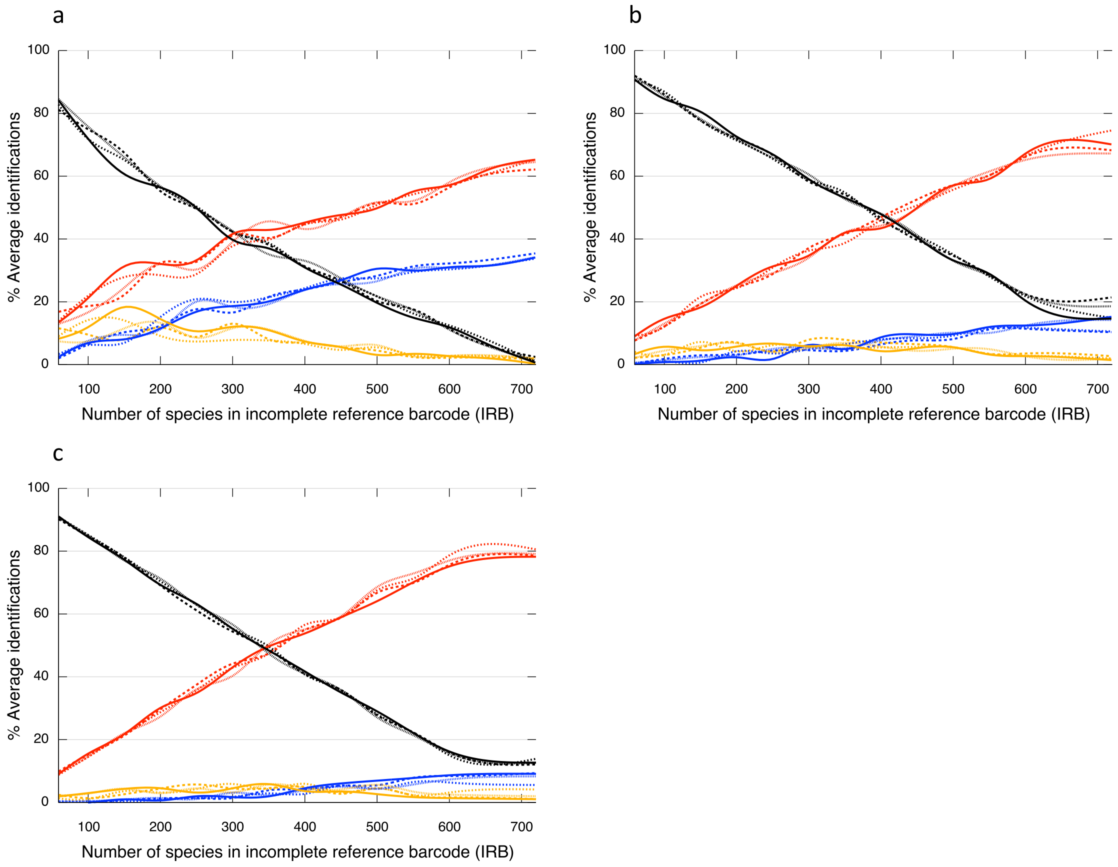

Supplementary Fig. S4. Extended reference DNA barcodes by Next Generation Sequencing. Extended amplicons (≈1,400bp) of *rbc*L and *mat*K were sonicated, tag-labelled and sequenced on the Illumina V3 MiSeq platform. Resultant fragments were assembled to create a contig of the entire amplicon. The panels below illustrate readouts indicating consensus sequences generated in the assembled contig (upper bar, where black=G; green=A; red=T and Blue=C); percent consensus agreement for all reads passing filter (red line); and the number of individual sequences contributing to each base position (blue line) for: *mat*K (a-d) and *rbcL* (e-h) with Amaranthaceae samples *Sclerolaena patenticuspis* M34 (a, e); *Maireana lobiflora* M45 (b, f); *Rhagodia crassifolia* M49 (c, g); *Rhagodia parabolica* M64 (d, h).

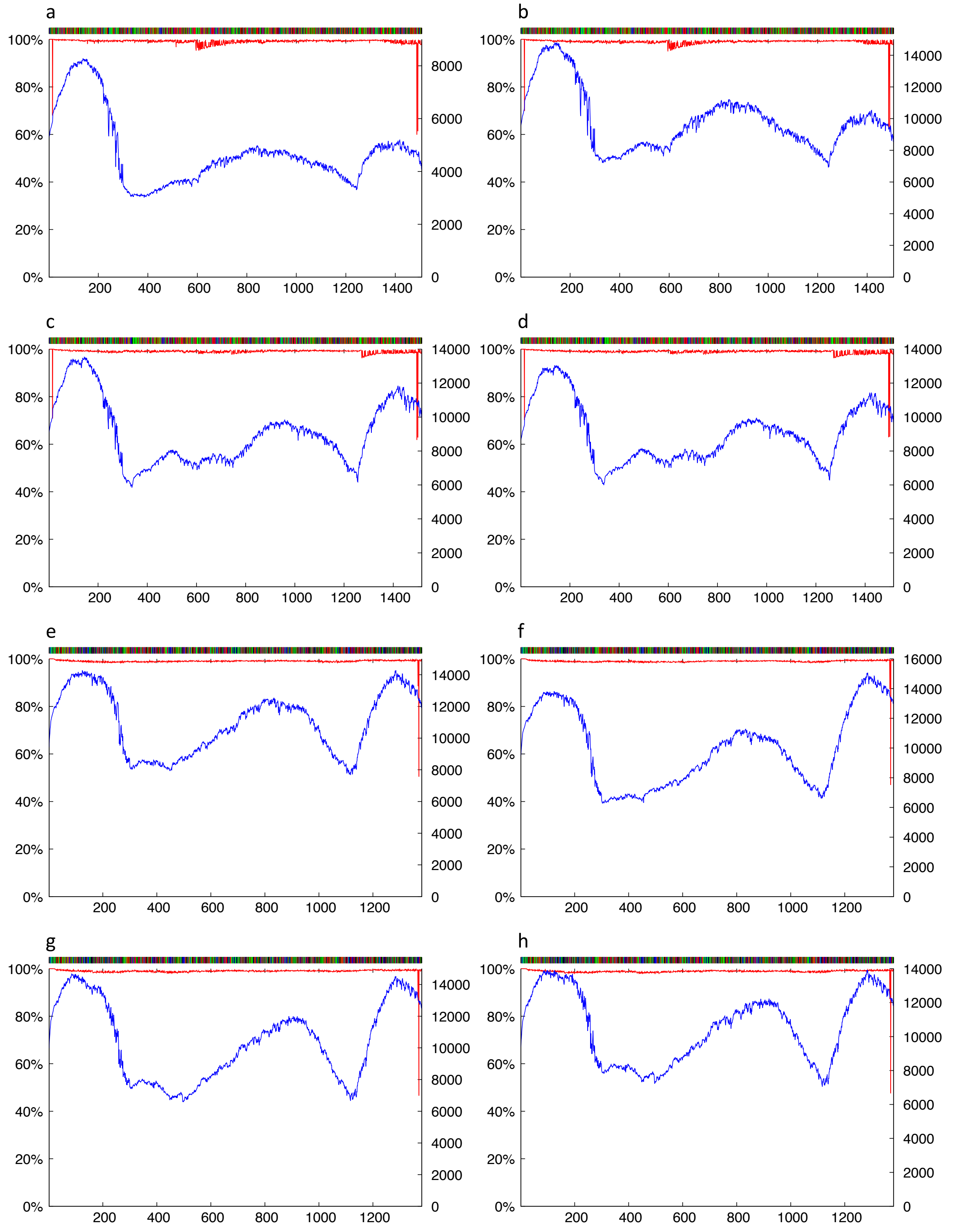

Supplementary Methods S1

We estimated the proportion of species in BOLD Systems (www.boldsystems.org, visited June 2016) containing both *rbcL* and *matK* by retrieving species records at frequencies reflecting the major taxonomic divisions of land plants. Entries at this time comprised 92.3% Magnoliophyta (331486/359087); 1.9% Pinophyta (6824/359087); 2.8%, Pteridophyta (10047/359087); 0.3% Lycopodiophyta (923/359087) and 2.7% Bryophyta (9807/359087). We first attained species lists for each of these phyla and, working sequentially down arbitrary pages, selected the appropriate number of species per phylum to a total of 1,000 (viz: 923 species of Magnoliophyta; 19 Pinophyta; 28 Pteridophyta; 3 Lycopodiophyta; 27 Bryophyta). To calculate the proportion of these species with 'full barcode coverage' (both *rbcL* and *matK*), single species searches were performed in BOLD and sequence presence recorded (Supplementary Table S1 online).

To test the effect of variable sequence overlap in multi-specimen alignments from BOLD Systems we downloaded all *rbcL* and *matK* entries for 101 congeneric species (Supplementary Table S2 online) into a Geneious 8.1 (Biomatters) database comprising 2,210 records (1056 *rbcL*, 1154 *matK*). For each locus sequences were aligned using MUSCLE (10 iterations). Left and right borders of universal coverage (bases present across all records) were identified and size (bp) of universally shared overlap recorded. We simulated partial representation of the reference database by randomly removing records and recalculating overlap. Five replicates per alignment size (number of records: 10; 50; 100; 200; 300; 400; 500; 600; 700; 800; 900; 1000, 1100) were generated using a random number generator (www.random.org). Non-linear regression of the resultant data was performed using GraphPad Prism V8.00 (La Jolla). To evaluate the effect of filtering short reads prior to alignment, we removed sequences <300bp (both loci) and <500bp (*rbcL*) or <600bp (*matK*) from the original data set and repeated the experiment.

Supplementary Methods S2

To model the importance of incomplete species coverage in the reference database, we first constructed an artificial Comprehensive Reference Barcode (CRB) database to represent the entire land plant flora of a hypothetical region. Here, we recovered full-gene sequences for *rbcL* and *matK* from 735 chloroplast genomes from the National Center for Biotechnology Information database (ncbi.nlm.nih.gov) of which 721 species contained both genes (Supplementary Table S3 online). These 721 *rbcL* and *matK* sequences were trimmed in Geneious 8.1 to match the nominal barcode regions internal to primers rbcLa_f[46] and rbcLa_rev[47] and *matK* xF[28] and MALPR1[40]. We simulated intraspecific variation by including all barcodes retrieved from BOLD Systems for these same species. In addition, we replaced ambiguous (N) and missing bases using the full-length sequences retrieved from the chloroplast genomes (Supplementary Table S3 online). Thus, all barcodes in our database were full-length.

To simulate the process of building a reference barcode database from scratch we created 200 separate Incomplete Reference Barcode (IRB) sets from the CRB, with each set containing the same number of species (N_s) in the range 70-721 species. This was achieved using the Python code IRB Assembly. IRB Assembly (available at http://bit.ly/next-gen-barcode) contains a 'Regional CRB dataset' representing the CRB. This dataset is encoded as a map, where the key is represented by the species/genus name, and the value is a barcode. If the key is not unique, i.e. a species (or genus) appears more than once in the CRB the value is then an array of barcodes. To generate IRBs from the Regional CRB dataset we first generate the IRB_Map, a dataset similar in structure to Regional_CRB. For each pre-determined IRB size *s*, a number of *s* keys are randomly selected from the Regional_CRB dataset, and are added to IRB_Map. CRBs and IRBs were assembled for *rbcL, matK* and the concatenated barcode *rbcL* + *matK* .

We entered these CRBs and IRBs into a model (IRB size) to characterize changes to the diagnostic properties of *rbcL* and *matK* as the IRB expands. The model (described below) identified barcodes as species-unique (true) when they possessed only one species name in both the IRB and CRB. However, if the barcode sequence was attached to two or more names in the IRB (also in the CRB), it was deemed ambiguous. Cases where discordance occurred between IRB and CRB assignments (species-unique barcodes in the IRB but not the CRB) were deemed false (negatives). The IRB Size model plotted the frequency of all diagnostic outcomes as the IRBs expanded from 70 to 721 species. This process was repeated with the species names replaced by the generic epithet alone to provide plots for genus-level diagnosis. The Python code IRB Size (available at http://bit.ly/next-gen-barcode) employs the Regional_CRB and IRB_Map, for species and genus respectively. The code goes through each key in IRB_Map and, if it has associated more than one value, that key is deemed ambiguous. If the key has associated one value in IRB_Map, but more than one value in Regional_CRB, then it is deemed false (negative). The code calculates the frequencies of these assignments as the number of keys in IRB_Map increases.

Supplementary Methods S3

Real-world identification using DNA barcodes requires comparison between unknown specimens and reference barcodes. To model this, we simulated variable collections of field specimens requiring identification: Ecological Study Sets (ESS). ESS assembly was achieved using the code ESS Diagnosis in which barcodes were randomly selected from the CRB with replacement and added to the ESS. We made no allowance for technical (sequencing, identification) error when generating either ESS or reference barcodes. For each ESS size *ess_s* (50, 100, 300, 700) our program (ESS Diagnosis) randomly selects *ess_s* barcodes from all the values in the Regional_CRB map and adds them to an array called ESS_Vector.

We next attempted to identify the species within each ESS by reference to barcodes in IRBs of differing size thus mimicking attempts to identify unknown specimens using incomplete reference barcode databases. The first iteration of the model used exact sequence matches as the only basis to define species identification. The model (described below) deemed that when ESS specimens possessed barcodes that uniquely matched barcode(s) with a single species name in the IRB these were perceived identifications. Specimens with barcodes shared by two or more IRB species were deemed ambiguous. Specimens that were assigned perceived from the IRB but were ambiguous in the CRB were categorized false. True identifications were barcodes were species-specific in both IRB and CRB. Failed matches were classified unknowns. The model used ESS sets of a single size (200 species) and one barcode (*rbcL*, *matK* or *rbcL+matK*) in each run, and recorded how each of the identification categories described above changed with IRB size. Our Python code ESS Diagnosis (available at http://bit.ly/next-gen-barcode) employs an additional data structure reverse_IRB_Map, where the keys are the unique IRB barcodes and the values are the species (or genus) names. Our code analyses each barcode *b* in ESS_Vector. For every barcode *b,* our code checks the reverse IRB_Map to see if the barcode maps to a unique species/genus name and classifies it as described above.

The model was repeated using the combined barcode (*rbcL+matK*) but imposing different sequence similarity thresholds *t* (97, 98, 99, 99.5 and 99.9% sequence similarity) to define sequence matches between the ESS query specimens (fixed at 200 species) and reference barcodes in the IRBs (50-721 species). The code ESS Diagnosis+ (available at http://bit.ly/next-gen-barcode) defined the optimum sequence similarity threshold (O_t) generating the highest proportion of true identifications out of all barcodes evaluated. Values of O_t were plotted as the size of the IRB increased towards 721 species. ESS Diagnosis+ employs an exact match between the barcode *b* in ESS_Vector and the keys in reverse_IRB_Map, and checks to see if there is a sequence similarity greater than a specified threshold *t*, with the values defined above. If the similarity exists, then the code proceeds as above.

Supplementary Table S1. **Core barcode coverage in BOLD Systems**. Coverage of the core barcode (*rbcL* and *matK*) among 1,000 species records in listed the BOLD Systems V3 database. Species and Phylum name are given in the first two columns, along with the presence (1) or absence (0) of *rbcL* sequence (column 3) and *matK* sequence (column 4).

Species	Phylum	rbcl	matK
Andreaea nitida	Bryophyta	1	0
Andreaea rupestris	Bryophyta	1	0
Anomobryum julaceum	Bryophyta	1	0
Aulacomnium androgynum	Bryophyta	1	0
Bartramia breviseta	Bryophyta	1	0
Bartramia halleriana	Bryophyta	1	0
Bartramia pomiformis	Bryophyta	1	0
Bartramia stricta	Bryophyta	1	0
Brachymenium nepalense	Bryophyta	1	0
Brachymenium pulchrum	Bryophyta	1	0
Bryum billardierei	Bryophyta	1	0
Bryum caespiticium	Bryophyta	1	0
Bryum pseudotriquetrum	Bryophyta	1	0
Imbribryum alpinum	Bryophyta	1	0
Leiomela bartramioides	Bryophyta	1	0
Mesochaete undulata	Bryophyta	1	0
Neosharpiella aztecorum	Bryophyta	1	0
Philonotis andina	Bryophyta	1	0
Philonotis fontana	Bryophyta	1	0
Philonotis nitida	Bryophyta	1	0
Plagiobryum cyclophyllum	Bryophyta	1	0
Plagiobryum zierii	Bryophyta	1	0
Plagiopus oederi	Bryophyta	1	0
Rhodobryum giganteum	Bryophyta	1	0
Rhodobryum ontariense	Bryophyta	1	0
Rosulabryum capillare	Bryophyta	1	0
Schistidium strictum	Bryophyta	1	0
Huperzia lucidula	Lycopodiophyta	1	1
Lycopodium clavatum	Lycopodiophyta	1	0
Lycopodium dendroideum	Lycopodiophyta	1	0
Hypoxis glabella	Magnoliophyta	1	0
Aa paleacea	Magnoliophyta	1	1
Acalypha hispida	Magnoliophyta	1	0
Acalypha hispida	Magnoliophyta	1	0
Acanthephippium mantinianum	Magnoliophyta	1	1
Acer palmatum	Magnoliophyta	1	1
Achlydosa glandulosa	Magnoliophyta	1	1
Acianthus exsertus	Magnoliophyta	1	1
Acineta chrysantha	Magnoliophyta	1	1
Acineta superba	Magnoliophyta	0	1
Acostaea costaricensis	Magnoliophyta	0	1
Ada aurantiaca	Magnoliophyta	0	1
Adenium obesum	Magnoliophyta	1	1
Aegiceras corniculatum	Magnoliophyta	1	1
Aerangis articulata	Magnoliophyta	1	1
Aeranthes grandiflora	Magnoliophyta	0	1
Aeranthes ramosa	Magnoliophyta	1	1
Aerides crispa	Magnoliophyta	0	1
Aerides emericii	Magnoliophyta	0	1
Aerides lawrenciae	Magnoliophyta	0	1
Aerides leeana	Magnoliophyta	0	1
Aerides multiflora	Magnoliophyta	1	1
Aerides ringens	Magnoliophyta	0	1
Aglaonema crispum	Magnoliophyta	1	1
Aglaonema crispum	Magnoliophyta	1	1
Agrostis mertensii	Magnoliophyta	1	1
Alamania punicea	Magnoliophyta	0	1
Aloe arborescens	Magnoliophyta	1	1
Aloe aristata	Magnoliophyta	1	1
Aloe barberae	Magnoliophyta	1	1
Aloe bowiea	Magnoliophyta	1	1
Aloe brevifolia	Magnoliophyta	1	1
Aloe buhrii	Magnoliophyta	1	1
Aloe cannellii	Magnoliophyta	1	1
Aloe commixta	Magnoliophyta	1	1
Aloe comosa	Magnoliophyta	1	1
Aloe dewinteri	Magnoliophyta	1	1
Aloe dichotoma	Magnoliophyta	1	1
Aloe dyeri	Magnoliophyta	1	1
Aloe erinacea	Magnoliophyta	1	1
Aloe fosteri	Magnoliophyta	1	1
Aloe glauca	Magnoliophyta	1	1
Aloe globuligemma	Magnoliophyta	1	1

Aloe gracilis	Magnoliophyta	1	1
Aloe greatheadii	Magnoliophyta	1	1
Aloe haworthioides	Magnoliophyta	1	1
Aloe hereroensis	Magnoliophyta	1	1
Aloe hexapetala	Magnoliophyta	1	1
Aloe humilis	Magnoliophyta	1	1
Aloe juddii	Magnoliophyta	1	1
Aloe kouebokkeveldensis	Magnoliophyta	1	1
Aloe lineata	Magnoliophyta	1	1
Aloe melanacantha	Magnoliophyta	1	1
Aloe microstigma	Magnoliophyta	1	1
Aloe perfoliata	Magnoliophyta	1	1
Aloe pictifolia	Magnoliophyta	1	1
Aloe plicatilis	Magnoliophyta	1	1
Aloe reynoldsii	Magnoliophyta	1	1
Aloe striata	Magnoliophyta	1	1
Aloe striatula	Magnoliophyta	1	1
Aloe succotrina	Magnoliophyta	1	1
Aloe tenuior	Magnoliophyta	1	1
Aloe thraskii	Magnoliophyta	1	1
Aloe variegata	Magnoliophyta	1	1
Alopecurus magellanicus	Magnoliophyta	1	1
Amblostoma armeniacum	Magnoliophyta	1	1
Amblostoma cernuum	Magnoliophyta	0	1
Amelichloa brachychaeta	Magnoliophyta	1	1
Amelichloa clandestina	Magnoliophyta	0	1
Amerorchis rotundifolia	Magnoliophyta	1	0
Ammophila breviligulata	Magnoliophyta	1	1
Amorphophallus yunnanensis	Magnoliophyta	1	1
Amorphophallus zenkeri	Magnoliophyta	1	1
Ampelodesmos mauritanicus	Magnoliophyta	1	1
Amphibromus fluitans	Magnoliophyta	1	0
Amphibromus scabrivalvis	Magnoliophyta	0	1
Amphipogon caricinus	Magnoliophyta	1	1
Amphipogon strictus	Magnoliophyta	1	0
Anatherostipa bomanii	Magnoliophyta	0	1
Anatherostipa hans-meyeri	Magnoliophyta	0	1
Anatherostipa mucronata	Magnoliophyta	0	1
Anatherostipa obtusa	Magnoliophyta	0	1
Anatherostipa rigidiseta	Magnoliophyta	0	1
Anatherostipa rosea	Magnoliophyta	0	1
Anatherostipa venusta	Magnoliophyta	0	1
Anchomanes difformis	Magnoliophyta	1	1
Ancistrochilus rothschildianus	Magnoliophyta	1	1
Andromeda polifolia	Magnoliophyta	1	1
Andropogon gerardii	Magnoliophyta	1	1
Androsace chamaejasme	Magnoliophyta	1	1
Androsace septentrionalis	Magnoliophyta	1	1
Anemone parviflora	Magnoliophyta	1	1
Angraecum magdalenae	Magnoliophyta	0	1
Angraecum sesquipedale	Magnoliophyta	1	1
Anguloa hohenlohii	Magnoliophyta	0	1
Ansellia africana	Magnoliophyta	1	1
Antennaria friesiana	Magnoliophyta	1	1
Antennaria media	Magnoliophyta	1	1
Anthogonium gracile	Magnoliophyta	1	1
Anthoxanthum arcticum	Magnoliophyta	1	1
Anthoxanthum monticola	Magnoliophyta	1	1
Anthoxanthum monticola	Magnoliophyta	1	1
Anthurium scherzerianum	Magnoliophyta	1	0
Aphyllorchis caudata	Magnoliophyta	1	0
Aphyllorchis montana	Magnoliophyta	1	0
Aplectrum hyemale	Magnoliophyta	1	1
Arbutus unedo	Magnoliophyta	1	1
Arctagrostis latifolia	Magnoliophyta	1	1
Arctophila fulva	Magnoliophyta	1	1
Arctous rubra	Magnoliophyta	1	1
Areca catechu	Magnoliophyta	1	1
Areca triandra	Magnoliophyta	1	1
Arenaria longipedunculata	Magnoliophyta	1	1
Arenga australasica	Magnoliophyta	1	1
Arenga brevipes	Magnoliophyta	1	1
Arenga caudata	Magnoliophyta	1	1
Arenga engleri	Magnoliophyta	1	1
Arenga hastata	Magnoliophyta	1	1
Arenga hookeriana	Magnoliophyta	1	1
Arenga listeri	Magnoliophyta	1	1
Arenga longicarpa	Magnoliophyta	1	0
Arenga microcarpa	Magnoliophyta	1	1
Arenga obtusifolia	Magnoliophyta	1	1
Arenga pinnata	Magnoliophyta	1	1

Arenga porphyrocarpa	Magnoliophyta	1	1
Arenga ryukyuensis	Magnoliophyta	1	1
Arenga tremula	Magnoliophyta	1	1
Arenga undulatifolia	Magnoliophyta	1	1
Arenga westerhoutii	Magnoliophyta	1	1
Ariopsis peltata	Magnoliophyta	1	1
Arisaema serratum	Magnoliophyta	1	1
Arisaema speciosum	Magnoliophyta	1	1
Arisaema tortuosum	Magnoliophyta	1	1
Arisaema triphyllum	Magnoliophyta	1	1
Arisarum vulgare	Magnoliophyta	1	1
Arnica frigida	Magnoliophyta	1	1
Artemisia borealis	Magnoliophyta	1	1
Arum alpinum	Magnoliophyta	1	1
Arum apulum	Magnoliophyta	1	1
Arum balansanum	Magnoliophyta	1	1
Arum byzantinum	Magnoliophyta	1	1
Arum concinnatum	Magnoliophyta	1	1
Arum creticum	Magnoliophyta	1	1
Arum cylindraceum	Magnoliophyta	1	1
Arum cyrenaicum	Magnoliophyta	1	1
Arum dioscoridis	Magnoliophyta	1	1
Arum elongatum	Magnoliophyta	1	1
Arum euxinum	Magnoliophyta	1	1
Arum gratum	Magnoliophyta	1	1
Arum hygrophilum	Magnoliophyta	1	1
Arum idaeum	Magnoliophyta	1	1
Arum italicum	Magnoliophyta	1	1
Arum jacquemontii	Magnoliophyta	1	1
Arum korolkowii	Magnoliophyta	1	1
Arum maculatum	Magnoliophyta	1	1
Arum nigrum	Magnoliophyta	1	1
Arum orientale	Magnoliophyta	1	1
Arum palaestinum	Magnoliophyta	1	1
Arum pictum	Magnoliophyta	1	1
Arum purpureospathum	Magnoliophyta	1	1
Arum rupicola	Magnoliophyta	1	1
Arum sintenisii	Magnoliophyta	1	1
Asparagus aethiopicus	Magnoliophyta	1	1
Asparagus aethiopicus	Magnoliophyta	1	1
Asterogyne martiana	Magnoliophyta	1	1
Astragalus alpinus	Magnoliophyta	1	1
Astragalus alpinus	Magnoliophyta	1	1
Astragalus eucosmus	Magnoliophyta	1	1
Astragalus eucosmus	Magnoliophyta	1	1
Astragalus nutzotinensis	Magnoliophyta	1	1
Astragalus richardsonii	Magnoliophyta	1	1
Astragalus umbellatus	Magnoliophyta	1	1
Astrocaryum alatum	Magnoliophyta	1	1
Astrocaryum chambira	Magnoliophyta	0	1
Astrocaryum gynacanthum	Magnoliophyta	0	1
Astrocaryum huaimi	Magnoliophyta	0	1
Astrocaryum jauari	Magnoliophyta	0	1
Astrocaryum malybo	Magnoliophyta	0	1
Astrocaryum mexicanum	Magnoliophyta	0	1
Astrocaryum minus	Magnoliophyta	0	1
Astrocaryum murumuru	Magnoliophyta	1	1
Astrocaryum paramaca	Magnoliophyta	0	1
Astrocaryum rodriguesii	Magnoliophyta	1	1
Astrocaryum sciophilum	Magnoliophyta	1	1
Astrocaryum scopatum	Magnoliophyta	1	1
Astrocaryum standleyanum	Magnoliophyta	0	1
Astrocaryum urostachys	Magnoliophyta	0	1
Astrocaryum vulgare	Magnoliophyta	0	1
Astroloba bullulata	Magnoliophyta	1	1
Astroloba corrugata	Magnoliophyta	1	1
Astroloba foliolosa	Magnoliophyta	1	1
Astroloba herrei	Magnoliophyta	1	1
Astroloba rubriflora	Magnoliophyta	1	1
Astroloba smutsiana	Magnoliophyta	1	1
Astroloba spiralis	Magnoliophyta	1	1
Attalea allenii	Magnoliophyta	1	1
Attalea butyracea	Magnoliophyta	1	0
Attalea phalerata	Magnoliophyta	0	1
Austrostipa acrociliata	Magnoliophyta	1	1
Austrostipa aphylla	Magnoliophyta	1	1
Austrostipa aquarii	Magnoliophyta	1	1
Austrostipa aristiglumis	Magnoliophyta	1	1
Austrostipa bigeniculata	Magnoliophyta	1	0
Austrostipa blackii	Magnoliophyta	1	1
Austrostipa breviglumis	Magnoliophyta	1	1

Austrostipa centralis	Magnoliophyta	0	1
Austrostipa compressa	Magnoliophyta	1	1
Austrostipa crinita	Magnoliophyta	1	1
Austrostipa curticoma	Magnoliophyta	1	0
Austrostipa densiflora	Magnoliophyta	1	1
Austrostipa dongicola	Magnoliophyta	1	0
Austrostipa drummondii	Magnoliophyta	1	1
Austrostipa elegantissima	Magnoliophyta	1	1
Austrostipa eremophila	Magnoliophyta	1	0
Austrostipa exilis	Magnoliophyta	1	1
Austrostipa feresetacea	Magnoliophyta	1	1
Austrostipa flavescens	Magnoliophyta	1	1
Austrostipa geoffrey	Magnoliophyta	1	1
Austrostipa gibbosa	Magnoliophyta	1	1
Austrostipa hemipogon	Magnoliophyta	1	1
Austrostipa juncifolia	Magnoliophyta	1	1
Austrostipa macalpinei	Magnoliophyta	1	1
Austrostipa mollis	Magnoliophyta	1	1
Austrostipa muelleri	Magnoliophyta	1	1
Austrostipa mundula	Magnoliophyta	1	1
Austrostipa nitida	Magnoliophyta	1	1
Austrostipa nivicola	Magnoliophyta	1	1
Austrostipa nodosa	Magnoliophyta	1	1
Austrostipa nullanulla	Magnoliophyta	1	1
Austrostipa oligostachya	Magnoliophyta	1	1
Austrostipa platychaeta	Magnoliophyta	1	1
Austrostipa puberula	Magnoliophyta	1	1
Austrostipa pubescens	Magnoliophyta	1	0
Austrostipa pubinodis	Magnoliophyta	1	1
Austrostipa pubinodis	Magnoliophyta	1	1
Austrostipa ramosissima	Magnoliophyta	1	0
Austrostipa rudis	Magnoliophyta	1	1
Austrostipa scabra	Magnoliophyta	1	1
Austrostipa semibarbata	Magnoliophyta	1	1
Austrostipa setacea	Magnoliophyta	1	0
Austrostipa stuposa	Magnoliophyta	1	1
Austrostipa tenuifolia	Magnoliophyta	1	1
Austrostipa trichophylla	Magnoliophyta	1	1
Austrostipa tuckeri	Magnoliophyta	1	1
Austrostipa variabilis	Magnoliophyta	1	0
Austrostipa verticillata	Magnoliophyta	1	1
Austrostipa wakoolica	Magnoliophyta	1	0
Avicennia marina	Magnoliophyta	1	1
Bactris acanthocarpa	Magnoliophyta	0	1
Bactris bifida	Magnoliophyta	0	1
Bactris campestris	Magnoliophyta	0	1
Bactris constanciae	Magnoliophyta	0	1
Bactris ferruginea	Magnoliophyta	0	1
Bactris gasipaes	Magnoliophyta	1	1
Bactris humilis	Magnoliophyta	1	0
Bactris killipii	Magnoliophyta	0	1
Bactris major	Magnoliophyta	0	1
Bactris maraja	Magnoliophyta	0	1
Bactris pliniana	Magnoliophyta	0	1
Bactris riparia	Magnoliophyta	0	1
Bactris setulosa	Magnoliophyta	0	1
Bactris simplicifrons	Magnoliophyta	0	1
Balaka seemannii	Magnoliophyta	1	1
Bambusa vulgaris	Magnoliophyta	1	1
Bambusa vulgaris	Magnoliophyta	1	1
Barcella odora	Magnoliophyta	1	1
Basselinia velutina	Magnoliophyta	1	1
Bauhinia variegata	Magnoliophyta	1	1
Bauhinia variegata	Magnoliophyta	1	1
Beaucarnea gracilis	Magnoliophyta	1	1
Beccariophoenix madagascariensis	Magnoliophyta	1	1
Bentinckia nicobarica	Magnoliophyta	1	1
Betula glandulosa	Magnoliophyta	1	1
Biarum arundanum	Magnoliophyta	1	1
Biarum davisii	Magnoliophyta	1	1
Biarum dispar	Magnoliophyta	1	1
Biarum tenuifolium	Magnoliophyta	1	1
Bismarckia nobilis	Magnoliophyta	1	1
Bistorta vivipara	Magnoliophyta	1	1
Borassodendron borneense	Magnoliophyta	1	0
Borassodendron machadonis	Magnoliophyta	1	1
Borassus aethiopum	Magnoliophyta	1	1
Borassus akeassii	Magnoliophyta	1	0
Borassus flabellifer	Magnoliophyta	1	1
Borassus heineanus	Magnoliophyta	1	0
Borassus madagascariensis	Magnoliophyta	1	0

Brahea dulcis	Magnoliophyta	1	1
Brassica insularis	Magnoliophyta	1	1
Brassica villosa	Magnoliophyta	1	1
Brassiophoenix schumannii	Magnoliophyta	1	1
Braya glabella	Magnoliophyta	1	1
Braya humilis	Magnoliophyta	1	1
Bromus pumpellianus	Magnoliophyta	1	1
Brongniartikentia lanuginosa	Magnoliophyta	1	1
Bruguiera cylindrica	Magnoliophyta	1	1
Burretiokentia hapala	Magnoliophyta	1	1
Butia capitata	Magnoliophyta	1	1
Butia eriospatha	Magnoliophyta	1	0
Caladium bicolor	Magnoliophyta	1	1
Calamagrostis canadensis	Magnoliophyta	1	1
Calamagrostis deschampsioides	Magnoliophyta	1	1
Calamagrostis lapponica	Magnoliophyta	1	1
Calamagrostis lapponica	Magnoliophyta	1	1
Calamagrostis purpurascens	Magnoliophyta	1	1
Calamagrostis stricta	Magnoliophyta	1	1
Calamus aruensis	Magnoliophyta	0	1
Calamus caesius	Magnoliophyta	1	0
Calamus hollrungii	Magnoliophyta	1	0
Calla palustris	Magnoliophyta	1	1
Calliandra tergemina	Magnoliophyta	1	1
Calyptrocalyx albertisianus	Magnoliophyta	1	1
Calyptrocalyx stenocrista	Magnoliophyta	1	0
Calyptrogyne ghiesbreghtiana	Magnoliophyta	1	1
Calyptronoma occidentalis	Magnoliophyta	1	1
Calyptronoma rivalis	Magnoliophyta	1	1
Campecarpus fulcitus	Magnoliophyta	1	1
Canna glauca	Magnoliophyta	1	0
Canna glauca	Magnoliophyta	1	0
Caralluma europaea	Magnoliophyta	1	1
Cardamine digitata	Magnoliophyta	1	1
Cardamine pratensis	Magnoliophyta	1	1
Carex aquatilis	Magnoliophyta	1	1
Carex atrofusca	Magnoliophyta	1	1
Carex bicolor	Magnoliophyta	1	1
Carex bigelowii	Magnoliophyta	1	1
Carex caroliniana	Magnoliophyta	0	1
Carex chordorrhiza	Magnoliophyta	1	1
Carex concinna	Magnoliophyta	1	1
Carex fuliginosa	Magnoliophyta	1	1
Carex glacialis	Magnoliophyta	1	1
Carex glareosa	Magnoliophyta	1	1
Carex gracillima	Magnoliophyta	1	1
Carex granularis	Magnoliophyta	1	1
Carex gravida	Magnoliophyta	0	1
Carex grayi	Magnoliophyta	0	1
Carex grisea	Magnoliophyta	0	1
Carex gynandra	Magnoliophyta	1	1
Carex gynocrates	Magnoliophyta	1	1
Carex gynodynama	Magnoliophyta	0	1
Carex halleriana	Magnoliophyta	1	1
Carex hallii	Magnoliophyta	0	1
Carex krausei	Magnoliophyta	1	1
Carex lachenalii	Magnoliophyta	1	1
Carex marina	Magnoliophyta	1	1
Carex maritima	Magnoliophyta	1	1
Carex membranacea	Magnoliophyta	1	1
Carex nardina	Magnoliophyta	1	1
Carex norvegica	Magnoliophyta	1	1
Carex petricosa	Magnoliophyta	1	1
Carex rariflora	Magnoliophyta	1	1
Carex rotundata	Magnoliophyta	1	1
Carex rupestris	Magnoliophyta	1	1
Carex scirpoidea	Magnoliophyta	1	1
Carex subspathacea	Magnoliophyta	1	1
Carex supina	Magnoliophyta	1	1
Carex ursina	Magnoliophyta	1	1
Carex vaginata	Magnoliophyta	1	1
Carpentaria acuminata	Magnoliophyta	1	1
Carpoxylon macrospermum	Magnoliophyta	1	1
Caryota kiriwongensis	Magnoliophyta	1	1
Caryota maxima	Magnoliophyta	1	1
Caryota mitis	Magnoliophyta	1	1
Caryota monostachya	Magnoliophyta	1	1
Caryota obtusa	Magnoliophyta	1	1
Caryota ophiopellis	Magnoliophyta	1	1
Caryota rumphiana	Magnoliophyta	1	1
Caryota urens	Magnoliophyta	1	1

Caryota urens	Magnoliophyta	1	1
Caryota urens	Magnoliophyta	1	1
Cassiope tetragona	Magnoliophyta	1	1
Castilleja elegans	Magnoliophyta	1	1
Cerastium arcticum	Magnoliophyta	1	1
Cerastium beeringianum	Magnoliophyta	1	1
Ceriops tagal	Magnoliophyta	1	1
Ceroxylon alpinum	Magnoliophyta	1	1
Ceroxylon amazonicum	Magnoliophyta	0	1
Ceroxylon echinulatum	Magnoliophyta	1	1
Ceroxylon parvifrons	Magnoliophyta	0	1
Ceroxylon parvum	Magnoliophyta	0	1
Ceroxylon quindiuense	Magnoliophyta	1	1
Ceroxylon ventricosum	Magnoliophyta	0	1
Ceroxylon vogelianum	Magnoliophyta	0	1
Cestrum nocturnum	Magnoliophyta	1	1
Cestrum nocturnum	Magnoliophyta	1	1
Chamaedorea adscendens	Magnoliophyta	0	1
Chamaedorea allenii	Magnoliophyta	0	1
Chamaedorea alternans	Magnoliophyta	0	1
Chamaedorea brachypoda	Magnoliophyta	0	1
Chamaedorea costaricana	Magnoliophyta	0	1
Chamaedorea crucensis	Magnoliophyta	0	1
Chamaedorea dammeriana	Magnoliophyta	0	1
Chamaedorea elatior	Magnoliophyta	0	1
Chamaedorea ernesti-augustii	Magnoliophyta	0	1
Chamaedorea fragrans	Magnoliophyta	0	1
Chamaedorea geonomiformis	Magnoliophyta	0	1
Chamaedorea glaucifolia	Magnoliophyta	0	1
Chamaedorea hooperiana	Magnoliophyta	0	1
Chamaedorea klotzschiana	Magnoliophyta	0	1
Chamaedorea linearis	Magnoliophyta	0	1
Chamaedorea metallica	Magnoliophyta	0	1
Chamaedorea microspadix	Magnoliophyta	1	1
Chamaedorea nationsiana	Magnoliophyta	0	1
Chamaedorea neurochlamys	Magnoliophyta	0	1
Chamaedorea oblongata	Magnoliophyta	0	1
Chamaedorea pedunculata	Magnoliophyta	0	1
Chamaedorea pinnatifrons	Magnoliophyta	0	1
Chamaedorea plumosa	Magnoliophyta	0	1
Chamaedorea pochutlensis	Magnoliophyta	0	1
Chamaedorea schiedeana	Magnoliophyta	0	1
Chamaedorea seifrizii	Magnoliophyta	1	1
Chamaedorea stolonifera	Magnoliophyta	0	1
Chamaedorea tenella	Magnoliophyta	0	1
Chamaedorea tepejilote	Magnoliophyta	0	1
Chamaerops humilis	Magnoliophyta	1	1
Chambeyronia macrocarpa	Magnoliophyta	1	1
Chamerion latifolium	Magnoliophyta	1	1
Chelyocarpus repens	Magnoliophyta	1	0
Chelyocarpus ulei	Magnoliophyta	1	1
Chortolirion angolense	Magnoliophyta	1	1
Chuniophoenix hainanensis	Magnoliophyta	1	0
Chuniophoenix nana	Magnoliophyta	1	1
Clinosperma bracteale	Magnoliophyta	1	1
Clinostigma savoryanum	Magnoliophyta	1	1
Coccothrinax argentata	Magnoliophyta	1	1
Cochlearia groenlandica	Magnoliophyta	1	1
Codiaeum variegatum	Magnoliophyta	1	1
Colocasia antiquorum	Magnoliophyta	1	1
Colocasia esculenta	Magnoliophyta	1	1
Colocasia heterochroma	Magnoliophyta	1	1
Colocasia lihengiae	Magnoliophyta	1	1
Colocasia yunnanensis	Magnoliophyta	1	1
Colpothrinax wrightii	Magnoliophyta	1	1
Copernicia prunifera	Magnoliophyta	1	1
Cordyline fruticosa	Magnoliophyta	1	1
Cordyline fruticosa	Magnoliophyta	1	1
Corypha taliera	Magnoliophyta	1	1
Corypha umbraculifera	Magnoliophyta	1	1
Corypha utan	Magnoliophyta	1	0
Couroupita guianensis	Magnoliophyta	1	1
Cryosophila warscewiczii	Magnoliophyta	0	1
Cryptocoryne sinensis	Magnoliophyta	1	0
Cyclopia genistoides	Magnoliophyta	1	1
Cyphokentia macrostachya	Magnoliophyta	1	1
Cyphophoenix nucele	Magnoliophyta	1	1
Cyphosperma balansae	Magnoliophyta	1	1
Cyrtostachys renda	Magnoliophyta	1	1
Dendrobium loddigesii	Magnoliophyta	1	1
Dendrobium macraei	Magnoliophyta	1	1

Dendrobium macrostachyum	Magnoliophyta	1	1
Dendrobium microbulbon	Magnoliophyta	1	1
Dendrobium miyakei	Magnoliophyta	1	1
Dendrobium moniliforme	Magnoliophyta	1	1
Dendrobium moschatum	Magnoliophyta	1	1
Dendrobium nobile	Magnoliophyta	1	1
Dendrobium nodosum	Magnoliophyta	1	1
Dendrobium nutantiflorum	Magnoliophyta	1	1
Dendrobium ochreatum	Magnoliophyta	1	1
Dendrobium officinale	Magnoliophyta	1	1
Dendrobium ovatum	Magnoliophyta	1	1
Deschampsia brevifolia	Magnoliophyta	1	1
Deschampsia cespitosa	Magnoliophyta	1	1
Deschampsia sukatschewii	Magnoliophyta	1	1
Desmoncus mitis	Magnoliophyta	0	1
Desmoncus orthacanthos	Magnoliophyta	1	1
Desmoncus polyacanthos	Magnoliophyta	0	1
Dianthus busambrae	Magnoliophyta	1	0
Dianthus rupicola	Magnoliophyta	1	1
Dictyocaryum lamarckianum	Magnoliophyta	1	1
Dictyosperma album	Magnoliophyta	1	1
Diospyros vestita	Magnoliophyta	1	1
Diospyros vieillardii	Magnoliophyta	1	1
Diospyros wallichii	Magnoliophyta	1	1
Diospyros yaouhensis	Magnoliophyta	1	1
Doryanthes palmeri	Magnoliophyta	1	1
Draba cinerea	Magnoliophyta	1	1
Draba corymbosa	Magnoliophyta	1	1
Draba glabella	Magnoliophyta	1	1
Draba nivalis	Magnoliophyta	1	1
Draba oblongata	Magnoliophyta	1	1
Draba pilosa	Magnoliophyta	1	1
Dracaena fragrans	Magnoliophyta	1	0
Dracaena fragrans	Magnoliophyta	1	0
Dracunculus canariensis	Magnoliophyta	1	1
Dracunculus vulgaris	Magnoliophyta	1	1
Dransfieldia micrantha	Magnoliophyta	1	1
Dryas integrifolia	Magnoliophyta	1	1
Drymophloeus litigiosus	Magnoliophyta	1	1
Dupontia fisheri	Magnoliophyta	1	1
Duranta erecta	Magnoliophyta	1	1
Duranta erecta	Magnoliophyta	1	1
Dypsis lastelliana	Magnoliophyta	1	0
Dypsis lutescens	Magnoliophyta	1	1
Echinochloa frumentacea	Magnoliophyta	1	1
Elaeis oleifera	Magnoliophyta	1	1
Eleocharis acicularis	Magnoliophyta	1	1
Eleusine coracana	Magnoliophyta	1	1
Elymus alaskanus	Magnoliophyta	1	1
Elymus macrourus	Magnoliophyta	1	1
Elymus violaceus	Magnoliophyta	1	1
Eminium spiculatum	Magnoliophyta	1	1
Empetrum nigrum	Magnoliophyta	1	1
Empodium veratrifolium	Magnoliophyta	1	1
Epilobium arcticum	Magnoliophyta	1	1
Epipremnum aureum	Magnoliophyta	1	1
Epipremnum aureum	Magnoliophyta	1	1
Eremospatha wendlandiana	Magnoliophyta	1	1
Erigeron compositus	Magnoliophyta	1	1
Eriophorum angustifolium	Magnoliophyta	1	1
Eriophorum brachyantherum	Magnoliophyta	1	1
Eriophorum callitrix	Magnoliophyta	1	1
Eriophorum chamissonis	Magnoliophyta	1	1
Eriophorum scheuchzeri	Magnoliophyta	1	1
Eriophorum triste	Magnoliophyta	1	1
Eriophorum vaginatum	Magnoliophyta	1	1
Erysimum pallasii	Magnoliophyta	1	1
Eugeissona tristis	Magnoliophyta	1	1
Euterpe oleracea	Magnoliophyta	1	1
Eutrema edwardsii	Magnoliophyta	1	1
Festuca altaica	Magnoliophyta	1	1
Festuca baffinensis	Magnoliophyta	1	1
Festuca brachyphylla	Magnoliophyta	1	1
Festuca brevissima	Magnoliophyta	1	1
Festuca edlundiae	Magnoliophyta	1	1
Festuca hyperborea	Magnoliophyta	1	1
Festuca lenensis	Magnoliophyta	1	1
Festuca rubra	Magnoliophyta	1	1
Festuca viviparoidea	Magnoliophyta	1	1
Ficus benjamina	Magnoliophyta	1	1
Ficus benjamina	Magnoliophyta	1	1

Filarum manserichense	Magnoliophyta	1	1
Gardenia jasminoides	Magnoliophyta	1	1
Gastrococos crispa	Magnoliophyta	1	1
Gaussia attenuata	Magnoliophyta	1	1
Gaussia gomez-pompae	Magnoliophyta	0	1
Gaussia maya	Magnoliophyta	1	1
Gaussia spirituana	Magnoliophyta	0	1
Genista demarcoi	Magnoliophyta	1	1
Genista madoniensis	Magnoliophyta	1	0
Geonoma congesta	Magnoliophyta	1	1
Geonoma oxycarpa	Magnoliophyta	1	0
Gladiolus marlothii	Magnoliophyta	0	1
Gladiolus martleyi	Magnoliophyta	0	1
Gladiolus meliusculus	Magnoliophyta	0	1
Gladiolus meridionalis	Magnoliophyta	0	1
Gladiolus microcarpus	Magnoliophyta	0	1
Glandularia pulchella	Magnoliophyta	1	0
Glandularia pulchella	Magnoliophyta	1	0
Gonatopus angustus	Magnoliophyta	1	1
Grevillea robusta	Magnoliophyta	1	1
Grevillea robusta	Magnoliophyta	1	1
Guihaia argyrata	Magnoliophyta	1	1
Gymnostachys anceps	Magnoliophyta	1	1
Halerpestes cymbalaria	Magnoliophyta	1	1
Hamelia patens	Magnoliophyta	1	1
Haworthia angustifolia	Magnoliophyta	1	1
Haworthia arachnoidea	Magnoliophyta	1	1
Haworthia arachnoidea	Magnoliophyta	1	1
Haworthia aristata	Magnoliophyta	1	1
Haworthia attenuata	Magnoliophyta	1	1
Haworthia bayeri	Magnoliophyta	1	1
Haworthia blackburniae	Magnoliophyta	1	1
Haworthia bolusii	Magnoliophyta	1	1
Haworthia bruynsii	Magnoliophyta	1	1
Haworthia chloracantha	Magnoliophyta	1	1
Haworthia coarctata	Magnoliophyta	1	1
Haworthia cooperi	Magnoliophyta	1	1
Haworthia cymbiformis	Magnoliophyta	1	1
Haworthia decipiens	Magnoliophyta	1	1
Haworthia emelyae	Magnoliophyta	1	1
Haworthia fasciata	Magnoliophyta	1	1
Haworthia floribunda	Magnoliophyta	1	1
Haworthia glauca	Magnoliophyta	1	1
Haworthia gracilis	Magnoliophyta	1	1
Haworthia herbacea	Magnoliophyta	1	1
Haworthia kingiana	Magnoliophyta	1	1
Haworthia koelmaniorum	Magnoliophyta	1	1
Haworthia limifolia	Magnoliophyta	1	1
Haworthia limifolia	Magnoliophyta	1	1
Haworthia lockwoodii	Magnoliophyta	1	1
Haworthia longiana	Magnoliophyta	1	1
Haworthia maculata	Magnoliophyta	1	1
Haworthia magnifica	Magnoliophyta	1	1
Haworthia marumiana	Magnoliophyta	1	1
Haworthia marxii	Magnoliophyta	1	1
Haworthia minor	Magnoliophyta	1	1
Haworthia mirabilis	Magnoliophyta	1	1
Haworthia mirabilis	Magnoliophyta	0	1
Haworthia monticola	Magnoliophyta	1	1
Haworthia mucronata	Magnoliophyta	1	1
Haworthia mutica	Magnoliophyta	1	1
Haworthia nigra	Magnoliophyta	1	1
Haworthia nortieri	Magnoliophyta	1	1
Haworthia outeniquensis	Magnoliophyta	1	1
Haworthia parksiana	Magnoliophyta	1	1
Haworthia pubescens	Magnoliophyta	1	1
Haworthia pulchella	Magnoliophyta	1	1
Haworthia pumila	Magnoliophyta	1	1
Haworthia pygmaea	Magnoliophyta	1	1
Haworthia reinwardtii	Magnoliophyta	1	1
Haworthia reticulata	Magnoliophyta	1	1
Haworthia retusa	Magnoliophyta	1	1
Haworthia scabra	Magnoliophyta	1	1
Haworthia semiviva	Magnoliophyta	1	1
Haworthia sordida	Magnoliophyta	1	1
Haworthia springbokvlakensis	Magnoliophyta	1	1
Haworthia truncata	Magnoliophyta	1	1
Haworthia variegata	Magnoliophyta	1	1
Haworthia venosa	Magnoliophyta	1	1
Haworthia vlokii	Magnoliophyta	1	1
Haworthia wittebergensis	Magnoliophyta	1	1

Haworthia zantneriana	Magnoliophyta	1	1
Hedera helix	Magnoliophyta	1	1
Hedysarum americanum	Magnoliophyta	1	1
Hedysarum boreale	Magnoliophyta	1	1
Hedyscepe canterburyana	Magnoliophyta	1	1
Helichrysum hyblaeum	Magnoliophyta	1	1
Helicodiceros muscivorus	Magnoliophyta	1	1
Heliconia collinsiana	Magnoliophyta	1	1
Hemithrinax compacta	Magnoliophyta	1	1
Heterospathe elata	Magnoliophyta	1	1
Heterospathe longipes	Magnoliophyta	1	1
Hieracium cophanense	Magnoliophyta	1	1
Hippuris lanceolata	Magnoliophyta	1	1
Homalomena speariae	Magnoliophyta	1	1
Homalomena speariae	Magnoliophyta	1	1
Honckenya peploides	Magnoliophyta	1	1
Hordeum jubatum	Magnoliophyta	1	1
Howea belmoreana	Magnoliophyta	1	1
Howea forsteriana	Magnoliophyta	1	1
Hulteniella integrifolia	Magnoliophyta	1	1
Hydriastele chaunostachys	Magnoliophyta	1	1
Hydriastele costata	Magnoliophyta	1	0
Hydriastele microspadix	Magnoliophyta	1	1
Hydriastele pinangoides	Magnoliophyta	1	0
Hydriastele wendlandiana	Magnoliophyta	1	0
Hyophorbe amaricaulis	Magnoliophyta	0	1
Hyophorbe indica	Magnoliophyta	0	1
Hyophorbe lagenicaulis	Magnoliophyta	1	1
Hyophorbe vaughanii	Magnoliophyta	0	1
Hyophorbe verschaffeltii	Magnoliophyta	0	1
Hyospathe macrorhachis	Magnoliophyta	1	1
Hyphaene coriacea	Magnoliophyta	1	1
Hyphaene dichotoma	Magnoliophyta	1	0
Hyphaene petersiana	Magnoliophyta	1	1
Hyphaene thebaica	Magnoliophyta	1	1
Hypoxidia rhizophylla	Magnoliophyta	1	0
Hypoxis leptocarpa	Magnoliophyta	1	1
Iguanura wallichiana	Magnoliophyta	1	1
Iriartea deltoidea	Magnoliophyta	1	1
Iriartella stenocarpa	Magnoliophyta	1	1
Itaya amicorum	Magnoliophyta	1	1
Johannesteijsmannia altifrons	Magnoliophyta	1	1
Juania australis	Magnoliophyta	1	1
Jubaea chilensis	Magnoliophyta	1	1
Jubaeopsis caffra	Magnoliophyta	1	1
Juncus arcticus	Magnoliophyta	1	0
Juncus biglumis	Magnoliophyta	1	0
Juncus castaneus	Magnoliophyta	1	0
Juncus dudleyi	Magnoliophyta	1	0
Juncus trifidus	Magnoliophyta	1	1
Juncus triglumis	Magnoliophyta	1	1
Kentiopsis oliviformis	Magnoliophyta	1	1
Kerriodoxa elegans	Magnoliophyta	1	1
Kobresia myosuroides	Magnoliophyta	1	1
Kobresia sibirica	Magnoliophyta	1	1
Kobresia simpliciuscula	Magnoliophyta	1	1
Koeleria asiatica	Magnoliophyta	1	1
Korthalsia cheb	Magnoliophyta	1	1
Laccospadix australasica	Magnoliophyta	1	1
Laccosperma acutiflorum	Magnoliophyta	1	1
Lanaria lanata	Magnoliophyta	1	1
Landoltia punctata	Magnoliophyta	1	1
Lasia spinosa	Magnoliophyta	1	1
Latania loddigesii	Magnoliophyta	1	0
Latania lontaroides	Magnoliophyta	1	0
Latania verschaffeltii	Magnoliophyta	1	1
Lathyrus japonicas	Magnoliophyta	1	1
Laurus nobilis	Magnoliophyta	1	1
Lavoixia macrocarpa	Magnoliophyta	1	1
Lemna aequinoctialis	Magnoliophyta	1	1
Lemna disperma	Magnoliophyta	1	1
Lemna ecuadoriensis	Magnoliophyta	1	1
Lemna gibba	Magnoliophyta	1	1
Lemna japonica	Magnoliophyta	1	1
Lemna minor	Magnoliophyta	1	1
Lemna minuta	Magnoliophyta	1	1
Lemna obscura	Magnoliophyta	1	1
Lemna perpusilla	Magnoliophyta	1	1
Lemna tenera	Magnoliophyta	1	1
Lemna trisulca	Magnoliophyta	1	1
Lemna turionifera	Magnoliophyta	1	1

Lemna valdiviana	Magnoliophyta	1	1
Lemna yungensis	Magnoliophyta	1	1
Lemurophoenix halleuxii	Magnoliophyta	1	1
Leopoldinia pulchra	Magnoliophyta	1	1
Lepidorrhachis mooreana	Magnoliophyta	1	1
Leucocasia gigantea	Magnoliophyta	1	1
Leymus mollis	Magnoliophyta	1	1
Licuala beccariana	Magnoliophyta	1	1
Licuala grandis	Magnoliophyta	1	1
Licuala kunstleri	Magnoliophyta	1	1
Limonium lopadusanum	Magnoliophyta	1	0
Limonium panormitanum	Magnoliophyta	1	0
Linaria reflexa	Magnoliophyta	0	1
Linospadix monostachya	Magnoliophyta	1	1
Liparia hirsuta	Magnoliophyta	1	1
Liparia myrtifolia	Magnoliophyta	1	1
Liparia rafnioides	Magnoliophyta	1	1
Liriodendron tulipifera	Magnoliophyta	1	1
Livistona chinensis	Magnoliophyta	1	1
Livistona speciosa	Magnoliophyta	1	0
Lobelia sonderiana	Magnoliophyta	1	0
Lobelia stricta	Magnoliophyta	1	0
Lobelia tomentosa	Magnoliophyta	1	0
Lobelia tupa	Magnoliophyta	1	0
Lodoicea maldivica	Magnoliophyta	1	1
Lomatogonium rotatum	Magnoliophyta	1	1
Loxococcus rupicola	Magnoliophyta	1	1
Lupinus arcticus	Magnoliophyta	1	1
Luzula arcuata	Magnoliophyta	1	0
Luzula confusa	Magnoliophyta	1	0
Luzula kjellmaniana	Magnoliophyta	1	0
Luzula multiflora	Magnoliophyta	1	1
Luzula rufescens	Magnoliophyta	1	0
Luzula wahlenbergii	Magnoliophyta	1	1
Lytocaryum weddellianum	Magnoliophyta	1	1
Magnolia virginiana	Magnoliophyta	1	1
Manicaria saccifera	Magnoliophyta	1	1
Marojejya darianii	Magnoliophyta	1	1
Marojejya insignis	Magnoliophyta	1	1
Masoala kona	Magnoliophyta	1	1
Masoala madagascariensis	Magnoliophyta	1	1
Mauritia flexuosa	Magnoliophyta	1	1
Maxburretia rupicola	Magnoliophyta	1	1
Michelia champaca	Magnoliophyta	1	0
Micranthes nivalis	Magnoliophyta	1	1
Minuartia rubella	Magnoliophyta	1	1
Minuartia stricta	Magnoliophyta	1	1
Molineria capitulata	Magnoliophyta	1	1
Montrichardia arborescens	Magnoliophyta	1	1
Musa campestris	Magnoliophyta	1	1
Musa chunii	Magnoliophyta	1	1
Musa coccinea	Magnoliophyta	1	1
Musa hirta	Magnoliophyta	1	1
Musa ingens	Magnoliophyta	1	1
Myriophyllum sibiricum	Magnoliophyta	1	1
Nassella tenuissima	Magnoliophyta	1	1
Nyctanthes arbor-tristis	Magnoliophyta	1	1
Odontonema tubaeforme	Magnoliophyta	1	0
Oncidium retusum	Magnoliophyta	0	1
Oncidium reversoides	Magnoliophyta	0	1
Oncidium roseoides	Magnoliophyta	0	1
Oncidium sceptrum	Magnoliophyta	0	1
Oncidium schroederianum	Magnoliophyta	0	1
Oncidium serratum	Magnoliophyta	1	1
Oncidium spectatissimum	Magnoliophyta	0	1
Oncidium sphacelatum	Magnoliophyta	0	1
Oncidium stenobulbon	Magnoliophyta	0	1
Oncidium stenoglossum	Magnoliophyta	0	1
Oncidium storkii	Magnoliophyta	0	1
Oncidium tenuoides	Magnoliophyta	0	1
Oncidium toachicum	Magnoliophyta	1	1
Oncidium trinasutum	Magnoliophyta	0	1
Oncostema dimartinoi	Magnoliophyta	1	0
Oncostema hughii	Magnoliophyta	1	1
Orontium aquaticum	Magnoliophyta	1	1
Orthilia secunda	Magnoliophyta	1	1
Oxalis acetosella	Magnoliophyta	1	1
Oxyria digyna	Magnoliophyta	1	1
Oxytropis arctica	Magnoliophyta	1	1
Oxytropis arctobia	Magnoliophyta	1	1
Oxytropis bellii	Magnoliophyta	1	1

Oxytropis borealis	Magnoliophyta	1	1
Oxytropis deflexa	Magnoliophyta	1	1
Oxytropis maydelliana	Magnoliophyta	1	1
Oxytropis varians	Magnoliophyta	1	1
Oxytropis viscida	Magnoliophyta	1	1
Pancratium linosae	Magnoliophyta	1	1
Pancratium maritimum	Magnoliophyta	1	1
Panicum miliaceum	Magnoliophyta	1	1
Panicum sumatrense	Magnoliophyta	1	1
Papaver hultenii	Magnoliophyta	1	1
Parnassia kotzebuei	Magnoliophyta	1	1
Parrya arctica	Magnoliophyta	1	1
Parrya arctica	Magnoliophyta	1	1
Paspalum scrobiculatum	Magnoliophyta	1	0
Pauridia longituba	Magnoliophyta	1	1
Pedicularis albolabiata	Magnoliophyta	1	1
Pedicularis arctoeuropaea	Magnoliophyta	1	1
Pedicularis capitata	Magnoliophyta	1	1
Pedicularis lanata	Magnoliophyta	1	1
Pedicularis langsdorffii	Magnoliophyta	1	1
Pelargonium vitifolium	Magnoliophyta	1	0
Peltandra virginica	Magnoliophyta	1	1
Pennisetum glaucum	Magnoliophyta	1	0
Phillyrea media	Magnoliophyta	1	1
Philodendron hederaceum	Magnoliophyta	1	0
Phippsia concinna	Magnoliophyta	1	1
Physaria arctica	Magnoliophyta	1	1
Pinellia cordata	Magnoliophyta	1	1
Pinellia fujianensis	Magnoliophyta	1	1
Pinellia pedatisecta	Magnoliophyta	1	1
Pinellia peltata	Magnoliophyta	1	1
Pinellia polyphylla	Magnoliophyta	1	1
Pinellia ternata	Magnoliophyta	1	1
Pinguicula vulgaris	Magnoliophyta	1	1
Piptatherum miliaceum	Magnoliophyta	1	1
Pistacia lentiscus	Magnoliophyta	1	1
Pistia stratiotes	Magnoliophyta	1	1
Pleuropogon sabinei	Magnoliophyta	1	1
Poa abbreviata	Magnoliophyta	1	1
Poa alpina	Magnoliophyta	1	1
Poa ammophila	Magnoliophyta	1	1
Poa arctica	Magnoliophyta	1	1
Poa glauca	Magnoliophyta	1	1
Poa hartzii	Magnoliophyta	1	1
Poa pratensis	Magnoliophyta	1	1
Pogonatherum crinitum	Magnoliophyta	1	1
Pogonatherum crinitum	Magnoliophyta	1	1
Polyscias guilfoylei	Magnoliophyta	1	1
Portulacaria afra	Magnoliophyta	1	1
Potentilla anserina	Magnoliophyta	1	1
Potentilla arenosa	Magnoliophyta	1	1
Potentilla biflora	Magnoliophyta	1	1
Potentilla bimundorum	Magnoliophyta	1	1
Potentilla nivea	Magnoliophyta	1	1
Potentilla pulchella	Magnoliophyta	1	1
Potentilla subgorodkovii	Magnoliophyta	1	1
Primula stricta	Magnoliophyta	1	1
Pseuderia smithiana	Magnoliophyta	1	0
Pseudocranichis thysanochila	Magnoliophyta	1	1
Pseudodracontium harmandii	Magnoliophyta	1	1
Pseudodracontium lanceolatum	Magnoliophyta	1	1
Pseudolaelia vellozicola	Magnoliophyta	1	1
Pseudovanilla foliata	Magnoliophyta	1	0
Pseudovanilla ponapensis	Magnoliophyta	1	0
Ptilostemon greuteri	Magnoliophyta	1	1
Puccinellia alaskana	Magnoliophyta	1	1
Puccinellia andersonii	Magnoliophyta	1	1
Puccinellia angustata	Magnoliophyta	1	1
Puccinellia arctica	Magnoliophyta	1	1
Puccinellia bruggemannii	Magnoliophyta	1	1
Puccinellia hauptiana	Magnoliophyta	1	0
Puccinellia nutkaensis	Magnoliophyta	1	1
Puccinellia nuttalliana	Magnoliophyta	1	1
Puccinellia phryganodes	Magnoliophyta	1	1
Puccinellia pumila	Magnoliophyta	1	1
Puccinellia tenella	Magnoliophyta	1	1
Puccinellia vaginata	Magnoliophyta	1	1
Puccinellia vahliana	Magnoliophyta	1	1
Quercus ilex	Magnoliophyta	1	1
Ranunculus arcticus	Magnoliophyta	1	1
Ranunculus lateriflorus	Magnoliophyta	1	1

Remusatia hookeriana	Magnoliophyta	1	1
Remusatia vivipara	Magnoliophyta	1	1
Remusatia yunnanensis	Magnoliophyta	1	1
Rhizophora mucronata	Magnoliophyta	1	1
Rhododendron lapponicum	Magnoliophyta	1	1
Rhododendron tomentosum	Magnoliophyta	1	1
Rhodohypoxis baurii	Magnoliophyta	1	1
Rhodohypoxis milloides	Magnoliophyta	1	1
Rosa sempervirens	Magnoliophyta	1	1
Roystonea regia	Magnoliophyta	1	0
Roystonea regia	Magnoliophyta	1	0
Ruscus aculeatus	Magnoliophyta	1	1
Salix arctica	Magnoliophyta	1	1
Salix arctophila	Magnoliophyta	1	1
Salix glauca	Magnoliophyta	1	1
Salix niphoclada	Magnoliophyta	1	1
Salix reticulata	Magnoliophyta	1	1
Salix richardsonii	Magnoliophyta	1	1
Saxifraga aizoides	Magnoliophyta	1	1
Saxifraga cernua	Magnoliophyta	1	1
Saxifraga cespitosa	Magnoliophyta	1	1
Saxifraga hirculus	Magnoliophyta	1	1
Saxifraga oppositifolia	Magnoliophyta	1	1
Saxifraga tricuspidata	Magnoliophyta	1	1
Scindapsus aureus	Magnoliophyta	1	0
Sesuvium portulacastrum	Magnoliophyta	1	1
Setaria italica	Magnoliophyta	1	1
Setaria viridis	Magnoliophyta	1	1
Silene acaulis	Magnoliophyta	1	1
Silene heuffelii	Magnoliophyta	1	1
Silene involucrata	Magnoliophyta	1	1
Silene ostenfeldii	Magnoliophyta	1	1
Silene uralensis	Magnoliophyta	1	1
Smilax aspera	Magnoliophyta	1	1
Sorghum bicolor	Magnoliophyta	1	1
Sparganium hyperboreum	Magnoliophyta	1	1
Spathiphyllum floribundum	Magnoliophyta	0	1
Spathiphyllum wallisii	Magnoliophyta	1	1
Spiloxene capensis	Magnoliophyta	1	0
Spirodela intermedia	Magnoliophyta	1	1
Stellaria longipes	Magnoliophyta	1	1
Stuckenia filiformis	Magnoliophyta	1	1
Stuckenia pectinata	Magnoliophyta	1	1
Stuckenia vaginata	Magnoliophyta	1	1
Suaeda maritima	Magnoliophyta	1	1
Symphyotrichum pygmaeum	Magnoliophyta	1	1
Syngonium podophyllum	Magnoliophyta	1	1
Syngonium podophyllum	Magnoliophyta	1	1
Taraxacum ceratophorum	Magnoliophyta	1	1
Taraxacum holmenianum	Magnoliophyta	1	1
Taraxacum hyparcticum	Magnoliophyta	1	1
Taraxacum phymatocarpum	Magnoliophyta	1	1
Taraxacum scopulorum	Magnoliophyta	1	1
Tephroseris frigida	Magnoliophyta	1	1
Thamnochortus cinereus	Magnoliophyta	1	1
Tofieldia coccinea	Magnoliophyta	1	1
Tofieldia pusilla	Magnoliophyta	1	1
Tradescantia spathacea	Magnoliophyta	1	1
Tradescantia spathacea	Magnoliophyta	1	1
Trichophorum cespitosum	Magnoliophyta	1	1
Triglochin maritima	Magnoliophyta	1	1
Triglochin palustris	Magnoliophyta	1	1
Tripleurospermum maritimum	Magnoliophyta	1	1
Trisetum spicatum	Magnoliophyta	1	1
Vaccinium uliginosum	Magnoliophyta	1	1
Vaccinium vitis-idaea	Magnoliophyta	1	1
Verbena hastata	Magnoliophyta	1	1
Verbena hastata	Magnoliophyta	1	1
Wrightia religiosa	Magnoliophyta	1	1
Abies chensiensis	Pinophyta	1	1
Abies delavayi	Pinophyta	1	1
Abies densa	Pinophyta	1	1
Abies ernestii	Pinophyta	1	1
Abies fabri	Pinophyta	1	1
Abies fargesii	Pinophyta	1	1
Abies ferrena	Pinophyta	1	1
Abies forrestii	Pinophyta	1	1
Abies georgei	Pinophyta	1	1
Abies holophylla	Pinophyta	1	1
Abies nephrolepis	Pinophyta	1	1
Abies recurvata	Pinophyta	1	1

Abies spectablis	Pinophyta	1	1
Abies squamata	Pinophyta	1	1
Pinus contorta	Pinophyta	1	1
Pinus jeffreyi	Pinophyta	1	1
Pinus muricata	Pinophyta	1	1
Pinus radiata	Pinophyta	1	1
Platycladus orientalis	Pinophyta	1	1
Sphaeropteris glauca	Pteridiophyta	1	0
Asplenium nidus	Pteridiophyta	1	0
Asplenium nitens	Pteridiophyta	1	0
Asplenium normale	Pteridiophyta	1	0
Asplenium northlandicum	Pteridiophyta	1	0
Asplenium obliquissimum	Pteridiophyta	1	0
Asplenium obliquum	Pteridiophyta	1	0
Asplenium oblongifolium	Pteridiophyta	1	0
Asplenium obtusatum	Pteridiophyta	1	0
Botrychium virginianum	Pteridiophyta	1	0
Elaphoglossum lingua	Pteridiophyta	1	0
Elaphoglossum lonchophyllum	Pteridiophyta	1	0
Elaphoglossum luridum	Pteridiophyta	1	0
Elaphoglossum metallicum	Pteridiophyta	1	0
Elaphoglossum minutum	Pteridiophyta	1	0
Elaphoglossum moorei	Pteridiophyta	1	0
Elaphoglossum nigrocostatum	Pteridiophyta	1	0
Elaphoglossum orbignyanum	Pteridiophyta	1	0
Nephrolepis exaltata	Pteridiophyta	1	0
Sphaeropteris albifrons	Pteridiophyta	1	0
Sphaeropteris atrox	Pteridiophyta	1	0
Sphaeropteris auriculifera	Pteridiophyta	1	0
Sphaeropteris brunei	Pteridiophyta	1	0
Sphaeropteris capitata	Pteridiophyta	1	0
Sphaeropteris celebica	Pteridiophyta	1	0
Sphaeropteris cooperi	Pteridiophyta	1	0
Sphaeropteris excelsa	Pteridiophyta	1	0
Sphaeropteris horrida	Pteridiophyta	1	0

Supplementary Table S2. Species pairs used to retrieve full-length barcodes from chloroplast genomes and barcode databases. List of 101 generic species pairs for which sequence data of *mat*K and *rbc*L loci were downloaded from complete chloroplast genomes from NCBI, and from barcoding entries of BOLD.

	Generic species pair	NCBI chloroplast genome accession number		BOLD accession numbers: matK	BOLD accession numbers: rbcL
1	*Acorus calamus*	NC_007407.1	AJ879453	CPBOL159-11, CPBOL161-11, CPBOL163-11, GBVA1508-11, GBVA1510-11, GBVA1511-11, GBVA1512-11, GBVA1516-11, GBVA1518-11, GBVB782-11, GBVB783-11, GBVB784-11, GBVB785-11, GBVR1770-13, GBVS384-13, GBVS388-13, GBVT1243-13, GBVT1244-13, GBVT1245-13, GBVT1246-13, GBVT1247-13, GBVT1248-13, GBVT1249-13, GBVT1250-13, GBVX1809-13	CPBOL159-11, CPBOL161-11, GBVA1509-11, GBVA1513-11, GBVA1514-11, GBVA1515-11, GBVA1517-11, GBVA1519-11, GBVA1520-11, GBVA1521-11, GBVA1522-11, GBVB786-11, GBVB787-11, GBVR1770-13, GBVT1160-13, GBVT1161-13, GBVT1162-13, GBVT1163-13, GBVT1164-13, GBVT1165-13, GBVT1166-13, GBVT1167-13, GBVX1809-13, GBVX6401-15, GBVY136-14, GBVY139-14
	Acorus gramineus	NC_026299.1	KP099646	CPBOL165-11, CPBOL167-11, CPBOL169-11, GBVA1523-11, GBVA1525-11, GBVA1529-11, GBVB789-11, GBVB790-11, GBVB791-11, GBVB792-11, GBVB793-11, GBVB794-11, GBVT1227-13, GBVT1228-13, GBVT1229-13, GBVT1230-13, GBVX7607-15	CPBOL165-11, CPBOL167-11, CPBOL169-11, GBVA1524-11, GBVA1526-11, GBVA1527-11, GBVA1528-11, GBVA1530-11, GBVA1531-11, GBVB795-11, GBVS3837-13, GBVT1143-13, GBVT1144-13, GBVT1145-13, GBVT1146-13, GBVX7607-15
2	*Actinidia chinensis*	NC_026690.1	KP297242	GBVK4698-11, GBVK4699-11	GBVK4700-11, GBVK4701-11, GBVK4702-11, GBVK4703-11, GBVK4704-11
	Actinidia deliciosa	NC_026691.1	KP297244	GBVK4710-11, GBVK4626-13	
3	*Aegilops sharonensis*	NC_024816.1	KJ614419	GBVD2616-11, GBVD2617-11, GBVD2618-11, GBVD2619-11, GBVD2620-11, GBVX6343-15, GBVX6345-15, GBVX7862-15	GBVX6343-15. GBVX6345-15, GBVX7862-15
	Aegilops speltoides	NC_022135.1	JQ740834	GBVD2621-11, GBVD2622-11, GBVD2624-11, GBVD2625-11, GBVD2626-11, GBVD2627-11, GBVD2628-11, GBVD2629-11, GBVD2630-11, GBVD2631-11, GBVD2632-11	GBVD2623-11, GBVX7803-15
4	*Aethionema cordifolium*	NC_009265.1	AP009366	GBVR1892-13, GBVX1842-13	GBVR1892-13, GBVX1842-13
	Aethionema grandiflorum	NC_009266.1	AP009367	GBVE3046-11, GBVR1893-13, GBVX1843-13	GBVE3047-11, GBVR1893-13, GBVX1843-13
5	*Amentotaxus argotaenia*	NC_027581.1	KR780582	CPBOL308-11, CPBOL310-11, CPBOL312-11, GBVM1032-11, GBVM1033-11, GBVM1034-11, GBVQ1399-13, GBVQ2599-13	CPBOL308-11, CPBOL310-11, CPBOL312-11, GBVM1031-11, GBVM1035-11, GBVM1036-11, GBVM1037-11, GBVQ1438-13, GBVQ2723-13
	Amentotaxus formosana	NC_024945.1	AP014574	CPBOL314-11, GBVM1039-11, GBVX7907-15, GBVX7959-15	CPBOL314-11, GBVM1038-11, GBVX7907-15, GBVX7959-15
6	*Angiopteris angustifolia*	NC_026300.1	KP099647	GBVX8013-15, GBVX8038-15	GBVM1403-11, GBVX8013-15, GBVX8038-15
	Angiopteris evecta	NC_008829.1	DQ821119	GBVQ568-13, GBVQ4540-13	GBVM1408-11, GBVM1409-11, GBVM1410-11, GBVM1411-11, GBVM1412-11, GBVM1413-11, GBVQ568-13, GBVQ4540-13
7	*Angophora costata*	NC_022412.1	KC180805	GBVH4871-11, GBVX2220-15, GBVX2342-15, GBVX2460-15, GBVX3631-15	GBVH4870-11
	Angophora floribunda	NC_022411.1	KC180804	GBVX2286-15, GBVX2446-15, GBVX3632-15	
8	*Anthoxanthum nitens*	NC_027475.1	KM974740	GBVR4252-13, VEMSH665-13	GBVD2850-11, GBVS3326-13, GBVW2857-13, VEMSH665-13
	Anthoxanthum odoratum	NC_027467.1	KM974732	GBVD2851-11, GBVD2853-11, GBVR1774-13, GBVW1877-13, GRASS951-07, GRASS1261-07, GRASS1273-07, POWNA040-10, POWNA1027-12, POWNA1030-12	GBVD2852-11, GBVD2854-11, GBVD2855-11, GBVY479-14, GBVY481-14, GBVY483-14, GBVY485-14, GRASS951-07, GRASS1261-07, GRASS1273-07, POWNA040-10, POWNA1027-12, POWNA1030-12
9	*Arabis alpina*	NC_023367.1	HF934132	FCA1857-11, FCA1858-11, FCA1859-11, FCA2022-11, GBVE3100-11, GBVE3101-11, GBVP2280-14, IASVF236-09, MKPCH716-10, MKPCH717-10	FCA1857-11, FCA1858-11, FCA1859-11, FCA1860-11, FCA2022-11, GBVP2280-14, IASVF236-09, MKPCH716-10, MKPCH717-10
	Arabis hirsuta JO23	NC_009268.1	AP009369	GBVE3103-11, GBVE3105-11, GBVR1895-13, GBVX1845-13, POWNA049-10, POWNA1065-12, POWNA1066-12	GBVE3104-11, GBVR1895-13, GBVW2805-13, GBVX1845-13, POWNA049-10, POWNA1065-12, POWNA1066-12
10	*Arundinaria fargesii*	NC_024712.1	JX513413	GBVT2008-13, GBVT2009-13, GBVT2010-13, GBVT2011-13, GBVX4872-15, GBVX7838-15	GBVT2103-13, GBVT2104-13, GBVT2105-13, GBVT2106-13, GBVX4872-15, GBVX7838-15

	Arundinaria tecta	NC_023935.1	KC817463	GBVD2892-11	GBVD2891-11
11	*Asclepias nivea*	NC_022431.1	KF539844		
	Asclepias syriaca	NC_022432.1	KF386166	GBVG9573-11, GBVS2867-13, GBVS4349-13, KSR133-07, VEMSH237-13, WABLK367-13, WABLK478-13	GBVG574-11, GBVS2935-13, GBVS4349-13, GBVY625-14, KSR133-07, VEMSH237-13
12	*Bambusa bambos*	NC_026957.1	KJ870988	GBVD3012-11, GBVW3643-13, GBVW3646-13	GBVX1713-13, GENG291-14, TDEF061-12, TDEF062-12, TDEF063-12
	Bambusa emeiensis	NC_015830.1	HQ337797	GBVS1654-13, GBVS2807-13, GBVX1918-13	GBVD3014-11, GBVS2807-13, GBVX1918-13
13	*Brassica napus ZY036*	NC_016734.1	GQ861354	GBVE3176-11, GBVE3178-11, GBVE3179-11, GBVE3180-11, GBVE3181-11, GBVE3182-11, GBVR4446-13, GBVT318-13, GBVX1945-13, GBVX6570-15	GBVE3177-11, GBVE3183-11, GBVE3184-11, GBVE3185-11, GBVE3186-11, GBVR4446-13, GBVX1945-13, GBVX6570-15, GBVX6647-15
	Brassica rapa	NC_015139.1	DQ231548	GBVE3203-11, GBVE3204-11, POWNA081-10, POWNA1424-12	GBVX6649-15, POWNA081-10, POWNA1424-12
14	*Callitropsis nootkatensis*	NC_026295.1	KP099642	GBVL4070-11, GBVL4072-11, GBVL4073-11, GBVQ1120-13, GBVX7929-15, GBVX7930-15, GBVX7953-15	GBVL4071-11, GBVL4074-11, GBVX7939-15, GBVX7940-15, GBVX7953-15
	Callitropsis vietnamensis	NC_026298.1	KP099645	GBVQ1156-13, GBVX7956-15	GBVX7956-15
15	*Camellia crapnelliana*	NC_024541.1	KF753632	GBVP6306-15	GBVP6306-15
	Camellia grandibracteata	NC_024659.1	KJ806274	GBVX5812-15, GBVX5830-15	GBVX5843-15, GBVX7830-15
16	*Capsella bursa-pastoris JO22*	NC_009270.1	AP009371	GBVE3229-11, GBVE3231-11, GBVR1897-13, GBVX1847-13, KSR175-07, MKPCH422-09	GBVE3230-11, GBVE3232-11, GBVE3233-11, GBVE3234-11, GBVE3235-11, GBVR1897-13, GBVT364-13, GBVW2807-13, GBVX1847-13, GBVY1204-14, KSR175-07, MKPCH422-09, PLGE128-13, POWNB062-10, POWNB230-10, VEMSH177-13
	Capsella rubella	NC_027693.1	KR029093	GBVE3243-11, GBVE3245-11, GBVE3246-11, GBVE3247-11, GBVE3248-11, GBVE3249-11, GBVE3250-11, GBVE3251-11, GBVE3252-11, GBVE3253-11, GBVE3254-11, GBVE3255-11, GBVE3256-11, GBVE3257-11, GBVE3258-11, GBVE3259-11, GBVE3260-11, GBVE3261-11, GBVE3262-11, GBVE3263-11, GBVE3264-11, GBVE3265-11, GBVE3266-11, GBVE3267-11, GBVE3268-11, GBVE3269-11, GBVE3270-11	GBVE3244-11
17	*Capsicum annuum*	NC_018552.1	JX270811	GBVK3996-11, GBVR914-13, GBVR916-13, GBVR918-13, GBVR920-13, GBVR922-13, GBVR924-13, GBVR926-13, GBVR928-13, GBVR930-13, GBVR932-13, GBVR934-13, GBVR936-13, GBVR938-13, GBVR940-13, GBVR942-13, GBVR944-13, GBVR946-13, GBVR948-13, GBVR950-13, GBVR952-13, GBVR954-13, GBVR956-13, GBVR958-13, GBVR960-13, GBVR962-13, GBVR964-13, GBVR966-13, GBVR968-13, GBVR970-13, GBVR972-13, GBVR974-13, GBVR976-13, GBVR978-13, GBVR980-13, GBVT1372-13, GBVW1482-13, GBVX1964-13, GBVX6351-15	GBVR915-13, GBVR917-13, GBVR919-13, GBVR921-13, GBVR923-13, GBVR925-13, GBVR927-13, GBVR929-13, GBVR931-13, GBVR933-13, GBVR935-13, GBVR937-13, GBVR939-13, GBVR941-13, GBVR943-13, GBVR945-13, GBVR947-13, GBVR951-13, GBVR953-13, GBVR955-13, GBVR957-13, GBVR959-13, GBVR961-13, GBVR963-13, GBVR965-13, GBVR967-13, GBVR969-13, GBVR971-13, GBVR973-13, GBVR975-13, GBVR977-13, GBVR979-13, GBVR981-13, GBVS1605-13, GBVT1422-13, GBVW1482-13, GBVW2962-13, GBVX1964-13, GBVX6363-15, GBVX7405-15, GBVY1206-14, GBVY1208-14, GBVY1210-14, GBVY1212-14, GBVY1214-14, HIDNA035-14
	Capsicum lycianthoides	NC_026551.1	KP274856		
18	*Cardamine impatiens*	NC_026445.1	KJ136821	CPBOL690-11, CPBOL692-11, CPBOL694-11, GBVX5723-15, POWNA103-10	CPBOL690-11, CPBOL692-11, CPBOL694-11, GBVX5723-15, POWNA103-10, POWNA1891-12
	Cardamine resedifolia	NC_026446.1	KJ136822	GBVX5724-15	GBVX5724-15
19	*Cephalotaxus oliveri*	NC_021110.1	KC136217	CPBOL804-11, GBVL4036-11, GBVQ4073-13, GBVQ4595-13	CPBOL804-11, GBVL4037-11, GBVL4038-11, GBVQ4073-13, GBVQ4595-13
	Cephalotaxus wilsoniana	NC_016063.1	AP012265	GBVL4052-11, GBVQ512-13, GBVQ4579-13	GBVL4053-11, GBVL4054-11, GBVL4055-11, GBVQ512-13, GBVQ4579-13
20	*Chloranthus japonicus*	NC_026565.1	KP256024		GBVI1216-11
	Chloranthus spicatus	NC_009598.1	EF380352	GBVR2630-13, GBVX1853-13	GBVI1220-11, GBVR2630-13, GBVX1853-13
21	*Chrysanthemum indicum*	NC_020320.1	JN867589	GBVT4121-13, GBVT4122-13, GBVX1983-13	GBVT338-13, GBVT4121-13, GBVT4122-13, GBVX1983-13, GENG1119-15
	Chrysanthemum x morifolium	NC_020092.1	JQ362483	GBVN3047-11, GBVN3048-11, GBVU573-13, GBVX1977-13	GBVT339-13, GBVU573-13, GBVX1977-13, GBVX7081-15

22	*Chusquea circinata*	NC_027490.1	KP319241		GBVD3202-11
	Chusquea liebmannii	NC_026969.1	KJ871001	GBVD3205-11	GBVD3206-11
23	*Cistanche deserticola*	NC_021111.1	KC128846	GBVW3974-13, GBVX1997-13	
	Cistanche phelypaea	NC_025642.1	HG515538	GBVK2930-11, GBVX4854-15, GBVX7886-15	GBVK2929-11
24	*Citrus aurantiifolia*	NC_024929.1	KJ865401	GBVK859-11, GBVR297-13, GBVX6569-15GBVX7866-15, TDEF422-12, TDEF423-12	GBVK860-11, GBVX6569-15, GBVX7866-15, TDEF421-12, TDEF422-12, TDEF423-12
	Citrus sinensis	NC_008334.1	DQ864733	GBVK919-11, GBVK920-11, GBVR310-13, GBVR1553-13, GBVR2518-13, GBVX1826-13, GBVX4786-15, GBVX4822-15	GBVK921-11, GBVK922-11, GBVK923-11, GBVR2518-13, GBVX1671-13, GBVX1826-13, GBVX4826-15, GENG1072-15
25	*Corallorhiza mertensiana*	NC_025661.1	KM390018	GBVA4212-11, GBVP5473-15, GBVP6873-15	GBVA4211-11
	Corallorhiza odontorhiza	NC_025664.1	KM390021	GBVA4214-11, GBVX7376-15, GBVX7890-15	GBVA4213-11, GBVA4215-11, GBVX7376-15, GBVX7890-15
26	*Corymbia eximia*	NC_022409.1	KC180802		
	Corymbia maculata	NC_022408.1	KC180801	GBVH4944-11	
27	*Cucumis melo*	NC_015983.1	JF412791	GBVL1271-11, GBVL1273-11, GBVT686-13, GBVT687-13, GBVT688-13, GBVT689-13, GBVT690-13, GBVT691-13, GBVT692-13, GBVT693-13, GBVT694-13, GBVT695-13, GBVT696-13	GBVL1272-11, GBVL1274-11, GENG616-14
	Cucumis sativus	NC_007144.1	AJ970307	GBVT681-13, GBVT683-13, GBVR2519-13, GBVL1293-11, GBVT680-13, GBVX1808-13, GBVT682-13, GBVR2395-13, GBVT678-13, GBVR1771-13, GBVT684-13, GBVT677-13, GBVT679-13, GBVR2520-13	GBVL1290-11, GBVL1291-11, GBVR1771-13, GBVR2395-13, GBVR2519-13, GBVR2520-13, GBVX1808-13, GBVY1700-14
28	*Cuscuta exaltata*	NC_009963.1	EU189132	GBVX1863-13	GBVX1863-13
	Cuscuta reflexa	NC_009766.1	AM711640	GBVK3495-11, GBVR1867-13, GBVX1858-13	GBVK3496-11, GBVR1867-13, GBVX1858-13
29	*Cycas revoluta*	NC_020319.1	JN867588	GBVB328-11, GBVQ2273-13, GBVQ2618-13, GBVQ2619-13, GBVQ4590-13	GBVB329-11, GBVB330-11, GBVB331-11, GBVP3658-15, GBVQ1763-15, GBVQ1764-15, GBVQ2273-13, GBVQ2742-13, GBVQ2743-13
	Cycas taitungensis	NC_009618.1	AP009339	GBVB339-11, GBVB340-11, GBVQ504-13, GBVQ4542-13	GBVQ504-13, GBVQ4542-13
30	*Cymbidium faberi*	NC_027743.1	KR919606	GBVX3826-15, GBVX3827-15	GBVX3857-15, GBVX3858-15
	Cymbidium sinense	NC_021430.1	KC876123	GBVA4475-11, GBVX1232-13, GBVX2004-13, GBVX3839-15, GBVX3840-15, GBVX3841-15, GBVX3842-15, GBVY235-14, GBVY879-14, GBVY1335-14, GBVY1372-14, GBVY1375-14	GBVX1232-13, GBVX2004-13, GBVX3870-15, GBVX3871-15, GBVX3872-15, GBVX3873-15
31	*Cypripedium japonicum*	NC_027227.1	KJ625630	GBVT1682-13, GBVX5109-15	GBVB2685-11, GBVT1698-13, GBVX1052-13, GBVY1761-14
	Cypripedium macranthos	NC_024421.1	KF925434	GBVX790-13, GBVX5110-15, GBVX5390-15, GBVX7826-15	GBVB2686-11, GBVX1053-13, GBVX1054-13, GBVX1055-13, GBVX5390-15, GBVX7826-15, GBVY1763-14
32	*Dendrobium catenatum*	NC_024019.1	KC771275	GBVP4127-15, GBVP4128-15, GBVX1430-13, GBVX1431-13, GBVX1432-13, GBVX1433-13, GBVX6568-15	GBVX6568-15
	Dendrobium strongylanthum	NC_027691.1	KR673323	GBVX1502-13, GBVX1503-13	
33	*Dendropanax dentiger*	NC_026546.1	KP271241	GBVE1586-11	GBVE1587-11
	Dendropanax morbifer	NC_027607.1	KR136270		
34	*Dioscorea rotundata*	NC_024170.1	KJ490011	GBVC2111-11, GBVX2909-15, GBVX5964-15, GBVX7820-15	GBVC2112-11, GBVX5964-15, GBVX7820-15
	Dioscorea zingiberensis	NC_027090.1	KP899622	GBVC2148-11, GBVT4937-13, GBVT4934-13, GBVT4931-13, GBVT4929-13, GBVP2960-14, GBVP2959-14, GBVP2962-14, GBVT4932-13, GBVP2963-14, GBVT4936-13, GBVT4933-13, GBVP2961-14, GBVT4930-13, GBVT4935-13, GBVT4938-13	GBVP3059-14, GBVP3060-14, GBVP3061-14, GBVP3062-14, GBVP3063-14, GBVP3064-14, GBVP3065-14, GBVP3066-14, GBVP3067-14, GBVU077-13, GBVU078-13, GBVU079-13, GBVU080-13, GBVU081-13, GBVU082-13, GBVU083-13, GBVU084-13, GBVU085-13, GBVU086-13

35	*Dunalia obovata*	NC_026563.1	KP280057		
	Dunalia solanacea	NC_027099.1	KP998157		
36	*Equisetum arvense*	NC_014699.1	GU191334	GBVB445-11, GBVB446-11, GBVB447-11, GBVQ706-13, GBVQ2335-13, GBVQ4573-13	GBVA1036-11, GBVA1037-11, GBVA1038-11, GBVA1039-11, GBVB448-11, GBVQ2335-13, GBVQ4573-13, GBVQ706-13, GBVX8012-15, IASVF132-09, IASVF150-09, KSR529-08, PLGE138-13, RRPLA061-15, VEMSH503-13, VEMSH504-13, VEMSH508-13, WABLK462-13
	Equisetum hyemale	NC_020146.1	KC117177	GBVA1050-11, GBVA1051-11, GBVP1735-14, GBVP1734-14, GBVQ4070-13, GBVP1730-14, GBVP1737-14, GBVP1733-14, GBVQ1000-13, GBVP1729-14, GBVA1052-11, GBVB451-11, GBVP1731-14, GBVA1053-11, GBVP1732-14, GBVP1736-14, GBVB452-11, GBVQ4588-13, GBVP1738-14	GBVA1046-11, GBVA1047-11, GBVA1048-11, GBVA1049-11, GBVA1054-11, GBVA1055-11, GBVP1739-14, GBVP1740-14, GBVP1741-14, GBVP1742-14, GBVP1743-14, GBVP1744-14, GBVP1745-14, GBVP1746-14, GBVP1747-14, GBVP1748-14, GBVQ1562-13, GBVQ4070-13, GBVQ4588-13
37	*Erodium carvifolium*	NC_015083.1	HQ713469	GBVS5367-13, GBVX1901-13	GBVS3567-13, GBVX1901-13
	Erodium texanum	NC_014569.1	HM125536	GBVG1771-11, GBVS1633-13, GBVX1893-13	GBVG1772-11, GBVG1773-11, GBVS1633-13, GBVX1893-13
38	*Eucalyptus globulus subsp. globulus*	NC_008115.1	AY780259	GBVH4959-11, GBVH4961-11, GBVH4962-11, GBVH4965-11, GBVH4966-11, GBVH4967-11, GBVX2260-15, GBVX2277-15, GBVX2279-15, GBVX2284-15, GENG050	GBVH4960-11, GBVH4963-11, GBVH4964-11, GBVX1689-13, GENG018-11, GENG021-11, GENG088-12
	Eucalyptus grandis	NC_014570.1	HM347959	GBVS1637-13, GBVX1844-13	GBVH4969-11, GBVS1637-13, GBVX1894-13
39	*Fagopyrum esculentum subsp. ancestrale*	NC_010776.1	EU254477	GBVI3462-11, GBVI3462-11, GBVT064-13, GBVT759-13	GBVI3461-11, GBVI3463-11, GBVI3465-11, GBVR0008-13, GBVT1705-13
	Fagopyrum tataricum Daegwan3-3	NC_027161.1	KM201427	GBVI3498-11, GBVI3499-11, GBVI3500-11, GBVT067-13, GBVT758-13	GBVI3502-11, GBVR018-13, GBVT1706-13
40	*Fargesia nitida*	NC_024715.1	JX513416	GBVP2884-14, GBVX4875-15, GBVX7841-15, GRASS1406-10	GBVX4875-15, GBVX7841-15, GRASS1406-10
	Fargesia yunnanensis	NC_024717.1	JX513418	GBVT2035-13, GBVT2036-13, GBVT2037-13, GBVX4877-15, GBVX7843-15	GBVT2130-13, GBVT2131-13, GBVT2132-13, GBVX4877-15, GBVX7843-15
41	*Festuca arundinacea*	NC_011713.2	FJ466687	GBVD3484-11, GBVD3486-11, GBVP6640-15, GBVP6685-15, GBVR2903-13, GBVW1898-13, GBVX1877-13, KSR314-07, PLGE124-13, POWNA1676-12, POWNA1678-12	GBVD3485-11, GBVR2903-13, GBVX1877-13, GBVY2159-14, GBVY2161-14, PLGE124-13, POWNA1676-12, POWNA1678-12
	Festuca pratensis	NC_019650.1	JX871941	GBVD3494-11, GBVP6666-15, GBVW3313-13, GBVX1974-13, POWNA290-10, POWNA1549-12, POWNA1681-12	GBVD3495-11, GBVD3496-11, GBVW3313-13, GBVX1974-13, GBVY2168-14, POWNA290-10, POWNA1549-12, POWNA1681-12
42	*Fragaria chiloensis*	NC_019601.1	JN884816	GBVT4225-13, GBVX1969-13	GBVT4225-13, GBVW1809-13, GBVX1969-13
	Fragaria vesca subsp. vesca	NC_015206.1	JF345175	GBVJ2287-11, GBVJ2290-11, GBVJ2291-11, POWNA297-10, POWNA1014-12, POWNA1143-12, POWNA2129-12, SCBI526-14	GBVJ2288-11, GBVJ2289-11, GBVJ2292-11, POWNA297-10, POWNA1014-12, POWNA11433-12, POWNA2129-12, SCBI526-14
43	*Fritillaria hupehensis*	NC_024736.1	KF712486	GBVC3498-11, GBVX5359-15, GBVX7856-15	GBVX5359-15, GBVX7856-15
	Fritillaria taipaiensis	NC_023247.1	KC543997	GBVX5368-15	GBVX5368-15
44	*Gentiana crassicaulis*	NC_027442.1	KJ676538	GBVX2914-15, GBVX2915-15, GBVX2916-15, GBVX2917-15, GBVX2918-15, GBVX2919-15, GBVX29204-15, GBVX2921-15, GBVX2922-15,	GBVX2960-15, GBVX2961-15, GBVX2962-15, GBVX2963-15, GBVX2964-15, GBVX2965-15, GBVX2966-15, GBVX2967-15, GBVX2968-15
	Gentiana straminea	NC_027441.1	KJ657732	GBVX2923-15, GBVX2924-15, GBVX2925-15, GBVX2974-15	GBVG1481-11, GBVX2969-15, GBVX2970-15, GBVX2971-15, GBVX3276-15
45	*Glycine falcata*	NC_021649.1	KC893637	GBVX2014-13	GBVX2014-13
	Glycine max	NC_007942.1	DQ317523	GBVO203-11, GBVO205-11, GBVO206-11, GBVO207-11, GBVO208-11, GBVO209-11, GBVO210-11, GBVO211-11, GBVO212-11, GBVO213-11, GBVR2412-13, GBVX1815-13	GBVO204-11, GBVO215-11, GBVO216-11, GBVO217-11, GBVO218-11, GBVO219-11, GBVO220-11, GBVO221-11, GBVO222-11, GBVO223-11, GBVR2412-13, GBVT3577-13, GBVX1815-13
46	*Gnetum montanum*	NC_021438.1	KC427271	GBVB561-11, GBVQ4239-13, GBVQ4597-13	GBVA1227-11, GBVB62-11, GBVQ4239-13
	Gnetum parvifolium	NC_011942.1	AP009569	GBVB563-11, GBVQ507-13, GBVQ4556-13	GBVA1232-11, GBVA1233-11, GBVB564-11, GBVB565-11, GBVB566-11, GBVQ507-13, GBVQ4556-13

47	*Gossypium darwinii*	NC_016670.1	HQ325741	GBVS2669-13, GBVX1932-13, MAPLT101-10, MAPLT102-10, MAPLT103-10, MAPLT104-10, MAPLT105-10	GBVS2669-13, GBVX1932-13, MAPLT101-10, MAPLT102-10, MAPLT103-10, MAPLT104-10, MAPLT105-10
	Gossypium mustelinum	NC_016711.1	HQ325743	GBVS2671-13, GBVX1938-13, MAPLT096-10, MAPLT097-10, MAPLT098-10, MAPLT099-10, MAPLT100-10	GBVS2671-13, GBVX1938-13, MAPLT096-10, MAPLT097-10, MAPLT098-10, MAPLT099-10, MAPLT100-10
48	*Haloxylon ammodendron*	NC_027668.1	KF534478		GBVE4030-11
	Haloxylon persicum	NC_027669.1	KF534479	GBVR4051-13	GBVE4031-11, GBVE4069-13
49	*Helianthus divaricatus*	NC_023109.1	KF746352	SCBI483-14, SCBI486-14	SCBI483-14, SCBI486-14
	Helianthus tuberosus	NC_023112.1	KF746361	GBVO501-11, GBVO504-11, GBVR4569-13	GBVO502-11, GBVO503-11, GBVP2952-14
50	*Hirtella physophora*	NC_024066.1	KJ414485	GBVX7817-15	GBVX7817-15
	Hirtella racemosa	NC_024060.1	KJ414479		
51	*Hordeum jubatum*	NC_027476.1	KM974741	GBVD3649-11, GBVD3650-11, GBVX4440-15, GBVX4441-15, GRASS730-07, GRASS739-07, GRII010-08	GBVD3651-11, GBVW2870-13, GRASS730-07, GRASS739-07, GRII010-08
	Hordeum vulgare subsp. vulgare	NC_008590.1	EF115541	GBVD3754-11, GBVD3755-11, GBVR2597-13, GBVX1379-13, GBVX1834-13	GBVD3756-11, GBVR2597-13, GBVS3319-13, GBVX1379-13, GBVX1834-13
52	*Indocalamus longiauritus*	NC_015803.1	HQ337795	GBVD33779-11, GBVS2805-13, GBVT2041-13, GBVT2042-13, GBVT2043-13, GBVT2044-13, GBVT2045-13, GBVT2046-13, GBVX1914-13	GBVS2805-13, GBVT2136-13, GBVT2137-13, GBVT2138-13, GBVT2139-13, GBVT2140-13, GBVT2141-13, GBVX1914-13
	Indocalamus wilsonii	NC_024720.1	JX513421	GBVX4880-15, GBVX7846-15	GBVX4880-15, GBVX7846-15
53	*Iochroma stenanthum*	NC_026574.1	KP262399		
	Iochroma tingoanum	NC_027177.1	KP280175		
54	*Ipomoea batatas*	NC_026703.1	KP212149	GBVK3552-11, GBVW2458-13, GBVW2459-13	GBVK3553-11, GBVU4115-13, GBVW013-13, GBVW014-13, GBVW752-13
	Ipomoea purpurea	NC_009808.1	EU118126	GBVK3577-11, GBVR2659-13, GBVX1859-13	GBVK3574-11, GBVK3575-11, GBVK3576-11, GBVR2659-13, GBVX1859-13
55	*Juniperus bermudiana*	NC_024021.1	KF866297	GBVL4256-11	GBVL4257-11
	Juniperus scopulorum	NC_024023.1	KF866299	GBVL4391-11	GBVL4392-11
56	*Lathyrus clymenum*	NC_027148.1	KJ850235	GBVO1158-11, GBVW2225-13	GBVO1159-11
	Lathyrus venosus	NC_027080.1	KJ806202	GBVW2254-13	GBVW2180-13
57	*Licania alba*	NC_024064.1	KJ414483	GBVU4006-13	GBVJ1499-11, GBVJ1500-11, GBVJ1501-11, GBVU1761-13
	Licania sprucei	NC_024065.1	KJ414484	GBVU4013-13	GBVU2016-13
58	*Lilium hansonii*	NC_027674.1	KM103364	GBVC3619-11, GBVC3620-11, GBVS4354-13, GBVS4355-13, GBVS4356-13, GBVS4357-13, GBVT2676-13	GBVC3621-11, GBVT2765-13, GBVW3511-13
	Lilium superbum	NC_026787.1	KP462883	GBVC3686-11, GBVC3687-11, GBVS2065-13	GBVC3688-11, GBVC3689-11, GBVS2104-13
59	*Lolium multiflorum*	NC_019651.1	JX871942	GBVP6667-15, GBVR3374-13, GBVW3314, GBVX1975-13, GRASS977-07, PLGE092-13	GBVR3166-13, GBVS3336-13, GBVW3314-13, GBVX1975-13, GBVY2704-14, GRASS977-07, PLGE092-13

	Lolium perenne	NC_009950.1	AM777385	GBVD3985-11, GBVD387-11, GBVD3988-11, GBVD3989-11, GBVR1869-13, GBVS1365-13, GBVW1902-13, GBVX1861-13, GRASS808-07, GRASS986-07, GRASS1020-07, PLGE016-13, POWNA434-10, POWNA1651-12, POWNA2644-12, POWNA2645-12	GBVD3986-11, GBVD3990-11, GBVR1869-13, GBVS929-13, GBVS3313-13, GBVX1861-13, GBVY2705-14, GRASS808-07, GRASS986-07, GRASS1020-07, KSR116-07, PLGE016-13, POWNA434-10, POWNA2644-12, POWNA2645-12
60	*Lupinus albus*	NC_026681.1	-		GBVO1557-11, GBVO1558-11
	Lupinus luteus	NC_023090.1	KC695666	GBVO1575-11	GBVO1576-11, GBVO1577-11
61	*Magnolia grandiflora*	NC_020318.1	JN867584	GBVH1380-11, GBVX1982-13, GBVH1376-11, GBVT810-13, GBVX6048-15, GBVT4120-13, GBVH1389-11, GBVT811-13, GBVT809-13, GBVH1383-11, HOSAM069-10, GBVH1382-11, GBVT4119-13, GBVH1384-11, GBVH1378-11	GBVH1377-11, GBVH1379-11, GBVH1381-11, GBVH1385-11, GBVH1386-11, GBVH1387-11, GBVH1388-11, GBVH1390-11, GBVT4119-13, GBVT4120-13, GBVT889-13, GBVT890-13, GBVT891-13, GBVX1982-13, GBVX5881-15, GENG531-14, HOSAM069-10
	Magnolia kwangsiensis	NC_015892.1	HM775382	GBVH1408-11, GBVH1409-11, GBVH1410-11, GBVH1411-11, GBVH1504-11, GBVS1791-13, GBVT839-13, GBVT853-13, GBVX1921-13	GBVH1412-11, GBVS1791-13, GBVX1921-13
62	*Masdevallia coccinea*	NC_026541.1	KP205432		
	Masdevallia picturata	NC_026777.1	KJ566305		
63	*Medicago hybrida*	NC_027153.1	KJ850240	GBVO1726-11	
	Medicago papillosa	NC_027154.1	KJ850241	GBVO1748-11	
64	*Melica mutica*	NC_027477.1	KM974742	GBVD4024-11	GBVD4023-11
	Melica subulata	NC_027478.1	KM974743		GRASS972-07, GRASS991-07, GRASS1028-07, GRASS1040-07, GRASS1210-07, GRASS1249-07
65	*Morus indica*	NC_008359.1	DQ226511	GBVL516-11, GBVR2397-13, GBVX1829-13, GBVX6296-15, GBVX6298-15, GBVX6300-15, GBVX6302-15, GBVX6303-15, GBVX6305-15, GBVX6306-15	GBVL517-11, GBVR2397-13, GBVX1829-13
	Morus mongolica	NC_025772.2	KM491711	GBVX7406-15, GBVX7895-15	GBVX7406-15, GBVX7895-15
66	*Nelumbo lutea*	NC_015605.1	FJ754269	GBVI1010-11, GBVR2912-13, GBVU508-13, GBVX1909-13	GBVI1009-11, GBVI1011-11, GBVI1012-11, GBVR2912-13, GBVU508-13, GBVX1909-13
	Nelumbo nucifera	NC_025339.1	KF009944	CPBOL2166-11, CPBOL2168-11, GBVI1013-11, GBVI1015-11, GBVI1017-11, GBVP8284-15, GBVR2913-13, GBVT2414-13, GBVT2415-13, GBVT2416-13, GBVT2417-13, GBVU509-13, GBVX1388-13, GBVX1911-13, GBVX7424-15, GBVX7882-15	CPBOL2168-11, GBVI1014-11, GBVI1016-11, GBVI1018-11, GBVR2913-13, GBVT2575-13, GBVT2576-13, GBVT2577-13, GBVT2578-13, GBVU509-13, GBVX1388-13, GBVX1911-13, GBVX7424-15, GBVX7882-15, GENG797-14
67	*Nicotiana sylvestris*	NC_007500.1	AB237912	GBVK4272-11, GBVK4273-11, GBVR124-13, GBVX1811-13	GBVK4274-11, GBVR4274-11, GBVR124-13, GBVX1811-13, GBVX6650-15
	Nicotiana tabacum	NC_001879.2	-	GBVK4277-11, GBVT1393-13, GBVX1794-13, GBVX2025-13, GBVX6357-15	GBVK4278-11, GBVT1451-13, GBVW2970-13, GBVX1202-13, GBVX1794-13, GBVX2025-13, GBVX6651-15
68	*Nymphaea alba*	NC_006050.1	AJ627251	GBVI1050-11, GBVR1769-13, GBVS1378-13, GBVX1805-13, POWNA1754-12, POWNA1746-12, POWNA1910-12, POWNB040-10	GBVI1051-11, GBVP1965-14, GBVR1769-13, GBVS956-13, GBVX1805-13, POWNA1745-12, POWNA1910-12, POWNB040-10
	Nymphaea mexicana	NC_024542.1	KF753633	GBVX2780-15	GBVX2780-15
69	*Oenothera argillicola*	NC_010358.1	EU262887	GBVR2708-15, GBVX1867-13	GBVR2708-13, GBVX1867-13
	Oenothera elata subsp. hookeri	NC_002693.2	-	GBVR1714-13, GBVX1796-13	GBVR1714-13, GBVX1796-13
70	*Olea europaea subsp. europaea*	NC_015401.1	FN997650	GBVK2600-11, GBVK2601-11, GBVK2602-11, GBVK2603-11, GBVK2604-11, GBVK2605-11, GBVK2606-11, GBVK2607-11, GBVK2608-11, GBVK2609-11, GBVR3395-13, GBVR3968-13, GBVR3969-13, GBVS1572-13, GBVX1906-13	GBVR3395-13, GBVR3968-13, GBVR3969-13, GBVS1572-13, GBVX1906-13
	Olea woodiana subsp. woodiana	NC_015608.1	FN998901	GBVK2631-11, GBVR3971-13, GBVX1910-13	GBVR3971-13, GBVX1910-13

71	*Orobanche californica*	NC_025651.1	HG515539	GBVP6268-15, GBVP7273-15	GBVK3089-11, GBVP6268-15, GBVP7273-15
	Orobanche crenata	NC_024845.1	HG515537	GBVK3101-11, GBVP5951-15, GBVP7070-15	GBVK3102-11, GBVK3103-11, GBVK3104-11, GBVK3105-11, GBVK3106-11, GBVK3107-11, GBVK3108-11
72	*Oryza australiensis*	NC_024608.1	KJ830774	GBVD4224-11, GBVR4559-13, GBVX6566-15	GBVR4559-13, GBVX6566-15
	Oryza rufipogon	NC_017835.1	JN005832	GBVD4250-11, GBVD4251-11, GBVP8430-15, GBVP8431-15, GBVP8432-15, GBVP8433-15, GBVP8434-15, GBVP8435-15, GBVP8436-15, GBVP8437-15, GBVP8438-15, GBVP8439-15, GBVP8440-15, GBVT700-13, GBVT701-13, GBVX1952-13, GBVX2105-15, GBVX2106-15, GBVX2107-15, GBVX2108-15, GBVX2109-15, GBVX2110-15, GBVX2111-15, GBVX2112-15, GBVX2113-15, GBVX2114-15, GBVX2115-15, GBVX2116-15, GBVX2117-15, GBVX2118-15, GBVX2119-15, GBVX2120-15, GBVX2121-15, GBVX2641-15, GBVX2642-15, GBVX2643-15, GBVX2644-15, GBVX2645-15, GBVX2646-15, GBVX2647-15, GBVX2648-15, GBVX7407-15	GBVT700-13, GBVT701-13, GBVX1952-13, GBVX2078-15, GBVX2079-15, GBVX2080-15, GBVX2081-15, GBVX2082-15, GBVX2122-15, GBVX2123-15, GBVX2124-15, GBVX2125-15, GBVX2126-15, GBVX2127-15, GBVX2128-15, GBVX2129-15, GBVX2130-15, GBVX2131-15, GBVX2132-15, GBVX2133-15, GBVX2134-15, GBVX2135-15, GBVX2136-15, GBVX2137-15, GBVX2138-15, GBVX2139-15, GBVX2140-15, GBVX2141-15, GBVX2142-15, GBVX2143-15, GBVX2144-15, GBVX2145-15, GBVX2146-15, GBVX2147-15, GBVX2148-15, GBVX2715-15, GBVX2716-15, GBVX2717-15, GBVX2718-15
73	*Pachycladon cheesemanii*	NC_021102.1	JQ806762	GBVU3860-13, GBVX1996-13	GBVU3860-13, GBVX1996-13
	Pachycladon enysii	NC_018565.1	JX205495	GBVW1153-13, GBVX1965-13	GBVE3384-11, GBVW1153-13, GBVX1965-13
74	*Panax ginseng*	NC_006290.1	AY582139	GBVE1679-11, GBVE1680-11, GBVE1681-11, GBVE1683-11, GBVE1685-11, GBVE1686-11, GBVR056-13, GBVR2216-13, GBVW3847-13, GBVX1807-13, GBVX3234-15, GBVX3235-15, GBVX5223-15, GBVX5363-15, GBVX7877-15	GBVE1682-11, GBVE1684-11, GBVE1687-11, GBVR2216-13, GBVT388-13, GBVT389-13, GBVX1807-13, GBVX3256-15, GBVX3257-15, GBVX5223-15
	Panax notoginseng	NC_026447.1	KJ566590	GBVE1794-11, GBVE1795-11, GBVE1796-11, GBVE1798-11, GBVE1799-11, GBVE1800-11, GBVE1801-11, GBVR059-13, GBVW2685-13, GBVW3848-13, GBVW3849-13, GBVW3850-13, GBVW3851-13, GBVX3240-15, GBVX3241-15, GBVX3241-15, GBVX3242-15, GBVX6136-15	GBVE1797-11, GBVE1802-11, GBVE1803-11, GBVT390-13, GBVX3262-15, GBVX3263-15, GBVX3264-15, GBVX6136-15
75	*Paphiopedilum armeniacum*	NC_026779.1	KJ566307	GBVB4880-11	
	Paphiopedilum niveum	NC_026776.1	KJ524105	GBVU4146-13	
76	*Pariana campestris*	NC_027491.1	KP319244		
	Pariana radiciflora	NC_026972.1	KJ871004	GBVD4370-11	GBVD4371-11
77	*Pelargonium alternans*	NC_023261.1	KF240617	GBVU1273-13	GBVU1330-13
	Pelargonium x hortorum	NC_008454.1	DQ897681	GBVG1833-11	GBVG001-11, GBVG002-11, GBVG1834-11, GBVG1835-11, GBVP2937-14
78	*Phalaenopsis aphrodite subsp. formosana*	NC_007499.1	AY916449	GBVR2310-13, GBVX1810-13	GBVR2310-13, GBVX1810-13
	Phalaenopsis equestris	NC_017609.1	JF719062	GBVC092-11, GBVS4524-13, GBVX1951-13	GBVC093-11, GBVS4524-13, GBVX1951-13
79	*Pharus lappulaceus*	NC_023245.1	KC311467	GBVP2889-14	GBVY3240-14
	Pharus latifolius	NC_021372.1	JN032131	GBVD4409-11, GBVT766-13, GBVX1999-13	GBVD4410-11, GBVT766-13, GBVW3731-13, GBVW3732-13, GBVX1999-13
80	*Phyllostachys nigra var. henonis*	NC_015826.1	HQ154129	GBVS2021-13, GBVX1917-13	GBVS2021-13, GBVS3306-13, GBVX1917-13
	Phyllostachys	NC_016699.1	JN415113	GBVT2655-13, GBVX1936-13	GBVT2655-13, GBVX1936-13

	propinqua				
81	*Picea abies*	NC_021456.1	HF937082	GBVL4636-11, GBVL4637-11, GBVL4645-11, GBVL4647-11, GBVQ038-13, GBVQ576-13, GBVQ618-13, GBVQ838-13, GBVQ1018-13, GBVQ2646-13, GBVQ2647-13, GBVQ2648-13, GBVQ4601-13	GBVL4635-11, GBVL4638-11, GBVL4639-11, GBVL4640-11, GBVL4641-11, GBVL4642-11, GBVL4643-11, GBVL4644-11, GBVL4646-11, GBVL4648-11, GBVQ490-13, GBVQ828-13, GBVQ1018-13, GBVQ2770-13, GBVQ2771-13, GBVQ2772-13, GBVQ4601-13
	Picea morrisonicola	NC_016069.1	AB480556	GBVL4801-11, GBVL4803-11, GBVL4805-11, GBVL4806-11, GBVQ596-13	GBVL4802-11, GBVL4804-11, GBVL4807-11, GBVL4808-11
82	*Pinus monophylla*	NC_011158.4	EU998745	GBVM305-11, GBVM307-11, GBVM309-11, GBVM3011-11, GBVQ635-13, GBVQ3567-13, GBVQ3568-13, GBVQ3569-13, GBVQ4553-13	GBVM306-11, GBVM308-11, GBVM310-11, GBVQ635-13, GBVQ4553-13
	Pinus thunbergii	NC_001631.1	D17510	CPBOL3065-11, CPBOL3067-11, CPBOL3069-11, GBVM575-11, GBVQ666-13, GBVQ1287-13, GBVQ2676-13, GBVQ2677-13, GBVQ4520-13	CPBOL3065-11, CPBOL3067-11, CPBOL3069-11, GBVM576-11, GBVQ666-13, GBVQ1295-13, GBVQ2800-13, GBVQ2801-13, GBVQ4520-13
83	*Podocarpus lambertii*	NC_023805.1	KJ010812	GBVM908-11	GBVM909-11, GBVQ1670-13, GBVY3455-14, GBVY3456-14, GBVY3457-14
	Podocarpus totara	NC_020361.1	KC306742	GBVM994-11, GBVM995-11, GBVQ4217-13, GBVQ4592-13	GBVM996-6, GBVM997-6, GBVM998-6, GBVM999-6, GBVQ1708-13, GBVQ1708-13, GBVQ4217-13, GBVQ4592-13, GBVY3542-14, GBVY3543-14, GBVY3544-14
84	*Populus euphratica*	NC_024747.1	KJ624919	GBVX5400-15, GBVX6348-15, GBVX7857-15, HOSAM117-11	GBVJ4753-11, GBVJ4754-11, GBVX6348-15, GBVX7857-15, HOSAM117-11
	Populus tremula	NC_027425.1	KP861984	GBVJ4777-11, GBVJ4781-11, GBVJ4782-11, GBVJ4783-11, GBVJ4784-11, GBVJ4785-11, GBVJ4786-11, GBVJ4787-11, GBVJ4788-11, GBVJ4789-11, GBVS1398-13, GBVX5414-15, POWNA552-10, POWNA2037-12, POWNA3038-12, POWNB075-10, POWNB158-10	GBVJ4778-11, GBVJ4779-11, GBVJ4780-11, GBVJ4790-11, GBVS992-13, POWNA552-10, POWNA2037-12, POWNA3083-12, POWNB075-10, POWNB158-10
85	*Prunus padus*	NC_026982.1	KP760072	GBVS2282-13, GBVS2283-13, POWNA569-10, POWNA2161-12, POWNA2790-12	GBVJ2570-11, GBVS2540-13, GBVS2541-13, POWNA569-10, POWNA2161-12, POWNA2789-12, POWNA2790-12
	Prunus persica	NC_014697.1	HQ336405	CPBOL3404-11, CPBOL3405-11, CPBOL3406-11, CPBOL3407-11, GBVJ2574-11, GBVJ2575-11, GBVJ2579-11, GBVS1216-13, GBVS2289-13, GBVS2290-13, GBVS2291-13, GBVS2292-13, GBVS2293-13, GBVS2294-13, GBVS2295-13, GBVS2760-13, GBVX1899-13, KNPA1051-09, ZPLPP049-13	CPBOL3404-11, CPBOL3405-11, CPBOL3406-11, CPBOL3407-11, GBVJ2576-11, GBVJ2577-11, GBVJ2578-11, GBVJ2580-11, GBVJ2581-11, GBVS1003-13, GBVS2544-13, GBVS2545-13, GBVS2546-13, GBVS2547-13, GBVS2548-13, GBVS2549-13, GBVS2550-13, GBVS2560-13, GBVT1461-13, GBVX1899-13, KNPA1051-09
86	*Pyrus pyrifolia*	NC_015996.1	AP012207	GBVJ2661-11, GBVR1903-13, GBVU696-13, GBVX1926-13	GBVJ2662-11, GBVR1903-13, GBVU867-13, GBVX1926-13
	Pyrus spinosa	NC_023130.1	HG737342	GBVP2776-14	GBVP2776-14
87	*Quercus aliena*	NC_026790.1	KP301144	GBVG9085-11, GBVG9086-11, GBVG9087-11, GBVG9088-11, GBVG9089-11	GBVG090-11
	Quercus rubra	NC_020152.1	JX970937	GBVG9356-11, GBVG9359-11, GBVG9360-11, GBVG9361-11, GBVG9362-11, GBVG9366-11, GBVG9367-11, GBVG9368-11, GBVS271-13, GBVW3648-13, GBVX1979-13, VEMSH084-13, WABLK112-13, WABLK194-13	GBVG355-11, GBVG357-11, GBVG358-11, GBVG363-11, GBVG364-11, GBVG365-11, GBVW3648-13, GBVX1979-13, KSR494-08, VEMSH084-13, WABLK112-13, WABLK194-13
88	*Salicornia bigelovii*	NC_027226.1	KJ629117		
	Salicornia brachiata	NC_027224.1	KJ629115		
89	*Salix interior*	NC_024681.1	KJ742926	GBVX6520-15, GBVX7837-15, SALIX112-08, SALIX183-08, SALIX254-08, SALIX265-08, SALIX317-08, SALIX331-08, SALIX336-08, SALIX337-08, SALIX525-08	GBVJ4931-11, GBVX6520-15, GBVX7837-15, SALIX112-08, SALIX183-08, SALIX218-08, SALIX254-08, SALIX265-08, SALIX317-08, SALIX331-08, SALIX336-08, SALIX337-08, SALIX338-08, SALIX525-08, VEMSH070-13
	Salix purpurea	NC_026722.1	KP019639	GBVJ4991-11, GBVJ4992-11, GBVJ4993-11, POWNA644-10, POWNA1701-12, POWNA1702-12	GBVJ4989-11, GBVJ4990-11, GBVJ4994-11, POWNA644-10, POWNA1701-12, POWNA1702-12
90	*Sartidia dewinteri*	NC_027147.1	KJ819550		
	Sartidia perrieri	NC_027146.1	KJ819549		
91	*Sedum sarmentosum*	NC_023085.1	JX427551	GBVJ1726-11	GBVJ1727-11,VGBVJ1728-11
	Sedum takesimense	NC_026065.1	KF954541	GBVX5421-15, GBVX7901-15	GBVX5421-15, GBVX7901-15

92	*Silene latifolia*	NC_016730.1	JF715055	GBVF311-11, GBVF312-11, GBVF313-11, GBVF314-11, GBVF315-11, GBVF316-11, GBVF317-11, GBVF318-11, GBVF319-11, GBVF320-11, GBVF321-11, GBVF322-11, GBVF324-11, GBVF325-11, GBVF326-11, GBVF327-11, GBVF328-11, GBVF329-11, GBVF330-11, GBVF331-11, GBVF332-11, GBVF333-11, GBVF334-11, GBVF335-11, GBVF336-11, GBVF337-11, GBVF338-11, GBVF339-11, GBVF340-11, GBVF341-11, GBVF342-11, GBVF343-11, GBVF344-11, GBVF345-11, GBVF346-11, GBVF347-11, GBVF348-11, GBVF349-11, GBVF350-11, GBVF351-11, GBVF352-11, GBVF353-11, GBVF354-11, GBVF355-11, GBVF356-11, GBVF357-11, GBVF358-11, GBVF359-11, GBVF360-11, GBVF361-11, GBVF362-11, GBVF363-11, GBVF364-11, GBVF365-11, GBVF366-11, GBVF367-11, GBVF368-11, GBVF369-11, GBVF370-11, GBVF371-11, GBVF372-11, GBVF373-11, GBVF374-11, GBVF375-11, GBVF376-11, GBVF377-11, GBVF378-11, GBVF379-11, GBVF380-11, GBVF381-11, GBVF382-11, GBVF383-11, GBVF384-11, GBVF385-11, GBVF386-11, GBVF387-11, GBVF388-11, GBVF389-11, GBVF390-11, GBVF391-11, GBVF392-11, GBVF393-11, GBVF394-11, GBVF395-11, GBVF396-11, GBVF397-11, GBVF398-11, GBVF399-11, GBVF451-11, GBVF452-11, GBVF453-11, GBVR098-13, GBVS4481-13, GBVW2416-13, GBVX1944-13, KSR049-07, MKPCH562-09, POWNA1709-12, POWNA681-10, POWNB104-10, VEMSH201-13	GBVF310-11, GBVF323-11, GBVF400-11, GBVF401-11, GBVF402-11, GBVF403-11, GBVF404-11, GBVF405-11, GBVF406-11, GBVF407-11, GBVF408-11, GBVF409-11, GBVF410-11, GBVF411-11, GBVF412-11, GBVF413-11, GBVF414-11, GBVF415-11, GBVF416-11, GBVF417-11, GBVF418-11, GBVF419-11, GBVF420-11, GBVF421-11, GBVF422-11, GBVF423-11, GBVF424-11, GBVF425-11, GBVF426-11, GBVF427-11, GBVF428-11, GBVF429-11, GBVF430-11, GBVF431-11, GBVF432-11, GBVF433-11, GBVF434-11, GBVF435-11, GBVF436-11, GBVF437-11, GBVF438-11, GBVF439-11, GBVF440-11, GBVF441-11, GBVF442-11, GBVF443-11, GBVF444-11, GBVF445-11, GBVF446-11, GBVF447-11, GBVF448-11, GBVF449-11, GBVF450-11, GBVS4481-13, GBVW2425-13, GBVX1944-13, KSR049-07, MKPCH562-09, POWNA1709-12, POWNA1710-12, POWNA681-10, POWNB104-10, RRPLA033-15, VEMSH201-13
	Silene vulgaris	NC_016727.1	JF715057	GBVF545-11, GBVF546-11, GBVF547-11, GBVF548-11, GBVF549-11, GBVF550-11, GBVF551-11, GBVF552-11, GBVF553-11, GBVF554-11, GBVF556-11, GBVF557-11, GBVF558-11, GBVS4483-13, GBVX1941-13	GBVF541-11, GBVF542-11, GBVF543-11, GBVF544-11, GBVF555-11, GBVS4483-13, GBVX1941-13, POWNA1714-12, POWNA685-10, VEMSH090-13
93	*Solanum bulbocastanum*	NC_007943.1	DQ347958	GBVK4514-11, GBVR2414-13, GBVX1816-13	GBVR2414-13, GBVX1816-13
	Solanum tuberosum	NC_008096.2	DQ386163	GBVK4655-11, GBVK4656-11, GBVK4657-11, GBVK4658-11, GBVR2398-13, GBVR2420-13, GBVS4535-13, GBVS4536-13, GBVX1820-13, GBVX6354-15	GBVK4659-11, GBVK4660-11, GBVR2398-13, GBVR2420-13, GBVS1603-13, GBVS4535-13, GBVS4536-13, GBVW2980-13, GBVX1820-13, GBVX6360-15
94	*Sorghum bicolor*	NC_008602.1	EF115542	GBVD4728-11, GBVR2598-13, GBVX1836-13	FSSM057-13, FSSM058-13, GBVD4729-11, GBVR2598-13, GBVX1836-13
	Sorghum timorense	NC_023800.1	KF998272		GBVX2100-15
95	*Taiwania cryptomerioides*	NC_016065.1	AP012266	GBVL4447-11, GBVL4448-11, GBVL4449-11, GBVQ513-13, GBVQ1149-13, GBVQ1370-13, GBVQ4581-13	GBVL4450-11, GBVQ513-13, GBVQ1409-13, GBVQ4581-13
	Taiwania flousiana	NC_021441.1	KC427274	GBVL4451-11, GBVQ4242-13, GBVQ4600-13	GBVQ4242-13, GBVQ4600-13
96	*Trifolium repens*	NC_024036.1	KC894706	GBVP048-11, GBVP053-11, GBVP054-11, GBVP055-11, GBVP056-11, GBVS1253-13, PLGE039-13, POWNA767-10, POWNA1728-12, VEMSH191-13, WABLK373-13	CSU029-10, GBVP047-11, GBVP049-11, GBVP050-11, GBVP051-11, GBVP052-11, GBVP057-11, VS1096-13, PLGE039-13, POWNA767-10, POWNA1728-12, POWNA1808-12, POWNA2583-12, RPLA001-15, VEMSH191-13, WABLK373-13
	Trifolium subterraneum	NC_011828.1	EU849487	GBVP071-11, GBVP073-11, GBVR2771-13, GBVX1878-13	GBVP072-11, GBVR2771-13, GBVX1878-13, POWNA771-10, POWNA2759-12
97	*Trillium maculatum*	NC_027738.1	KR780075	GBVC3838-11, GBVP2756-14, GBVP5612-15	GBVP5754-15
	Trillium tschonoskii	NC_027739.1	KR780076	GBVC3860-11, GBVT2726-13	GBVT2816-13
98	*Triticum macha*	NC_025955.1	LC005978	GBVD4883-11, GBVX7728-15, GBVX7900-15	GBVX7728-15, GBVX4851-15
	Triticum timopheevii	NC_024764.1	KJ614410	GBVD4908-11, GBVD4909-11, GBVD4910-11, GBVD4911-11, GBVX4851-15, GBVX6333-15, GBVX6334-15, GBVX6335-15, GBVX6336-15, GBVX7858-15	GBVX4851-15, GBVX6333-15, GBVX6334-15, GBVX6335-15, GBVX6336-15, GBVX7795-15, GBVX7796-15, GBVX7858-15
99	*Utricularia gibba*	NC_021449.1	KC997777	GBVX1387-13	GBVK2243-11, GBVX1387-13
	Utricularia macrorhiza	NC_025653.1	HG803177	GBVK2251-11	GBVX4856-15, GBVX7888-15
100	*Vigna angularis*	NC_021091.1	AP012598	GBVP1134-11, GBVP1135-11, GBVP1136-11, GBVP1137-11, GBVP1138-11, GBVP1139-11, GBVP1140-11, GBVR1904-13, GBVX1994-13	GBVP2095-14, GBVP2096-14, GBVP2097-14, GBVP2098-14, GBVP2099-14, GBVP2100-14, GBVR1904-13, GBVX1994-13
	Vigna unguiculata	NC_018051.1	JQ755301	GBVP1193-11, GBVP1194-11, GBVP1195-11, GBVP1197-11, GBVP1198-11, GBVP1199-11, GBVP1200-11, GBVP1201-11, GBVP1202-11, GBVP1209-11, GBVP1210-11, GBVP1212-11, GBVT3682-13, GBVU3119-13, GBVX1954-13	GBVP1196-11, GBVP1204-11, GBVP1205-11, GBVP1206-11, GBVP1207-11, GBVP1208-11, GBVP1211-11, GBVT3682-13, GBVU3119-13, GBVX1954-13
101	*Vitis rotundifolia*	NC_023790.1	KF976463		CSU014-10, GBVJ1333-11, GBVJ1334-11
	Vitis vinifera	NC_007957.1	DQ424856	GBVJ1335-11, GBVR2422-13, GBVS1444-13, GBVT1406, GBVX1818-13	GBVJ1336-11, GBVR2422-13, GBVS4411-13, GBVS1117-13, GBVT1473-13, GBVW1481-13, GBVX1818-13, GBVX1879-13, HIDNA046-14

Supplementary Table S3. Records used to compile the Comprehensive Reference Barcode database. List of 735 species for which sequence data of *mat*K and *rbc*L were downloaded from complete chloroplast genomes from NCBI forming the basis of the artificial Comprehensive Reference Barcode (CRB) database.

Species	Accession numbers	
Abies koreana	NC_026892.1	KP742350
Acacia ligulata	NC_026134.1	LN555649
Acidosasa purpurea	NC_015820.1	HQ337793
Acorus americanus	NC_010093.1	EU273602
Acorus calamus	NC_007407.1	AJ879453
Acorus gramineus	NC_026299.1	KP099646
Actinidia chinensis	NC_026690.1	KP297242
Actinidia deliciosa	NC_026691.1	KP297244
Adenophora remotiflora	NC_026999.1	KP889213
Adiantum capillus-veneris	NC_004766.1	AY178864
Aegilops bicornis	NC_024831.1	KJ614418
Aegilops cylindrica	NC_023096.1	KF534489
Aegilops geniculata	NC_023097.1	KF534490
Aegilops kotschyi	NC_024832.1	KJ614420
Aegilops longissima	NC_024830.1	KJ614416
Aegilops searsii	NC_024815.1	KJ614414
Aegilops sharonensis	NC_024816.1	KJ614419
Aegilops speltoides	NC_022135.1	JQ740834
Aegilops tauschii	NC_022133.1	JQ754651
Aethionema cordifolium	NC_009265.1	AP009366
Aethionema grandiflorum	NC_009266.1	AP009367
Agathis dammara	NC_023119.1	AB830884
Ageratina adenophora	NC_015621.1	JF826503
Agrostemma githago	NC_023357.1	KF527884
Agrostis stolonifera	NC_008591.1	EF115543
Ajuga reptans	NC_023102.1	KF709391
Allium cepa	NC_024813.1	KF728080
Allosyncarpia ternata	NC_022413.1	KC180806
Alsophila spinulosa	NC_012818.1	FJ556581
Amborella trichopoda	NC_005086.1	AJ506156
Amentotaxus argotaenia	NC_027581.1	KR780582
Amentotaxus formosana	NC_024945.1	AP014574
Ammophila breviligulata	NC_027465.1	KM974730
Ampelocalamus calcareus	NC_024731.1	KJ496369
Ampelodesmos mauritanicus	NC_027466.1	KM974731
Ananas comosus	NC_026220.1	AP014632
Andrographis paniculata	NC_022451.2	KF150644
Aneura mirabilis	NC_010359.1	EU043314
Angiopteris angustifolia	NC_026300.1	KP099647
Angiopteris evecta	NC_008829.1	DQ821119
Angophora costata	NC_022412.1	KC180805
Angophora floribunda	NC_022411.1	KC180804
Anomochloa marantoidea	NC_014062.1	GQ329703
Anthoceros angustus	NC_004543.1	AB086179
Anthoxanthum nitens	NC_027475.1	KM974740
Anthoxanthum odoratum	NC_027467.1	KM974732
Anthriscus cerefolium	NC_015113.1	GU456628
Apios americana	NC_025909.1	-
Arabidopsis thaliana	NC_000932.1	AP000423
Arabis alpina	NC_023367.1	HF934132
Arabis hirsuta JO23	NC_009268.1	AP009369
Arachis hypogaea	NC_026676.1	-
Aralia undulata	NC_022810.1	KC456163
Araucaria heterophylla	NC_026450.1	KM067155
Ardisia polysticta	NC_021121.1	KC465962
Aristida purpurea	NC_025228.1	KJ920224
Artemisia frigida	NC_020607.1	JX293720
Artemisia montana	NC_025910.1	KF887960
Arundinaria appalachiana	NC_023934.1	KC817462
Arundinaria fargesii	NC_024712.1	JX513413
Arundinaria gigantea	NC_020341.1	JX235347
Arundinaria tecta	NC_023935.1	KC817463
Asclepias nivea	NC_022431.1	KF539844
Asclepias syriaca	NC_022432.1	KF386166
Aster spathulifolius	NC_027434.1	KF279514
Atropa belladonna Ab5p(kan)	NC_004561.1	AJ316582
Avena sativa	NC_027468.1	KM974733
Azadirachta indica	NC_023792.1	KF986530
Bambusa arnhemica	NC_026958.1	KJ870989
Bambusa bambos	NC_026957.1	KJ870988
Bambusa emeiensis	NC_015830.1	HQ337797
Bambusa multiplex	NC_024668.1	KJ722536
Bambusa oldhamii	NC_012927.1	FJ970915

Barbarea verna	NC_009269.1	AP009370
Berberis bealei	NC_022457.1	KF176554
Bismarckia nobilis	NC_020366.1	JX088664
Boea hygrometrica	NC_016468.1	JN107811
Bomarea edulis	NC_025306.1	KM233641
Bowenia serrulata	NC_026036.1	JX402774
Brachyelytrum aristosum	NC_027470.1	KM974735
Brachypodium distachyon	NC_011032.1	EU325680
Brassaiopsis hainla	NC_022811.1	KC456164
Brassica napus ZY036	NC_016734.1	GQ861354
Brassica rapa	NC_015139.1	DQ231548
Briza maxima	NC_027471.1	KM974736
Bromus vulgaris	NC_027472.1	KM974737
Buergersiochloa bambusoides	NC_026968.1	KJ871000
Buxus microphylla	NC_009599.1	EF380351
Calamus caryotoides	NC_020365.1	JX088663
Calanthe triplicata	NC_024544.1	KF753635
Callitropsis nootkatensis	NC_026295.1	KP099642
Callitropsis vietnamensis	NC_026298.1	KP099645
Calocedrus formosana	NC_023121.1	AB831010
Calycanthus floridus var. glaucus	NC_004993.1	AJ428413
Camellia crapnelliana	NC_024541.1	KF753632
Camellia cuspidata	NC_022459.1	KF156833
Camellia danzaiensis	NC_022460.1	KF156834
Camellia grandibracteata	NC_024659.1	KJ806274
Camellia impressinervis	NC_022461.1	KF156835
Camellia leptophylla	NC_024660.1	KJ806275
Camellia oleifera	NC_023084.1	JQ975031
Camellia petelotii	NC_024661.1	KJ806276
Camellia pitardii	NC_022462.1	KF156837
Camellia pubicosta	NC_024662.1	KJ806277
Camellia reticulata	NC_024663.1	KJ806278
Camellia sinensis	NC_020019.1	KC143082
Camellia taliensis	NC_022264.1	KF156839
Camellia yunnanensis	NC_022463.1	KF156838
Campanula takesimana	NC_026203.1	KP006497
Campynema lineare	NC_026785.1	KP462881
Cannabis sativa	NC_026562.1	KP274871
Cannabis sativa	NC_027223.1	KR363961
Capsella bursa-pastoris JO22	NC_009270.1	AP009371
Capsella rubella	NC_027693.1	KR029093
Capsicum annuum	NC_018552.1	JX270811
Capsicum lycianthoides	NC_026551.1	KP274856
Cardamine impatiens	NC_026445.1	KJ136821
Cardamine resedifolia	NC_026446.1	KJ136822
Carex siderosticta	NC_027250.1	KP751906
Carica papaya	NC_010323.1	EU431223
Carludovica palmata	NC_026786.1	KP462882
Carnegiea gigantea	NC_027618.1	KT164771
Castanea mollissima	NC_014674.1	HQ336406
Castanopsis echinocarpa	NC_023801.1	KJ001129
Catharanthus roseus	NC_021423.1	KC561139
Cathaya argyrophylla	NC_014589.1	AB547400
Cattleya crispata	NC_026568.1	KP168671
Cedrus deodara	NC_014575.1	AB480043
Cenchrus americanus	NC_024171.1	KJ490012
Centaurea diffusa	NC_024286.1	KJ690264
Centotheca lappacea	NC_025229.1	KJ920225
Cephalotaxus oliveri	NC_021110.1	KC136217
Cephalotaxus wilsoniana	NC_016063.1	AP012265
Ceratonia siliqua	NC_026678.1	-
Ceratophyllum demersum	NC_009962.1	EF614270
Ceratozamia hildae	NC_026037.1	JX407108
Chikusichloa aquatica	NC_027184.1	KR078265
Chimonocalamus longiusculus	NC_024714.1	JX513415
Chionochloa macra	NC_025230.1	KJ920227
Chloranthus japonicus	NC_026565.1	KP256024
Chloranthus spicatus	NC_009598.1	EF380352
Chrysanthemum indicum	NC_020320.1	JN867589
Chrysanthemum x morifolium	NC_020092.1	JQ362483
Chrysobalanus icaco	NC_024061.1	KJ414480
Chusquea circinata	NC_027490.1	KP319241
Chusquea liebmannii	NC_026969.1	KJ871001
Chusquea spectabilis	NC_026959.1	KJ870990
Cicer arietinum	NC_011163.1	EU835853
Cistanche deserticola	NC_021111.1	KC128846
Cistanche phelypaea	NC_025642.1	HG515538
Citrus aurantiifolia	NC_024929.1	KJ865401
Citrus sinensis	NC_008334.1	DQ864733
Cocos nucifera	NC_022417.1	KF285453
Coffea arabica	NC_008535.1	EF044213

Coix lacryma-jobi	NC_013273.1	FJ261955
Coleataenia prionitis	NC_025231.1	KJ920228
Colocasia esculenta	NC_016753.1	JN105689
Corallorhiza bulbosa	NC_025659.1	KM390013
Corallorhiza macrantha	NC_025660.1	KM390017
Corallorhiza mertensiana	NC_025661.1	KM390018
Corallorhiza odontorhiza	NC_025664.1	KM390021
Corallorhiza trifida	NC_025662.1	KM390019
Corallorhiza wisteriana	NC_025663.1	KM390020
Corymbia eximia	NC_022409.1	KC180802
Corymbia gummifera	NC_022407.1	KC180800
Corymbia maculata	NC_022408.1	KC180801
Corymbia tessellaris	NC_022410.1	KC180803
Corynocarpus laevigata	NC_014807.1	HQ207704
Couepia guianensis	NC_024063.1	KJ414482
Crucihimalaya wallichii JS5	NC_009271.1	AP009372
Cryptomeria japonica	NC_010548.1	AP009377
Cucumis hystrix	NC_023544.1	KF957866
Cucumis melo	NC_015983.1	JF412791
Cucumis sativus	NC_007144.1	AJ970307
Cunninghamia lanceolata	NC_021437.1	KC427270
Cupressus sempervirens	NC_026296.1	KP099643
Curcuma roscoeana	NC_022928.1	KF601574
Cuscuta exaltata	NC_009963.1	EU189132
Cuscuta reflexa	NC_009766.1	AM711640
Cycas revoluta	NC_020319.1	JN867588
Cycas taitungensis	NC_009618.1	AP009339
Cymbidium aloifolium	NC_021429.1	KC876122
Cymbidium faberi	NC_027743.1	KR919606
Cymbidium mannii	NC_021433.1	KC876129
Cymbidium sinense	NC_021430.1	KC876123
Cymbidium tortisepalum	NC_021431.1	KC876124
Cymbidium tracyanum	NC_021432.1	KC876127
Cynara humilis	NC_027113.1	KP299292
Cypripedium formosanum	NC_026772.1	KJ501998
Cypripedium japonicum	NC_027227.1	KJ625630
Cypripedium macranthos	NC_024421.1	KF925434
Dactylis glomerata	NC_027473.1	KM974738
Danthonia californica	NC_025232.1	KJ920229
Dasypogon bromeliifolius	NC_020367.1	JX088665
Datura stramonium	NC_018117.1	JN654342
Daucus carota	NC_008325.1	DQ898156
Dendrobium catenatum	NC_024019.1	KC771275
Dendrobium strongylanthum	NC_027691.1	KR673323
Dendrocalamus latiflorus	NC_013088.1	FJ970916
Dendropanax dentiger	NC_026546.1	KP271241
Dendropanax morbifer	NC_027607.1	KR136270
Diandrolyra sp. Clark 1301	NC_026960.1	KJ870991
Diarrhena obovata	NC_027474.1	KM974739
Dieffenbachia seguine	NC_027272.1	KR262889
Digitaria exilis	NC_024176.1	KJ513091
Dioon spinulosum	NC_027512.1	LC049070
Dioscorea elephantipes	NC_009601.1	EF380353
Dioscorea rotundata	NC_024170.1	KJ490011
Dioscorea zingiberensis	NC_027090.1	KP899622
Diplopterygium glaucum	NC_024158.1	KF225594
Draba nemorosa JO21	NC_009272.1	AP009373
Drimys granadensis	NC_008456.1	DQ887676
Dunalia brachyacantha	NC_026906.1	KP308151
Dunalia obovata	NC_026563.1	KP280057
Dunalia solanacea	NC_027099.1	KP998157
Echinochloa oryzicola STB03	NC_024643.2	KJ000048
Echites umbellatus	NC_025655.1	KJ953904
Elaeis guineensis	NC_017602.1	JF274081
Eleutherococcus senticosus	NC_016430.1	JN637765
Elleanthus sodiroi	NC_027266.1	KR260986
Elodea canadensis	NC_018541.1	JQ310743
Elytrophorus spicatus	NC_025233.1	KJ920230
Encephalartos lehmannii	NC_027514.1	LC049336
Ephedra equisetina	NC_011954.1	AP010819
Equisetum arvense	NC_014699.1	GU191334
Equisetum hyemale	NC_020146.1	KC117177
Eriachne stipacea	NC_025234.1	KJ920231
Erodium absinthoides	NC_026847.1	KJ523886
Erodium carvifolium	NC_015083.1	HQ713469
Erodium chrysanthum	NC_027065.1	KJ701602
Erodium crassifolium	NC_025906.1	KF977221
Erodium gruinum	NC_025907.1	KF804069
Erodium texanum	NC_014569.1	HM125536
Erodium trifolium	NC_024635.1	KF441758
Erycina pusilla	NC_018114.1	JF746994

Eucalyptus aromaphloia	NC_022396.1	KC180789
Eucalyptus baxteri	NC_022382.1	KC180773
Eucalyptus camaldulensis	NC_022398.1	KC180791
Eucalyptus cladocalyx	NC_022394.1	KC180786
Eucalyptus cloeziana	NC_022388.1	KC180779
Eucalyptus curtisii	NC_022391.1	KC180782
Eucalyptus deglupta	NC_022399.1	KC180792
Eucalyptus delegatensis	NC_022380.1	KC180771
Eucalyptus diversicolor	NC_022402.1	KC180795
Eucalyptus diversifolia	NC_022383.1	KC180774
Eucalyptus elata	NC_022385.1	KC180776
Eucalyptus erythrocorys	NC_022406.1	KC180799
Eucalyptus globulus subsp. globulus	NC_008115.1	AY780259
Eucalyptus grandis	NC_014570.1	HM347959
Eucalyptus guilfoylei	NC_022405.1	KC180798
Eucalyptus marginata	NC_022390.1	KC180781
Eucalyptus melliodora	NC_022392.1	KC180784
Eucalyptus microcorys	NC_022404.1	KC180797
Eucalyptus nitens	NC_022395.1	KC180788
Eucalyptus obliqua	NC_022378.1	KC180769
Eucalyptus patens	NC_022389.1	KC180780
Eucalyptus polybractea	NC_022393.1	KC180785
Eucalyptus radiata	NC_022379.1	KC180770
Eucalyptus regnans	NC_022386.1	KC180777
Eucalyptus saligna	NC_022397.1	KC180790
Eucalyptus salmonophloia	NC_022403.1	KC180796
Eucalyptus sieberi	NC_022384.1	KC180775
Eucalyptus spathulata	NC_022400.1	KC180793
Eucalyptus torquata	NC_022401.1	KC180794
Eucalyptus umbra	NC_022387.1	KC180778
Eucalyptus verrucata	NC_022381.1	KC180772
Eugenia uniflora	NC_027744.1	KR867678
Eustrephus latifolius	NC_025305.1	KM233639
Fagopyrum esculentum subsp. ancestrale	NC_010776.1	EU254477
Fagopyrum tataricum Daegwan3-3	NC_027161.1	KM201427
Fargesia nitida	NC_024715.1	JX513416
Fargesia spathacea	NC_024716.1	JX513417
Fargesia yunnanensis	NC_024717.1	JX513418
Fatsia japonica	NC_027685.1	KR021045
Ferrocalamus rimosivaginus	NC_015831.1	HQ337794
Festuca altissima	NC_019648.1	JX871939
Festuca arundinacea	NC_011713.2	FJ466687
Festuca ovina	NC_019649.1	JX871940
Festuca pratensis	NC_019650.1	JX871941
Fragaria chiloensis	NC_019601.1	JN884816
Fragaria iinumae	NC_024258.1	KC507759
Fragaria mandshurica	NC_018767.1	JQ396172
Fragaria vesca subsp. bracteata	NC_018766.1	JQ396171
Fragaria vesca subsp. vesca	NC_015206.1	JF345175
Fragaria virginiana	NC_019602.1	JN884817
Francoa sonchifolia	NC_021101.1	JQ809470
Fritillaria cirrhosa	NC_024728.1	KF769143
Fritillaria hupehensis	NC_024736.1	KF712486
Fritillaria taipaiensis	NC_023247.1	KC543997
Gaoligongshania megalothyrsa	NC_024718.1	JX513419
Gelidocalamus tessellatus	NC_024719.1	JX513420
Gentiana crassicaulis	NC_027442.1	KJ676538
Gentiana straminea	NC_027441.1	KJ657732
Geranium palmatum	NC_014573.1	HM125537
Ginkgo biloba	NC_016986.1	JN867585
Glycine canescens	NC_021647.1	KC893635
Glycine cyrtoloba	NC_021645.1	KC893632
Glycine dolichocarpa	NC_021648.1	KC893636
Glycine falcata	NC_021649.1	KC893637
Glycine max	NC_007942.1	DQ317523
Glycine soja	NC_022868.1	KF611800
Glycine stenophita	NC_021646.1	KC893634
Glycine syndetika	NC_021650.1	KC893638
Glycine tomentella	NC_021636.1	KC893633
Glycyrrhiza glabra	NC_024038.1	KF201590
Gnetum gnemon	NC_026301.1	KP099649
Gnetum montanum	NC_021438.1	KC427271
Gnetum parvifolium	NC_011942.1	AP009569
Goodyera fumata	NC_026773.1	KJ501999
Gossypium anomalum	NC_023213.1	JF317351
Gossypium arboreum	NC_016712.1	HQ325740
Gossypium areysianum	NC_018112.1	JN019795
Gossypium bickii	NC_023214.1	JF317352
Gossypium capitis-viridis	NC_018111.1	JN019794
Gossypium darwinii	NC_016670.1	HQ325741
Gossypium gossypioides	NC_017894.1	HQ901195

Gossypium herbaceum	NC_023215.1	JF317353
Gossypium herbaceum subsp. africanum	NC_016692.1	HQ325742
Gossypium hirsutum	NC_007944.1	DQ345959
Gossypium incanum	NC_018109.1	JN019792
Gossypium longicalyx	NC_023216.1	JF317354
Gossypium mustelinum	NC_016711.1	HQ325743
Gossypium raimondii	NC_016668.1	HQ325744
Gossypium robinsonii	NC_018113.1	JN019791
Gossypium somalense	NC_018110.1	JN019793
Gossypium stocksii	NC_023217.1	JF317355
Gossypium sturtianum	NC_023218.1	JF317356
Gossypium thurberi	NC_015204.1	GU907100
Gossypium tomentosum	NC_016690.1	HQ325745
Gossypium turneri	NC_026835.1	JQ742090
Greslania sp. McPherson 19217	NC_026961.1	KJ870993
Guadua weberbaueri	NC_026991.1	KP793062
Guizotia abyssinica	NC_010601.1	EU549769
Habenaria pantlingiana	NC_026775.1	KJ524104
Haematoxylum brasiletto	NC_026679.1	-
Hakonechloa macra	NC_025235.1	KJ920232
Haloxylon ammodendron	NC_027668.1	KF534478
Haloxylon persicum	NC_027669.1	KF534479
Hanabusaya asiatica	NC_024732.1	KJ477692
Helianthus annuus	NC_007977.1	DQ383815
Helianthus decapetalus	NC_023110.1	KF746356
Helianthus divaricatus	NC_023109.1	KF746352
Helianthus giganteus	NC_023107.1	KF746346
Helianthus grosseserratus	NC_023108.1	KF746350
Helianthus hirsutus	NC_023111.1	KF746359
Helianthus maximiliani	NC_023114.1	KF746380
Helianthus strumosus	NC_023113.1	KF746376
Helianthus tuberosus	NC_023112.1	KF746361
Heliconia collinsiana	NC_020362.1	JX088660
Helictochloa hookeri	NC_027469.1	KM974734
Heloniopsis tubiflora	NC_027159.1	KM078036
Hesperelaea palmeri	NC_025787.1	LN515489
Hesperocyparis glabra	NC_026297.1	KP099644
Hevea brasiliensis	NC_015308.1	HQ285842
Hibiscus syriacus	NC_026909.1	KP688069
Hickelia madagascariensis	NC_026962.1	KJ870994
Hirtella physophora	NC_024066.1	KJ414485
Hirtella racemosa	NC_024060.1	KJ414479
Hordeum jubatum	NC_027476.1	KM974741
Hordeum vulgare subsp. vulgare	NC_008590.1	EF115541
Huperzia lucidula	NC_006861.1	AY660566
Hypseocharis bilobata	NC_023260.1	KF240616
Illicium oligandrum	NC_009600.1	EF380354
Indigofera tinctoria	NC_026680.1	-
Indocalamus longiauritus	NC_015803.1	HQ337795
Indocalamus wilsonii	NC_024720.1	JX513421
Indosasa sinica	NC_024721.1	JX513422
Iochroma loxense	NC_026726.1	KP296185
Iochroma nitidum	NC_026567.1	KP294386
Iochroma stenanthum	NC_026574.1	KP262399
Iochroma tingoanum	NC_027177.1	KP280175
Ipomoea batatas	NC_026703.1	KP212149
Ipomoea purpurea	NC_009808.1	EU118126
Iris gatesii	NC_024936.1	KM014691
Isachne distichophylla	NC_025236.1	KJ920233
Isoetes flaccida	NC_014675.1	GU191333
Jacobaea vulgaris	NC_015543.1	HQ234669
Jasminum nudiflorum	NC_008407.1	DQ673255
Jatropha curcas	NC_012224.1	FJ695500
Juniperus bermudiana	NC_024021.1	KF866297
Juniperus monosperma	NC_024022.1	KF866298
Juniperus scopulorum	NC_024023.1	KF866299
Juniperus virginiana	NC_024024.1	KF866300
Kalopanax septemlobus	NC_022814.1	KC456167
Keteleeria davidiana	NC_011930.1	AP010820
Lactuca sativa	NC_007578.1	AP007232
Larix decidua	NC_016058.1	AB501189
Lathyrus clymenum	NC_027148.1	KJ850235
Lathyrus davidii	NC_027073.1	KJ806192
Lathyrus graminifolius	NC_027074.1	KJ806193
Lathyrus inconspicuus	NC_027149.1	KJ850236
Lathyrus japonicus	NC_027075.1	KJ806194
Lathyrus littoralis	NC_027076.1	KJ806196
Lathyrus ochroleucus	NC_027077.1	KJ806197
Lathyrus odoratus	NC_027150.1	KJ850237
Lathyrus palustris	NC_027078.1	KJ806199
Lathyrus pubescens	NC_027079.1	KJ806200

Lathyrus sativus	NC_014063.1	HM029371
Lathyrus tingitanus	NC_027151.1	KJ850238
Lathyrus venosus	NC_027080.1	KJ806202
Lecomtella madagascariensis	NC_024106.1	HF543599
Leersia tisserantii	NC_016677.1	JN415112
Lemna minor	NC_010109.1	DQ400350
Lens culinaris	NC_027152.1	KJ850239
Lepidium virginicum JO26	NC_009273.1	AP009374
Lepidozamia peroffskyana	NC_027513.1	LC049207
Libidibia coriaria	NC_026677.1	-
Licania alba	NC_024064.1	KJ414483
Licania heteromorpha	NC_024062.1	KJ414481
Licania sprucei	NC_024065.1	KJ414484
Lilium hansonii	NC_027674.1	KM103364
Lilium sp. KHK-2014	NC_027679.1	KM103383
Lilium superbum	NC_026787.1	KP462883
Lilium tsingtauense	NC_027675.1	KM103365
Liquidambar formosana	NC_023092.1	KC588388
Liriodendron tulipifera	NC_008326.1	DQ899947
Lithachne pauciflora	NC_026970.1	KJ871002
Lithocarpus balansae	NC_026577.1	KP299291
Lobularia maritima	NC_009274.1	AP009375
Lolium multiflorum	NC_019651.1	JX871942
Lolium perenne	NC_009950.1	AM777385
Lonicera japonica	NC_026839.1	KJ170923
Lotus japonicus Accession MG-20	NC_002694.1	AP002983
Lupinus albus	NC_026681.1	-
Lupinus luteus	NC_023090.1	KC695666
Luzuriaga radicans	NC_025333.1	KM233640
Lygodium japonicum	NC_022136.1	KC536645
Lysimachia coreana	NC_026197.1	KM819521
Macadamia integrifolia	NC_025288.1	KF862711
Macrozamia mountperriensis	NC_027511.1	LC049069
Magnolia denudata	NC_018357.1	JN227740
Magnolia grandiflora	NC_020318.1	JN867584
Magnolia kwangsiensis	NC_015892.1	HM775382
Magnolia officinalis	NC_020316.1	JN867579
Magnolia officinalis subsp. biloba	NC_020317.1	JN867580
Magnolia tripetala	NC_024027.1	KJ408574
Magnolia yunnanensis	NC_024545.1	KF753638
Manihot esculenta	NC_010433.1	EU117376
Mankyua chejuensis	NC_017006.1	JF343520
Marsilea crenata	NC_022137.1	KC536646
Masdevallia coccinea	NC_026541.1	KP205432
Masdevallia picturata	NC_026777.1	KJ566305
Medicago hybrida	NC_027153.1	KJ850240
Medicago papillosa	NC_027154.1	KJ850241
Megaleranthis saniculifolia	NC_012615.1	FJ597983
Melianthus villosus	NC_023256.1	KF017614
Melica mutica	NC_027477.1	KM974742
Melica subulata	NC_027478.1	KM974743
Metapanax delavayi	NC_022812.1	KC456165
Metasequoia glyptostroboides	NC_027423.1	KR061358
Millettia pinnata	NC_016708.2	JN673818
Monachather paradoxus	NC_025237.1	KJ920235
Monsonia speciosa	NC_014582.1	HM125538
Morus indica	NC_008359.1	DQ226511
Morus mongolica	NC_025772.2	KM491711
Morus notabilis	NC_027110.1	KP939360
Musa textilis	NC_022926.1	KF601567
Myriopteris lindheimeri	NC_014592.1	HM778032
Nageia nagi	NC_023120.1	AB830885
Najas flexilis	NC_021936.1	JX978472
Nandina domestica	NC_008336.1	DQ923117
Nasturtium officinale	NC_009275.1	AP009376
Nelumbo lutea	NC_015605.1	FJ754269
Nelumbo nucifera	NC_025339.1	KF009944
Neohouzeaua sp. Clark & Attigala 1712	NC_026963.1	KJ870995
Neololeba atra	NC_026964.1	KJ870996
Nerium oleander	NC_025656.1	KJ953907
Neyraudia reynaudiana	NC_024262.1	KF356392
Nicotiana sylvestris	NC_007500.1	AB237912
Nicotiana tabacum	-	Z00044.2
Nicotiana tabacum	NC_001879.2	-
Nicotiana tomentosiformis	NC_007602.1	AB240139
Nicotiana undulata	NC_016068.1	JN563929
Nothoceros aenigmaticus	NC_020259.1	KC285889
Nuphar advena	NC_008788.1	DQ354691
Nyholmiella obtusifolia	NC_026979.1	KP765733
Nymphaea alba	NC_006050.1	AJ627251
Nymphaea mexicana	NC_024542.1	KF753633

Oenothera argillicola	NC_010358.1	EU262887
Oenothera biennis	NC_010361.1	EU262889
Oenothera elata subsp. hookeri	NC_002693.2	-
Oenothera glazioviana	NC_010360.1	EU262890
Oenothera parviflora	NC_010362.1	EU262891
Olea europaea	NC_013707.2	GU228899
Olea europaea subsp. cuspidata	NC_015604.1	FN650747
Olea europaea subsp. europaea	NC_015401.1	FN997650
Olea europaea subsp. maroccana	NC_015623.1	FN998900
Olea woodiana subsp. woodiana	NC_015608.1	FN998901
Oligostachyum shiuyingianum	NC_024722.1	JX513423
Olimarabidopsis pumila JS2	NC_009267.1	AP009368
Olmeca reflexa	NC_026965.1	KJ870997
Olyra latifolia	NC_024165.1	KF515509
Oncidium hybrid cultivar	NC_014056.1	GQ324949
Oncinotis tenuiloba	NC_025657.1	KJ953908
Ophioglossum californicum	NC_020147.1	KC117178
Orobanche californica	NC_025651.1	HG515539
Orobanche crenata	NC_024845.1	HG515537
Orthotrichum rogeri	NC_026212.1	KP119739
Oryza australiensis	NC_024608.1	KJ830774
Oryza barthii	NC_027460.1	KM881634
Oryza glaberrima	NC_024175.1	KJ513090
Oryza glumipatula	NC_027461.1	KM881640
Oryza longistaminata	NC_027462.1	KM881641
Oryza meridionalis	NC_016927.1	JN005831
Oryza nivara SL10	NC_005973.1	AP006728
Oryza officinalis	NC_027463.1	KM881643
Oryza punctata	NC_027676.1	KM103375
Oryza rufipogon	NC_017835.1	JN005832
Oryza sativa	NC_027678.1	KM103382
Oryza sativa Indica Group	NC_008155.1	AY522329
Oryza sativa Japonica Group	NC_001320.1	-
Oryzopsis asperifolia	NC_027479.1	KM974744
Osmundastrum cinnamomeum	NC_024157.1	KF225592
Otatea acuminata	NC_026971.1	KJ871003
Pachycladon cheesemanii	NC_021102.1	JQ806762
Pachycladon enysii	NC_018565.1	JX205495
Pachyrhizus erosus	NC_026682.1	-
Paeonia obovata	NC_026076.1	KJ206533
Panax ginseng	NC_006290.1	AY582139
Panax notoginseng	NC_026447.1	KJ566590
Panax quinquefolius	NC_027456.1	KM088018
Panicum virgatum	NC_015990.1	HQ822121
Paphiopedilum armeniacum	NC_026779.1	KJ566307
Paphiopedilum niveum	NC_026776.1	KJ524105
Pariana campestris	NC_027491.1	KP319244
Pariana radiciflora	NC_026972.1	KJ871004
Parinari campestris	NC_024067.1	KJ414486
Paris verticillata	NC_024560.1	KJ433485
Parthenium argentatum	NC_013553.1	GU120098
Pastinaca pimpinellifolia	NC_027450.1	KM035850
Pelargonium alternans	NC_023261.1	KF240617
Pelargonium x hortorum	NC_008454.1	DQ897681
Pellia endiviifolia	NC_019628.1	JX827163
Pentactina rupicola	NC_016921.1	JQ041763
Pentalinon luteum	NC_025658.1	KJ953909
Penthorum chinense	NC_023086.1	JX436155
Petrosavia stellaris	NC_023356.1	KF482381
Phaenosperma globosum	NC_027480.1	KM974745
Phalaenopsis aphrodite subsp. formosana	NC_007499.1	AY916449
Phalaenopsis equestris	NC_017609.1	JF719062
Phalaenopsis hybrid cultivar	NC_025593.1	KJ944326
Phalaris arundinacea	NC_027481.1	KM974746
Pharus lappulaceus	NC_023245.1	KC311467
Pharus latifolius	NC_021372.1	JN032131
Phaseolus vulgaris	NC_009259.1	DQ886273
Phleum alpinum	NC_027482.1	KM974747
Phoenix dactylifera	NC_013991.2	GU811709
Phragmites australis	NC_022958.1	KF730315
Phyllostachys edulis	NC_015817.1	HQ337796
Phyllostachys nigra var. henonis	NC_015826.1	HQ154129
Phyllostachys propinqua	NC_016699.1	JN415113
Phyllostachys sulphurea	NC_024669.1	KJ722540
Physalis peruviana	NC_026570.1	KP295964
Physcomitrella patens	NC_005087.1	AP005672
Picea abies	NC_021456.1	HF937082
Picea morrisonicola	NC_016069.1	AB480556
Picea sitchensis	NC_011152.3	EU998739
Pinellia ternata	NC_027681.1	KR270823
Pinus gerardiana	NC_011154.4	EU998741

Pinus koraiensis	NC_004677.2	AY228468
Pinus krempfii	NC_011155.4	EU998742
Pinus lambertiana	NC_011156.4	EU998743
Pinus massoniana	NC_021439.1	KC427272
Pinus monophylla	NC_011158.4	EU998745
Pinus nelsonii	NC_011159.4	EU998746
Pinus strobus	NC_026302.1	KP099650
Pinus taeda	NC_021440.1	KC427273
Pinus taiwanensis	NC_027415.1	KP771703
Pinus thunbergii	NC_001631.1	D17510
Piper cenocladum	NC_008457.1	DQ887677
Piptochaetium avenaceum	NC_027483.1	KM974748
Pisum sativum	NC_014057.1	HM029370
Platanus occidentalis	NC_008335.1	DQ923116
Pleioblastus maculatus	NC_024723.1	JX513424
Poa palustris	NC_027484.1	KM974749
Podocarpus lambertii	NC_023805.1	KJ010812
Podocarpus totara	NC_020361.1	KC306742
Podococcus barteri	NC_027276.1	KR347117
Populus alba	NC_008235.1	AP008956
Populus balsamifera	NC_024735.1	KJ664927
Populus euphratica	NC_024747.1	KJ624919
Populus fremontii	NC_024734.1	KJ664926
Populus tremula	NC_027425.1	KP861984
Populus trichocarpa	NC_009143.1	EF489041
Praxelis clematidea	NC_023833.1	KF922320
Premna microphylla	NC_026291.1	KM981744
Primula poissonii	NC_024543.1	KF753634
Prinsepia utilis	NC_021455.1	KC571835
Prosopis glandulosa	NC_026683.1	-
Prunus kansuensis	NC_023956.1	KF990036
Prunus maximowiczii	NC_026981.1	KP760071
Prunus mume	NC_023798.1	KF765450
Prunus padus	NC_026982.1	KP760072
Prunus persica	NC_014697.1	HQ336405
Prunus yedoensis	NC_026980.1	KP760070
Pseudophoenix vinifera	NC_020364.1	JX088662
Pseudotsuga sinensis var. wilsoniana	NC_016064.1	AB601120
Psilotum nudum	NC_003386.1	AP004638
Pteridium aquilinum subsp. aquilinum	NC_014348.1	HM535629
Ptilidium pulcherrimum	NC_015402.1	HM222519
Puccinellia nuttalliana	NC_027485.1	KM974750
Pyrus pyrifolia	NC_015996.1	AP012207
Pyrus spinosa	NC_023130.1	HG737342
Quercus aliena	NC_026790.1	KP301144
Quercus aquifolioides	NC_026913.1	KP340971
Quercus rubra	NC_020152.1	JX970937
Quercus spinosa	NC_026907.1	KM841421
Raddia brasiliensis	NC_026966.1	KJ870998
Ranunculus macranthus	NC_008796.1	DQ359689
Ravenala madagascariensis	NC_022927.1	KF601568
Retrophyllum piresii	NC_024827.1	KJ617081
Rhazya stricta	NC_024292.1	KJ485849
Rheum palmatum	NC_027728.1	KR816224
Rhynchoryza subulata	NC_016718.1	JN415114
Ricinus communis	NC_016736.1	JF937588
Robinia pseudoacacia	NC_026684.1	-
Rosmarinus officinalis	NC_027259.1	KR232566
Sabal domingensis	NC_026444.1	KF928963
Saccharum hybrid cultivar SP80-3280	NC_005878.2	AE009947
Saccharum officinarum	NC_006084.1	AP006714
Salicornia bigelovii	NC_027226.1	KJ629117
Salicornia brachiata	NC_027224.1	KJ629115
Salicornia europaea	NC_027225.1	KJ629116
Salix interior	NC_024681.1	KJ742926
Salix purpurea	NC_026722.1	KP019639
Salix suchowensis	NC_026462.1	KM983390
Salvia miltiorrhiza	NC_020431.1	JX312195
Sanionia uncinata	NC_025668.1	KM111545
Sapindus mukorossi	NC_025554.1	KM454982
Saracha punctata	NC_026694.1	KP280050
Sarocalamus faberi	NC_024713.1	JX513414
Sartidia dewinteri	NC_027147.1	KJ819550
Sartidia perrieri	NC_027146.1	KJ819549
Schefflera delavayi	NC_022813.1	KC456166
Schwalbea americana	NC_023115.1	HG738866
Scrophularia takesimensis	NC_026202.1	KM590983
Scutellaria baicalensis	NC_027262.1	KR233163
Secale cereale	NC_021761.1	KC912691
Sedum sarmentosum	NC_023085.1	JX427551
Sedum takesimense	NC_026065.1	KF954541

Selaginella moellendorffii	NC_013086.1	FJ755183
Sesamum indicum	NC_016433.2	-
Seseli montanum	NC_027451.1	KM035851
Setaria italica	NC_022850.1	KF646538
Silene chalcedonica	NC_023359.1	KF527886
Silene conica	NC_016729.1	JF715054
Silene conoidea	NC_023358.1	KF527885
Silene latifolia	NC_016730.1	JF715055
Silene noctiflora	NC_016728.1	JF715056
Silene paradoxa	NC_023360.1	KF527887
Silene vulgaris	NC_016727.1	JF715057
Sinopodophyllum hexandrum	NC_027732.1	KR779994
Solanum bulbocastanum	NC_007943.1	DQ347958
Solanum cheesmaniae	NC_026876.1	KP117020
Solanum chilense	NC_026877.1	KP117021
Solanum galapagense	NC_026878.1	KP117022
Solanum habrochaites	NC_026879.1	KP117023
Solanum lycopersicum	AC_000188.1	AM087200
Solanum lycopersicum	NC_007898.3	DQ347959
Solanum neorickii	NC_026880.1	KP117025
Solanum pennellii	NC_024584.1	HG975452
Solanum peruvianum	NC_026881.1	KP117026
Solanum pimpinellifolium	NC_026882.1	KP117027
Solanum tuberosum	NC_008096.2	DQ386163
Sorghum bicolor	NC_008602.1	EF115542
Sorghum timorense	NC_023800.1	KF998272
Spinacia oleracea	NC_002202.1	-
Spirodela polyrhiza 7498	NC_015891.1	JN160603
Sporobolus maritimus	NC_027650.1	KP176438
Stangeria eriopus	NC_026041.1	JX416858
Stipa hymenoides	NC_027464.1	KM974729
Stockwellia quadrifida	NC_022414.1	KC180807
Syntrichia ruralis	NC_012052.1	FJ546412
Taiwania cryptomerioides	NC_016065.1	AP012266
Taiwania flousiana	NC_021441.1	KC427274
Tamarindus indica	NC_026685.1	-
Taxus mairei	NC_020321.1	JN867586
Tectona grandis	NC_020098.1	HF567869
Tectona grandis	-	HF567870.1
Tectona grandis	-	HF567871.1
Tetracentron sinense	NC_021425.1	KC608752
Tetraphis pellucida	NC_024291.1	KJ817846
Thalictrum coreanum	NC_026103.1	KM206568
Thamnocalamus spathiflorus	NC_024724.1	JX513425
Theobroma cacao	NC_014676.2	HQ244500
Thysanolaena latifolia	NC_025238.1	KJ920236
Torreyochloa pallida	NC_027486.1	KM974752
Trachelium caeruleum	NC_010442.1	EU090187
Trifolium aureum	NC_024035.1	KC894708
Trifolium boissieri	NC_025743.1	KJ788284
Trifolium glanduliferum	NC_025744.1	KJ788285
Trifolium grandiflorum	NC_024034.1	KC894707
Trifolium meduseum	NC_024166.1	KJ476730
Trifolium repens	NC_024036.1	KC894706
Trifolium strictum	NC_025745.1	KJ788292
Trifolium subterraneum	NC_011828.1	EU849487
Trigonobalanus doichangensis	NC_023959.1	KF990556
Trillium cuneatum	NC_027185.1	KR135077
Trillium decumbens	NC_027282.1	KR534612
Trillium maculatum	NC_027738.1	KR780075
Trillium tschonoskii	NC_027739.1	KR780076
Trisetum cernuum	NC_027487.1	KM974753
Trithuria inconspicua	NC_020372.1	HE963749
Triticum aestivum	NC_002762.1	AB042240
Triticum macha	NC_025955.1	LC005978
Triticum monococcum	NC_021760.1	KC912690
Triticum timopheevii	NC_024764.1	KJ614410
Triticum turgidum	NC_024814.1	KJ614397
Triticum urartu	NC_021762.1	KC912693
Trochodendron aralioides	NC_021426.1	KC608753
Typha latifolia	NC_013823.1	GU195652
Utricularia gibba	NC_021449.1	KC997777
Utricularia macrorhiza	NC_025653.1	HG803177
Vaccinium macrocarpon	NC_019616.1	JQ757046
Vanilla planifolia	NC_026778.1	KJ566306
Veratrum patulum	NC_022715.2	KF437397
Vicia sativa	NC_027155.1	KJ850242
Vigna angularis	NC_021091.1	AP012598
Vigna radiata	NC_013843.1	GQ893027
Vigna unguiculata	NC_018051.1	JQ755301
Viola seoulensis	NC_026986.1	KP749924

Vitis rotundifolia	NC_023790.1	KF976463
Vitis vinifera	NC_007957.1	DQ424856
Viviania marifolia	NC_023259.1	KF240615
Welwitschia mirabilis	NC_010654.1	EU342371
Wisteria floribunda	NC_027677.1	KM103376
Wolffia australiana 7733	NC_015899.1	JN160605
Wolffiella lingulata 7289	NC_015894.1	JN160604
Wollemia nobilis	NC_027235.1	KP259800
Xerophyllum tenax	NC_027158.1	KM078035
Yushania levigata	NC_024725.1	JX513426
Zamia furfuracea	NC_026040.1	JX416857
Zea mays	NC_001666.2	X86563
Zingiber spectabile	NC_020363.1	JX088661
Zizania aquatica	NC_026967.1	KJ870999

Supplementary Table S4. Performance of plant core barcode in metabarcoding studies. Diagnostic value (percent species identified) of the core land plant barcode (*rbcL* + *matK*) reported by large barcoding studies (comparing >50 diverse species). Maximum estimate is shown when more than one method of species diagnosis was applied. *Approximate value taken from Figure 4

Reference	No. of species	Percent diagnosis
1. Burgess et al. (2011)	436	93
2. China Plant BOL Group (2011)	1757	53
3. Clement and Donoghue (2012)	112	53
4. De Vere et al. (2012)	1143	75
5. Dong et al. (2015)	490	71
6. Gere et al (2013)	58	61
7. CBOL Plant Working Group (2011)	550	72
8. Huang et al. (2015)	655	48
9. Kim et al. (2014)	84	86
10. Kuzmina et al. (2012)	286	54
11. Li et al. (2012)	1319	55
12. Little et al. (2013)	145	46
13. Liu et al. (2014)	149	40
14. Mangka et al. (2013)	108	97
15. Pang et al. (2011)	52	96
16. Pei et al. (2015)	896	73
17. Saarela et al. (2013)	312	56
18. Yan et al. (2015)	173	30
19. Yu et al. (2011)	88	70*

<u>**Full references**</u>

1. Burgess, K. S. *et al.* Discriminating plant species in a local temperate flora using the *rbcL* + *matK* DNA barcode. *Methods Ecol. Evol.* **2,** 333-340 (2011).
2. China Plant BOL Group. Comparative analysis or a large dataset indicated that internal transcribed spacer (ITS) should be incorporated in the core barcode for seed plants. *Proc. Natl. Acad. Sc.i USA.* **108,** 19641-19646 (2011).
3. Clement, W. L., Donoghue, M. J. Barcoding success as a function of phylogenetic relatedness in Viburnum, a clade of woody angiosperms. *BMC Evol. Biol.* **12,** 73 (2012).
4. De Vere, N. *et al*. DNA barcoding the native flowering plants and conifers of Wales. *Plos ONE* **7,** e37945 (2012).
5. Dong, W. *et al*. *ycf*1, the most promising plastid DNA barcode of land plants. *Sci. Rep.* **5,** 8348 (2015).
6. Gere, J. *et al*. Incorporating *trnH-psbA* to the core DNA barcodes improves significantly species discriminaton within southern African Combretaceae. *ZooKeys* **365,** 129-147 (2013).
7. CBOL Plant Working Group. A DNA barcode for land plants. *Proc. Natl. Acad. Sci. USA.* **106,** 12794-12797 (2011).
8. Huang, X. –C., Ci, X. –Q., Conran, J. G., Li, J. Application of DNA barcodes in Asian tropical trees – a case study from Xishuangbanna nature reserve, Southwest China. *PloS ONE* **10,** e0129295 (2015).
9. Kim, H. M., Oh, S. –H., Bhandari, G. S., Kim, C. –S., Park, C. -W. DNA barcoding of Orchidaceae in Korea. *Mol. Ecol. Res.* **14,** 499-507 (2014).
10. Kuzmina, M. L., Johnson, K. L., Barron, H. R., Hebert, P. D. N. Identification of the vascular plants of Churchill, Manitoba, using a DNA barcode library. *BMC Ecol.* **12,** 25 (2012).
11. Li, C. P., Yu, Z. G., Han, G. S., Chu, K. H. Analyzing multi-locus plant barcoding datasets with a composition vector method based on adjustable weighted distance. *PloS ONE* **7,** 42154 (2012).
12. Little, D. P., Knopf, P., Schulz, C. DNA barcode identification of Podocarpaceae – the second largest conifer family. *PloS ONE* **8,** e81008 (2013).
13. Liu, J. *et al.* Identification of species in the angiosperm family Apiaceae using DNA barcodes. *Mol. Ecol. Res.* **14,** 1231-1238 (2014).
14. Mankga, L. T., Yessoufou, K., Moteetee, A. M., Daru, B. H., van der Bank, M. Efficacy of the core DNA barcodes in identifying processed and poorly conserved plant materials commonly used in South African traditional medicine. *ZooKeys* **365,** 215-233 (2013).
15. Pang, X. *et al.* Applying plant DNA barcodes for Rosaceae species identification. *Cladistics* **27,** 165-170 (2011).
16. Pei, N. *et al*. Closely-related taxa influence woody species discrimination via DNA barcoding: evidence from global dynamics plots. *Sci. Rep.* **5,** 15127 (2015).
17. Saarela, J. M., Sokoloff, P. C., Gillespie, L. J., Consaul, L. L., Bull, R. D. DNA barcoding the Canadian arctic flora: core plastid barcodes (*rbcL* + *matK*) for 490 vascular plant species. *PloS ONE* **8,** e77982 (2013).
18. Yan, L. –J. *et al*. DNA barcoding of *Rhododendron* (Ericaceae), the largest Chinese plant genus in biodiversity hotspots of the Himalaya-Hengduan Mountains. *Mol. Ecol. Res.* **15,** 932-944 (2015).
19. Yu, W. B. *et al.* DNA barcoding of *Pedicularis* Linn. (Orobanchaceae): evaluating four universal barcode loci in a large and hemiparasitic genus. *J. Syst. Evol.* **49,** 425-437 (2011).

Supplementary Table S5. Specimens used for conventional and Next Generation reference barcoding. List of plant species collected from the Murraylands and Waite Arboretum, South Australia for which *de novo* barcoding was performed for *rbc*L and *mat*K via conventional Sanger sequencing, and the Illumina V3 MiSeq Platform using the Extended Unidirectional Sequencing (EUS) and Sonication MicroAssembly (SMA) methods. Key: - = reaction not performed; *X* = reaction failed.

					Recovered sequence length (base pairs)						
					rbcL barcode region		matK barcode region			SMA gene-length	
**Accession Number**	**Species**	**Collection Site**	**Latitude**	**Longitude**	**EUS**	**Sanger**	**SMA**	**Sanger**	**Diff.**	**rbcL**	**matK**
M75	*Acacia nyssophylla*	Murraylands	-34.538481	139.591213	553	553	898	867	31	-	-
M63	*Ajuga iva*	Murraylands	-34.531619	139.585348	553	553	904	879	25	-	-
M53	*Alectryon oleifolius*	Murraylands	-34.460922	139.472026	553	553	898	869	29	-	-
M38	*Amyema miquelli*	Murraylands	-34.461621	139.472463	553	553	910	865	45	-	-
M74	*Amyema preissii*	Murraylands	-34.538481	139.591213	553	553	901	865	36	-	-
529	*Araucaria bidwillii*	Waite Arboretum	34° 58' S	138° 37' E	-	-	898	-	-	-	-
521	*Araucaria cunninghamii*	Waite Arboretum	34° 58' S	138° 37' E	-	-	X	-	-	-	-
M80	*Asphodelus fistulosus*	Murraylands	-34.381057	139.531638	553	553	916	880	36	-	-
X94	*Asteridea athrixioides*	Murraylands	-34.461328	139.472276	553	553	904	874	30	-	-
M62	*Atriplex stipitata*	Murraylands	-34.531619	139.585348	553	553	-	913	-	-	-
M5	*Austrostipa drummondii*	Murraylands	-34.461294	139.472163	553	553	898	888	10	1333	1471
M22	*Austrostipa drummondii*	Murraylands	-34.460958	139.471688	553	553	898	888	10	-	-
M2	*Austrostipa elegantissima*	Murraylands	-34.461097	139.472694	553	553	898	888	10	1333	1471
M28	*Austrostipa elegantissima*	Murraylands	-34.461045	139.472189	X	553	898	858	40	X	X
M59	*Austrostipa eremophila*	Murraylands	-34.531619	139.585348	553	553	898	886	12	1333	1471
M4	*Austrostipa hemipogon*	Murraylands	-34.461243	139.472235	553	553	898	888	10	1333	1471
M44	*Austrostipa puberula*	Murraylands	-34.461206	139.472078	553	553	898	868	30	1333	X
M43	*Austrostipa scabra falcata*	Murraylands	-34.461206	139.472078	553	553	898	887	11	X	X
M32	*Austrostipa* sp.	Murraylands	-34.461045	139.472189	553	553	898	889	9	-	-
M1	*Austrostipa* sp.	Murraylands	-34.461045	139.472189	553	553	898	866	32	-	-
M81	*Austrostipa* sp.	Murraylands	-34.380974	139.533341	553	553	898	887	11	-	-
M16	*Austrostipa* sp.	Murraylands	-34.461294	139.471973	553	553	898	893	5	-	-
X88	*Austrostipa* sp.	Murraylands	-34.374865	139.530738	553	553	898	898	0	-	-
A114	*Brachyscome ciliaris*	Murraylands	-34.456677	139.478655	553	553	892	852	40	-	-
X95	*Brachyscome ciliaris*	Murraylands	-34.461433	139.472332	553	553	-	856	-	-	-
M20	*Brachyscome ciliaris*	Murraylands	-34.461227	139.471778	553	553	892	862	30	-	-
M48	*Brassica tournefortii*	Murraylands	-34.461234	139.47243	553	553	-	790	-	-	-
X84	*Bromus madritensis*	Murraylands	-34.538808	139.590432	553	553	-	856	-	1333	X
M11	*Bupleurum semicompositum*	Murraylands	-34.461294	139.471973	553	553	889	861	28	-	-
X93	*Bupleurum semicompositum*	Murraylands	-34.461433	139.472332	553	553	889	862	27	-	-
1022a	*Callitris rhomboidea*	Waite Arboretum	34° 58' S	138° 37' E	-	-	X	-	-	-	-
M68	*Calotis hispidula*	Murraylands	-34.531488	139.585158	553	553	892	858	34	-	-

B116	*Carrichtera annua*	Murraylands	-34.461541	139.473541	✗	553	883	849	34	-	-
M83	*Convolvulus angustissimus* ssp. *peninsularum*	Murraylands	-34.38073	139.531513	553	553	904	878	26	-	-
M39A	*Crassula colorata*	Murraylands	-34.461045	139.472189	553	553	-	-	-	-	-
563	*Cupressus glabra*	Waite Arboretum	34° 58' S	138° 37' E	-	-	✗	-	-	-	-
1024	*Cupressus jiangeensis*	Waite Arboretum	34° 58' S	138° 37' E	-	-	✗	-	-	-	-
M51	*Dissocarpus paradoxus*	Murraylands	-34.460927	139.472364	553	553	898	887	11	1333	1461
M65	*Einadia nutans* ssp. *nutans*	Murraylands	-34.531488	139.585158	553	553	898	889	9	1333	1470
M41	*Enchylaena tomentosa*	Murraylands	-34.461179	139.472078	553	553	-	889	-	✗	1461
M46	*Enchylaena tomentosa* var. *tomentosa*	Murraylands	-34.461233	139.472259	553	553	898	856	42	1333	1470
X102	*Eremophila glabra*	Murraylands	-34.464857	139.475058	553	553	904	873	31	-	-
M56B	*Eremophila scoparia*	Murraylands	-34.476497	139.438005	553	553	904	896	8	-	-
M23	*Eriochiton sclerolaenoides*	Murraylands	-34.460958	139.471688	553	553	-	867	-	1333	1461
M8	*Eriochiton sclerolaenoides*	Murraylands	-34.46137	139.472157	✗	553	✗	889	-	1333	1461
A107	*Eriochiton sclerolaenoides*	Murraylands	-34.456677	139.478655	553	553	898	868	30	-	-
M26	*Eriochiton sclerolaenoides*	Murraylands	-34.461419	139.47212	553	553	898	860	38	1333	✗
A110	*Erodium crinitum*	Murraylands	-34.456677	139.478655	553	553	-	872	-	-	-
X91	*Erodium crinitum*	Murraylands	-34.461328	139.472276	✗	553	-	894	-	-	-
M69	*Euphorbia drummondii*	Murraylands	-34.531648	139.585179	553	553	904	875	29	-	-
M29	*Festuca* or *Vulpia* sp.	Murraylands	-34.461045	139.472189	553	553	898	860	38	-	-
X96	*Geijera linearifolia*	Murraylands	-34.460979	139.471614	553	553	898	866	32	-	-
M73	*Goodenia pinnatifida*	Murraylands	-34.538668	139.590656	553	553	904	874	30	-	-
A112	*Goodenia pusilliflora*	Murraylands	-34.456677	139.478655	553	553	904	877	27	-	-
X90	*Goodenia pusilliflora*	Murraylands	-34.461328	139.472276	553	553	904	902	2	-	-
X85	*Herniaria cinerea*	Murraylands	-34.461711	139.472935	553	553	898	867	31	-	-
M78	*Hordeum glaucum*	Murraylands	-34.538569	139.590701	553	553	898	860	38	-	-
X92	*Hyalosperma semisterile*	Murraylands	-34.461328	139.472276	553	553	916	885	31	-	-
A111	*Hyalosperma semisterile*	Murraylands	-34.456677	139.478655	553	553	916	886	30	-	-
M12	*Hypochaeris radicata*	Murraylands	-34.461294	139.471973	✗	553	904	904	0	-	-
1285	*Juniperus excelsa*	Waite Arboretum	34° 58' S	138° 37' E	-	-	✗	-	-	-	-
X86	*Maireana georgei*	Murraylands	-34.538201	139.595721	553	553	-	847		1333	✗
M45	*Maireana lobiflora*	Murraylands	-34.461252	139.472084	553	553	898	888	10	1333	1461
M36	*Maireana* sp.	Murraylands	-34.461166	139.472206	553	553	-	855	-	-	-
M39B	*Malva parviflora*	Murraylands	-34.461442	139.472332	553	553	-	✗	-	-	-
M72	*Marrubium vulgare*	Murraylands	-34.538546	139.590425	553	553	904	875	29	-	-
M60	*Medicago minima*	Murraylands	-34.531619	139.585348	553	553	856	826	30	-	-
M54	*Melaleuca lanceolata*	Murraylands	-34.461426	139.472555	553	553	895	884	11	-	-
M56A	*Mesembryanthemum crystallinum*	Murraylands	-34.539971	139.597051	553	553	898	898	0	-	-
M9	*Moraea setifolia*	Murraylands	-34.461311	139.472139	553	553	922	883	39	-	-
M40	*Moraea setifolia*	Murraylands	-34.461045	139.472189	553	553	922	884	38	-	-
M52	*Myoporum parvifolium*	Murraylands	-34.460922	139.472026	553	553	904	875	29	-	-
M77	*Nicotiana glauca*	Murraylands	-34.538568	139.59091	553	553	898	890	8	-	-
A109	*Nicotiana goodspeedii*	Murraylands	-34.459823	139.470087	553	553	898	889	9	-	-

M37	*Nicotiana goodspeedii*	Murraylands	-34.461252	139.472084	553	553	898	889	9	-	-
M15	*Oxalis perennans*	Murraylands	-34.461294	139.471973	553	553	-	913	-	-	-
B118	*Pimelea micrantha*	Murraylands	-34.461541	139.473541	✕	553	-	✕	-	-	-
527	*Pinus nigra*	Waite Arboretum	34° 58' S	138° 37' E	-	-	✕	-	-	-	-
538	*Podocarpus elatus*	Waite Arboretum	34° 58' S	138° 37' E	-	-	✕	-	-	-	-
M13	*Ptilotus seminudus*	Murraylands	-34.461294	139.471973	553	553	892	844	48	-	-
M18	*Ptilotus spathulatus*	Murraylands	-34.461219	139.471837	553	553	892	849	43	-	-
X100	*Rhagodia candolleana*	Murraylands	-34.464857	139.475058	553	553	-	858	-	1333	1470
X97	*Rhagodia crassifolia*	Murraylands	-34.464857	139.475058	553	553	898	874	24	1333	1461
X99	*Rhagodia crassifolia* X *R. candolleana*	Murraylands	-34.464857	139.475058	553	553	898	875	23	✕	1470
M49	*Rhagodia* hybrid	Murraylands	-34.460852	139.472201	553	553	898	850	48	1333	1470
M67	*Rhagodia parabolica*	Murraylands	-34.531488	139.585158	553	553	898	860	38	1333	1470
M64	*Rhagodia parabolica*	Murraylands	-34.531488	139.585158	553	553	-	898	-	1333	1494
M76	*Rhagodia* sp.	Murraylands	-34.538481	139.591213	553	553	-	871	-	-	-
X101	*Rhagodia spinescens*	Murraylands	-34.464857	139.475058	553	553	-	845	-	1333	1470
X98	*Rhagodia spinescens*	Murraylands	-34.464857	139.475058	553	553	-	849	-	1333	1470
M10	*Rostraria cristata*	Murraylands	-34.461294	139.471973	553	553	-	✕	-	-	-
M33	*Rostraria cristata*	Murraylands	-34.461145	139.47233	553	553	-	✕	-	-	-
M42	*Rytidosperma caespitosa*	Murraylands	-34.461206	139.472078	553	553	-	801	-	1333	1471
M3	*Rytidosperma caespitosa*	Murraylands	-34.461045	139.472189	553	553	898	872	26	1333	1471
M82	*Rytidosperma caespitosa*	Murraylands	-34.380974	139.533341	553	553	898	886	12	1333	1471
M61	*Rytidosperma caespitosa*	Murraylands	-34.531619	139.585348	553	553	-	898	-	1333	1471
M7	*Rytidosperma caespitosa*	Murraylands	-34.461319	139.472236	553	553	-	856	-	1333	✕
M27	*Rytidosperma caespitosa*	Murraylands	-34.461045	139.472189	553	553	-	881	-	1333	1470?
M17	*Rytidosperma* sp.	Murraylands	-34.461219	139.471837	✕	553	-	885	-	-	-
M57	*Schismus barbatus*	Murraylands	-34.53216	139.586191	553	553	-	845	-	1333	✕
M71	*Schismus barbatus*	Murraylands	-34.531648	139.585179	553	553	898	893	5	1333	✕
M70	*Schismus barbatus*	Murraylands	-34.531648	139.585179	553	553	898	898	0	1333	✕
M34	*Sclerolaena patenticuspis*	Murraylands	-34.461072	139.472527	553	553	898	867	31	1333	1461
X87	*Senecio spanomerus*	Murraylands	-34.381012	139.536642	553	553	895	865	30	-	-
M35	*Senna artemisioides* ssp. *filifolia*	Murraylands	-34.461072	139.472527	553	553	898	892	6	-	-
M79	*Sida corrugata*	Murraylands	-34.540566	139.581067	553	553	889	857	32	-	-
M58	*Silene apetala*	Murraylands	-34.531619	139.585348	553	553	904	874	30	-	-
A113	*Silene apetala*	Murraylands	-34.456677	139.478655	553	553	-	✕	-	-	-
M30	*Sonchus oleraceus*	Murraylands	-34.461045	139.472189	553	553	904	874	30	-	-
M66	*Sonchus oleraceus*	Murraylands	-34.531488	139.585158	553	553	904	874	30	-	-
X104	*Sonchus oleraceus*	Murraylands	-34.461243	139.472235	553	553	-	903	-	-	-
M14	*Sonchus oleraceus*	Murraylands	-34.461294	139.471973	553	553	-	-	-	-	-
M21	*Sonchus* sp.	Murraylands	-34.461173	139.471629	553	553	904	903	1	-	-
A105	*Teucrium racemosum*	Murraylands	-34.542992	139.591191	553	553	904	874	30	-	-
M47	*Thysanotus baueri*	Murraylands	-34.461232	139.472301	553	553	916	905	11	-	-
M55	unidentified Camphorosmeae Amaranthaceae	Murraylands	-34.461979	139.472338	553	553	898	841	57	1333	1461

M50	unidentified Camphorosmeae Amaranthaceae	Murraylands	-34.460927	139.472364	553	553	898	898	0	-	-
M19	unidentified Gnaphalieae Asteraceae	Murraylands	-34.461227	139.471778	553	553	904	865	39	-	-
A108	unidentified Gnaphalieae Asteraceae	Murraylands	-34.456677	139.478655	553	553	904	871	33	-	-
M25	*Velleia arguta*	Murraylands	-34.460978	139.471702	553	553	904	899	5	-	-
M24	*Vittadinia cuneata*	Murraylands	-34.460978	139.471702	553	553	892	867	25	-	-
B115	*Wurmbea dioica*	Murraylands	-34.461541	139.473541	553	553	898	863	35	-	-
X103	*Zygophyllum angustifolium*	Murraylands	-34.464857	139.475058	553	553	898	868	30	-	-
X89	*Zygophyllum apiculatum*	Murraylands	-34.379439	139.540514	553	553	898	867	31	-	-
M6	*Zygophyllum aurantiacum*	Murraylands	-34.531619	139.585348	X	553	898	866	32	-	-
M31	*Zygophyllum aurantiacum*	Murraylands	-34.461045	139.472189	553	553	898	874	24	-	-

Supplementary Table S6. **Primers for gene-length barcodes.** Primer pairs designed to generate full-length gene amplicons for *rbc*L and *mat*K from Amaranthaceae (prefix Caryo) and Poaceae (prefix Poa) samples.

Primer	Direction	Sequence
rbcLa_f*	forward	ATG TCA CCA CAA ACA GAG ACT AAA GC
rbcL_UR2d	reverse	CTT CAC AAG CNG CRG CTA GTT C
matK_CaryoFwd1d	forward	GAA TTC CAA GGA TAT WTA GAA C
matK_CaryoRev1d	reverse	CAT CAT TGG CCA AAT YAT GRA TAC
matK_PoaFwd1	forward	ATT CGA AGG GTA TTC AGA AAA AC
matK_Poa1554R	reverse	GTT CAC CAG GTC ATT GAT ACG G

*Levin, R. A. *et al.* (2003) Family-level relationships of Onagraceae based on chloroplast rbcL and ndhF data. *Am. J. Bot.* **90,** 107–115.
